# Switchable Ultralong Chiral Signal Transmission and Gate Tunability in Organic Chiral Semiconductor

**DOI:** 10.34133/research.1306

**Published:** 2026-06-15

**Authors:** Renjie Hu, Xiaoying Niu, Xiangqian Lu, Shilin Li, Yuan Yu, Xiangping Zhao, Zhiwei Xiang, Kepeng Song, Ki Tae Nam, Kun Gao, Wei Qin

**Affiliations:** ^1^ School of Physics, State Key Laboratory of Crystal Materials, Shandong University, Jinan 250100, China.; ^2^ Department of Materials Science and Engineering, Seoul National University, Seoul 08826, Republic of Korea.; ^3^ School of Chemistry and Chemical Engineering, Shandong University, Jinan 250100, China.

## Abstract

Chiral structures have substantial potential for spin information utilization because of their distinctive spatial symmetry-breaking features and the consequent strong spin–charge correlation. One of the limitations in the development of chiral spintronics for both theoretical and practical applications is the limited range of chiral-related signal transmission, which is only tens of nanometers and is usually determined by the conductivity of the chiral materials and device applicability. In addition, the role of circularly polarized light, an important chiral parameter, in chiral signal transmission has been typically neglected. In this study, targeted chiral induction was conducted using the semiconductor polymer PCDTPT ([4-(4,4-dihexadecyl-4*H*-cyclopenta[1,2-b:5,4-b′]dithiophen-2-yl)-alt-[1,2,5]thiad-iazolo [3,4-c] pyridine]), which was further used to fabricate a field-effect transistor device. In a dark environment, the chiral-related signal could be transmitted up to 10 μm with an external magnetic field. Through the synergistic effects of spin-selective transition and chiral spin filtering under illumination, the chiral signal expression was fundamentally switched to another form, and the transmission distance was notably increased to the millimeter scale. In addition, owing to the flexible external field tunability (gate voltage, temperature, and polarized light) in multiple scenarios, the chiral signals reflected detailed dynamic changes. The achievement of long-distance chiral signal transmission and circularly polarized light-induced signal conversion and extension is expected to promote the development of chiral spintronics for both theoretical and practical applications.

## Introduction

Chiral structures have drawn widespread attention in recent years as distinctive spatially symmetry-broken structures. This is due not only to its high efficiency and enantiomeric characteristics in molecular recognition and catalysis [1–5] and chiral–optical interactions [6–12] but also to its unique charge–spin properties [13–17]. Typically, the spins of the electrons can be locked in the direction perpendicular to their momentum in the system with the spin Hall effect and Edelstein effect [18–22], resulting in a homogeneous spin polarization oriented perpendicularly to the charge current flow [23–27]. By manipulating the axial direction of chirality and the orientation of the current, the singular spin-dependent Hall effect can be controlled, as the direction of the spin polarization is locked to be collinear with the direction of momentum [16,28–30]. It offers a novel perspective for the conversion between spin and charge current in chiral structures.

Within chiral structures, the chiral-induced spin selectivity (CISS) is expected to maintain spin polarization efficiently in the transport direction [16,29]. Meanwhile, the electron transport process and spin dynamics inside chiral structures have also garnered substantial attention [13,17,31–33]. In most cases, chiral spin based on vertical heterojunctions is a direct and commonly used configuration to study spin transport [32,34]. The chiral structure can lead to a pure spin current selectivity [30,35], which further verifies the special spin–momentum locking in chiral structures. Furthermore, utilizing the spin pumping, the inverse effect of the CISS effect in chiral polymer/Py heterojunctions was detected, indicating that the spin current flowing along the chiral axis can produce an equivalent charge current [16]. Therefore, although the concept of utilizing chiral structures to study the spin property has been proposed for many years, the distance of chiral-related signal transmission is usually limited to the nanoscale, regardless of whether magnon or electron is the spin propagation carrier [32,34,36]. The loss of interchain (between fibers) chirality–spin signal transmission is a key factor leading to the overall shorter transmission distance. In addition, the gate-field effect on chiral-dependent spin transport has not been sufficiently explored. Long-distance transmission is expected to advance chiral spintronics.

Herein, the chiral fiber width of approximately 3 nm will be beneficial for the transmission of chiral signals between fibers. An oriented chiral semiconductor film with excellent mobility and a suitable chiral nanofiber size was obtained experimentally. Chiral field-effect transistors (FETs) exhibit a specific chiral-dependent transport. Through the synergistic effects of spin-polarized injection and chiral spin filtering, the transport distance of the chiral signal can be substantially increased up to the millimeter level, which is the largest magnitude to date. Furthermore, mirror-symmetric chiral magnetoconductance (CMC) can be observed when the transported charge carriers change from electrons to holes. For chiral R-type (S-type) devices, the CMC signal can be enhanced (weakened) when switching the external magnetic field from negative to positive. In addition, the gate could effectively further enhance the chiral signal transport in chiral organic field-effect transistors (OFETs).

## Results

### Basic characterization of the OFET devices and the CMC signal

**Fig. 1.**
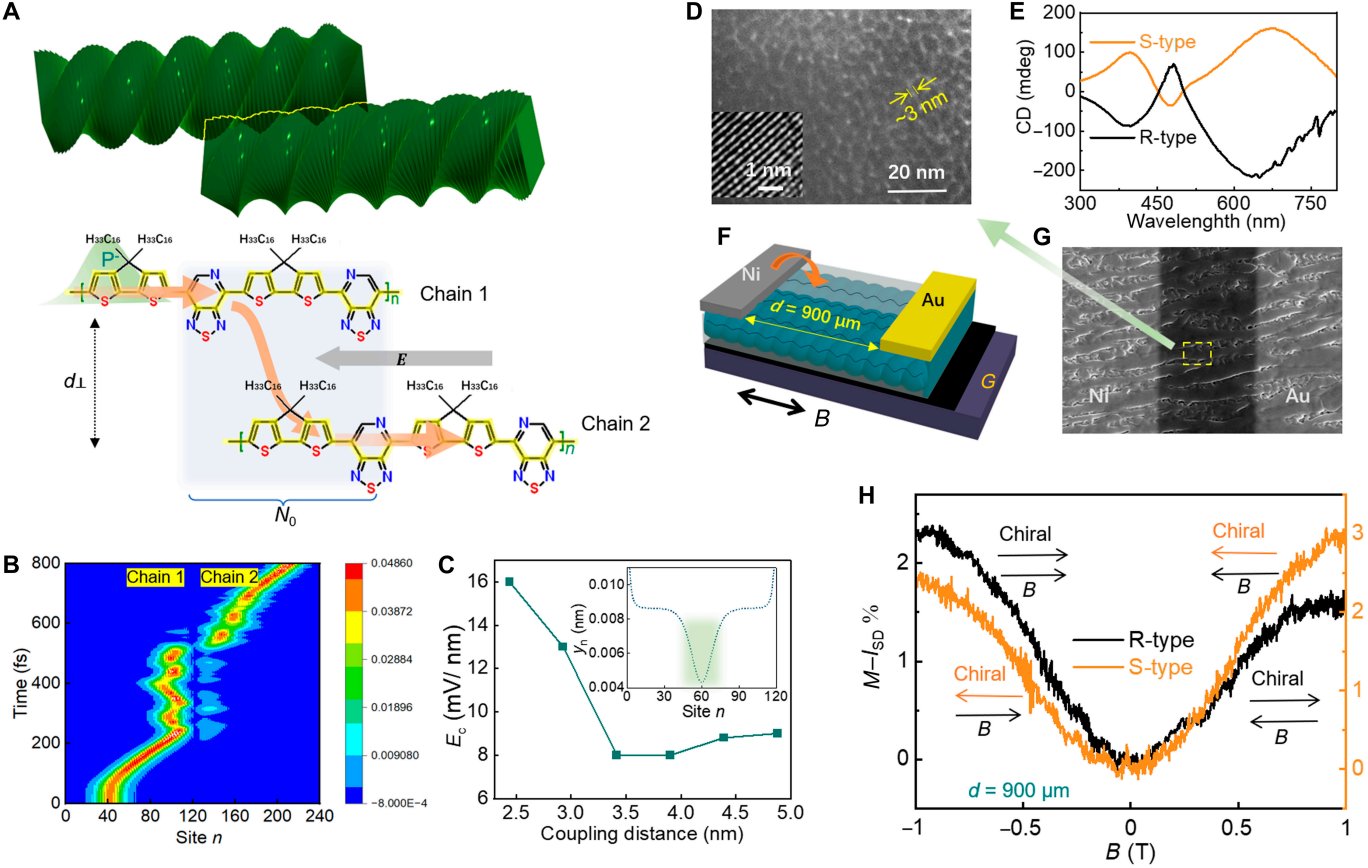
The tight-binding model analysis and basic characteristics of chiral-related spin transport. (A) Schematic of chiral polymers and interchain coupling model. (B) The polaron migration dynamics along the chains, where we show the evolution of the net charge density $q_{n}=e\left(\rho_{n,n}-1\right)$ [$\rho_{n,n}$ denotes the density matrix defined as Eq. (S9)]. The result with *N*_0_ = 30 and the electric field $E_{0}=7\;\text{mV}/\text{nm}$. (C) Correlation of the minimum critical electric field (*E*_c_) with the coupling distance between molecular chains, where the inset shows the lattice displacement order parameter ($y_{n}$) of the polaron formed in chain 1, and the rectangular shaded area indicates the polaron width. (D) The high-resolution transmission electron microscopy (HRTEM) of the chiral nanofiber network; a robust lattice structure is built inside the thin film (insert). (E) The circular dichroism (CD) spectrum of chiral polymer. (F) The schematic diagram of a field-effect transistor (FET) with asymmetrical electrodes (Ni and Au). (G) Scanning electron microscopy (SEM) of the chiral polymer surface with evaporated electrodes. (H) The chiral magnetoconductance (CMC) signal, *M–I*_SD_ = [*I*_SD_(*B*) − *I*_SD_(0)]/*I*_SD_(0) under multiple cycles of external magnetic fields for R/S-chiral-type devices under photoexcitation; the laser wavelength is 793 nm; *V*_G_ = −3 V and *V*_SD_ = −0.05 V.

By constructing 2 PCDTPT ([4-(4,4-dihexadecyl-4*H*-cyclopenta[1,2-b:5,4-b′]dithiophen-2-yl)-alt-[1,2,5]thiad-iazolo [3,4-c] pyridine]) polymer chains and analyzing the interchain transport of polarons theoretically (Fig. 1A). The results demonstrate that the carriers exhibit an optimized transport mode, with a coupling distance of ~3.5 nm between fiber chains (Fig. 1B and C, see Methods, and Figs. S1 to S3). In addition, the localization width of the polaron is comparable to such a distance. If the fiber width is about 3.5 nm, it will provide an excellent platform for chiral spin transmission between fibers. As a result, experimentally, the stepwise chiral induction method, combined with a thermally assisted strategy, was systematically applied for making suitable chiral fiber size. By adjusting the chiral inducer ratio and temperature difference in the solution, reasonable chiral structures, with a fiber width of approximately 3 nm and a clear lattice structure for the signal fiber, were obtained. These structures were comparable to those simulated (Fig. 1D; detailed information about the fabrication is presented in the “Experimental section/methods” section).

Meanwhile, the pronounced circular dichroism (CD) signal of the enantiomers indicates the formation of chiral structures (Fig. 1E). Then, the organic chiral semiconductor material with high mobility is used to fabricate a chiral device. The OFETs with asymmetric electrodes of gold and nickel are fabricated (Fig. 1F and G and Figs. S6 and S7), showing that spin-polarized carriers can be injected from the Ni electrode (Figs. S8 to S16). Through applying a cyclic magnetic field, the magnetic field dependence of chiral transport is studied. Under dark-state and photoexcitation conditions, the chiral signal exhibits 2 completely different line shapes (Fig. S17). A typical chiral signal under photoexcitation is shown in Fig. 1H; for R-type chiral devices, the strength of the CMC (*M–I*_SD_) is weaker under a positive magnetic field than under a negative magnetic field, while the opposite is true for S-type devices. It presents a multimode between the coupling between magnetic field and chiral field (Fig. 1H). However, in achiral FETs, there is no difference in the response of the magnetic field direction (Fig. S17). In the following, a systematic description and discussion will be carried out referring to the CMC signals under dark states and light excitation to comprehensively reveal their physical origin and potential application value.

### Characterization of the CMC signal in a dark environment

**Fig. 2.**
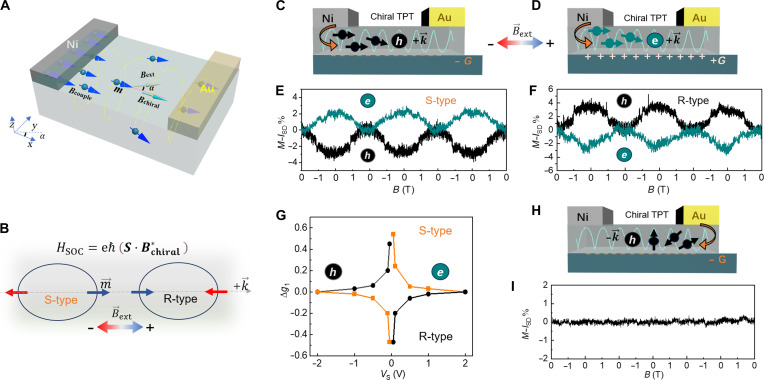
Dynamic evolution of the chiral magnetoconductance (CMC) signal in the dark environment. (A) The schematic diagram of the carrier transport process influenced by the equivalent magnetic field. (B) The schematic diagram of spin texture in chiral structure under hole transport, with a marked positive direction of the external magnetic field ${\vec{B}}_{\text{ext}}$ and the momentum $\vec{k}$. (C) The schematic diagram of hole-carrier transport ($+\vec{k}$) induced by negative gate voltage. (D) The schematic diagram of electron carrier transport ($+\vec{k}$) induced by positive gate voltage. (E) The CMC signal of S-type device at 300 K when carriers are injected from the Ni electrode with *V*_G_ = −5 V and *V*_SD_ = −0.005 V (hole) and *V*_G_ = 1 V and *V*_SD_ = 0.005 V (electron), respectively. (F) The CMC signal of R-type device at 300 K when carriers are injected from the Ni electrode with *V*_G_ = −5 V and *V*_SD_ = −0.005 V (hole) and *V*_G_ = 1 V and *V*_SD_ = 0.005 V (electron), respectively. (G) The $\Delta g_{1}\;$dependence on the source voltage *V*_SD_ for different chiral devices (R- and S-type) and different carriers (electron and hole) at 300 K. (H) The schematic diagram of the hole injection from the Au electrode. (I) The CMC signal of the S-type device when carriers are injected from the Au electrode with *V*_G_ = −5 V and *V*_SD_ = −0.005 V. All measurements were performed at 300 K.

In the dark environment in FET for hole-carrier transport at room temperature (Fig. S17A), the spin dynamics of a single carrier are affected by the total equivalent magnetic field resulting from multiple contributions, as shown in the schematic diagram (Fig. 2A). The effective magnetic field can be expressed as ${\vec{B}}_{\text{eff}}={\vec{B}}_{\text{int}}+{\vec{B}}_{\mathrm{ext}}+{\vec{B}}_{\text{couple}}+{\vec{B}}_{\text{chiral}}$, where ${\vec{B}}_{\mathrm{int}}$ is induced by the accumulation of spin polarization due to the ferromagnetic metal (FM) injection, ${\vec{B}}_{\text{ext}}$ is the external magnetic field, ${\vec{B}}_{\text{couple}}$ represents the existence of spin–spin interactions, and ${\vec{B}}_{\text{chiral}}\sim \left(\vec{p}\times {\vec{E}}_{\text{chiral}}\right)$ is the chiral potential field. A chiral crystal can be obtained with spin textures at the Fermi surface [29], where the spin orientation in the $\vec{k}$ direction is tagged (Fig. 2B). The spin polarization is collinear and locked to the momentum direction, which can be reinforced or disturbed by the equivalent magnetic field, resulting in a difference in the charge current along the collinear direction.

In the case of an S-type device, the current decreases (increases) throughout the negative (positive) magnetic field region (hole transport in Fig. 2E); this feature is plausible in the initial stage (from 0 to −1 T) because ${\vec{B}}_{\text{ext}}$ and $\vec{m}$ are antiparallel (Fig. 2B). However, the weakening of the external magnetic field did not change this decreasing trend (from −1 to 0 T); rather, a weak external magnetic field directly attenuates ${\vec{B}}_{\text{ext}}$, whereas the change in ${\vec{B}}_{\mathrm{int}}$ is limited owing to the small coercive field of the FM (Fig. S8). For ${\vec{B}}_{\text{chiral}}$, the continuous carrier injection makes a larger built-in electric field to further enhance ${\vec{B}}_{\text{chiral}}$. Therefore, the total equivalent magnetic field ${\vec{B}}_{\text{eff}}$ does not weaken drastically as the external magnetic field decreases. However, once the direction of the magnetic field changes, ${\vec{B}}_{\text{int}}$ and ${\vec{B}}_{\text{ext}}$ abruptly point to the opposite direction, and the ${\vec{B}}_{\text{couple}}$ becomes further disordered. Therefore, the total equivalent magnetic field acting on the carrier spin changes drastically, causing a turning point for the CMC signal at a zero magnetic field. It makes the CMC signals not coincident under the reciprocating sweep of the magnetic field (Fig. S17A). In addition, upon changing to an R-type chiral configuration, the opposite spin textures at the Fermi surface result in opposite CMC signals (Fig. 2B and F).

When the carriers change from holes to electrons, although the carriers are still injected from the Ni electrode and keep the positive momentum direction ($+\vec{k}$), the CMC curve is completely opposite (Fig. 2E and F). It is manifesting another important proof of spin–momentum locking mediated by spin–orbit coupling in chiral structures, as the electrons transported to the right are equivalent to the holes transported to the left. In addition, when changing α from 0° to 90°, i.e., the direction of spin polarization is perpendicular to the direction of momentum (Fig. 2B), the chiral signal disappears (Fig. S18). Moreover, the gate voltage perturbs the chiral potential field, resulting in further tuning of the chiral-related spin transport. A relative parameter $\Delta g_{1}\;$($\Delta g_{1}\;$= CMC_max_ − CMC_min_ in Fig. S17A) used to indicate the strength of the chiral-related spin. The increase in gate voltage leads to a rapid attenuation of the chiral-related spin signal, and this effect can also be achieved by increasing the source voltage (Fig. 2G and Figs. S19 to S39). Carrier injection from the Au electrode (Fig. 2H) does not result in the generation of any substantial signal (Fig. 2I and Figs. S19 to S39). This indicates that the synergistic effects of spin-polarized injection and chiral spin filtering are quite important factors for the observation of transmission over long distances in organic materials.

### Characterization of the CMC signal under illumination

**Fig. 3.**
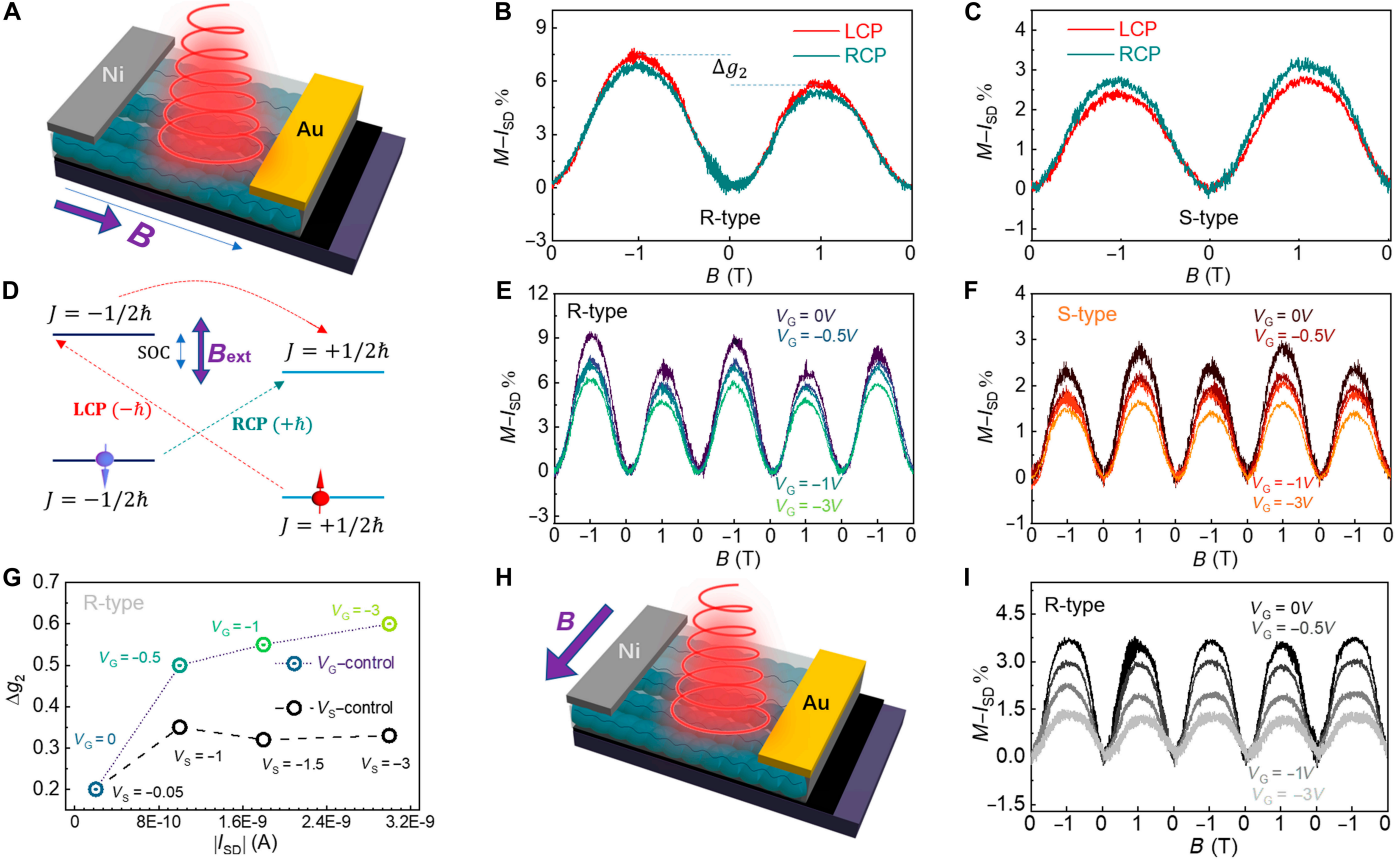
Dynamic evolution of the chiral magnetoconductance (CMC) signal under illumination. (A) The schematic diagram of CMC measurement under photoexcitation. (B) The CMC signal under left-handed circularly polarized light (LCP )and right-handed circularly polarized light (RCP) excitation for R-type device with *V*_G_ = −0.5 V and *V*_SD_ = −0.05 V at 300 K. (C) The CMC signal under LCP and RCP excitation for S-type device with *V*_G_ = −0.5 V and *V*_SD_ = −0.05 V at 300 K. (D) The schematic diagram of the spin selective transitions under LCP and RCP excitation in R-type device. (E) The CMC signal with the source voltage is fixed at −0.05 V, and the gate voltage changed from 0 to −3 V for the R-type device under LCP irradiation. (F) The CMC signal with the source voltage is fixed at −0.05 V, and the gate voltage changed from 0 to −3 V for the S-type device under LCP irradiation. (G) The $\Delta g_{2}\;$dependence on the source voltage *V*_SD_ and gate voltage *V*_G_ for R-type devices at 300 K. (H) The schematic diagram of CMC measurement with an external magnetic field vertical to the direction of carrier transport. (I) The CMC signal with the external magnetic field vertical to the direction of carrier transport. The source voltage is fixed at −0.05 V, and the gate voltage changed from 0 to −3 V for the R-type device under LCP irradiation.

The chiral FET exhibits a special response to circularly polarized light (CPL) at specific wavelengths, providing a new window to further investigate the effects of circular photon–CMC effects. As shown in Fig. 3A, under a magnetic field applied along the direction of carrier transport, the CMC curve obtained under light illumination differs substantially from that recorded under the dark environment. For R-type (or S-type) devices, the CMC signal generated by a negative magnetic field is substantially greater (weaker) than that of a positive magnetic field (Fig. 3B and C and Figs. S42 to S49). The external magnetic field can affect the electron transition under photoexcitation and thus increase the number of carriers, forming the basic MC (magnetoconductance) linear for both chiral and achiral devices (Fig. S17B). After that, the spin-polarized carriers are locked with momentum during transport in chiral potential field. Therefore, the CMC signal changes upon altering the direction of the external magnetic field. For R-type devices, the positive magnetic field is antiparallel to ${\vec{B}}_{\text{chiral}}$; this configuration is unfavorable for maintaining spin polarization when electrons are transported along the +*k* direction (Fig. 2B). Therefore, the CMC signal under a positive magnetic field is weaker than that under a negative magnetic field (Fig. 3B). Conversely, when using S-type devices, the CMC signal obtained under a positive magnetic field is stronger than that obtained under a negative magnetic field owing to the flipping of the spin texture (Fig. 3C). In addition, the signal intensity of the R-(S-) type device under left-handed circularly polarized light (LCP) light irritation is higher (weaker) than that obtained under right-handed circularly polarized light (RCP) light irradiation. Because of the conservation of angular momentum, the electrons with different spin orientations will undergo different relaxation processes with CPL excitation (Fig. 3D). Owing to the CMC signal, a chiral FET serves as an efficient platform for fabricating highly sensitive CPL detectors with a strong anti-interference ability. Figures S50 to S52 compare the photoexcited MC signal of the achiral-type device, which shows normal organic magnetic field effects, irrespective of the direction of the magnetic field.

Under CPL excitation, the gate could modify the chiral dependence of transport (Fig. 3E and F), especially to further increase the asymmetry factor (${\Delta g}_{2}$; defined in Fig. S17B) of the device (Fig. 3G). Traditionally, the gate voltage of FET will change in the number of injected carriers and the spin splitting energy level. However, by fixing the gate voltage, increasing the source voltage to increase the number of carriers, the chiral signal changes very weakly (Fig. 3G and Figs. S44 and S48). This result indicates that the effect of the gate voltage on ${\Delta g}_{2}$ is weakly dependent on the charge density. Thus, under the tunability of gate voltage of FET, the change of spin distribution and spin transitions caused by the rise and fall of the spin-polarized Ni/organic interface should be the main reason. In addition, upon changing the orientation of the magnetic field with respect to the direction of carrier transport, from parallel to vertical (Fig. 3H), the MC intensity recorded under the positive magnetic field remains the same as that obtained under the negative. In addition, the gate voltage loses its ability to regulate the chiral signal (Fig. 3I and Figs. S53 to S55).

### Comparison of the CMC signal between the dark state and the illumination

**Fig. 4.**
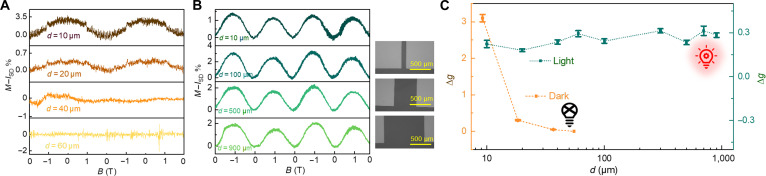
Comparison of the chiral magnetoconductance (CMC) signal between dark state and illumination. (A) The CMC signal under the dark environment for R-type device with different channel widths. The voltage parameters are *V*_G_ = −5 V and *V*_SD_ = −0.005 V, and the temperature is 300 K. (B) The CMC signal under the left-handed circularly polarized light (LCP) irradiation for R-type device with different channel widths. The voltage parameters are *V*_G_ = −3 V and *V*_SD_ = −0.05 V, and the temperature is 300 K. (C) Δgdependence on the channel width for R-type devices at 300 K temperature (left Δgand rightΔgrepresent the situation of dark and photoexcitation, respectively).

By changing the FET channel width, a clearer contrast in transport distance between dark environment and photoexcitation can be observed (Fig. 4). Under dark conditions, chiral-related transport occurs at tens of microns (Fig. 4A). However, with light excitation, chiral-dependent signals remain clear at distances over hundreds of micrometers. As shown in Fig. 4B and Fig. S56, OFETs with hundred-micrometer channels are prepared, and chiral-related signals persist in OFETs with a channel width of >900 μm (Fig. 4C and Fig. S56). The high-resolution transmission electron microscopy (HRTEM) of chiral nanofiber (Fig. 1D) shows a distinct fiber network and robust lattice structure in the thin film. The ordered lattice within fibers enhances chiral transmission, while the fiber network facilitates chiral-related transmission between fibers, enabling long-distance transport. The flexible adjustability of chiral-related spin implies that signals can be controlled by modulating variables such as channel width, gate voltage, and polarized light type.

In previous studies on chiral-related transport, the focus has mainly been on spin–orbit coupling caused by structural symmetry breaking, which, in turn, induces intrinsic transport in chiral structures. As an external chiral parameter, CPL has made rapid progress in areas such as chiral recognition and detection. However, there are currently limited reports on its role in chiral transport. Circularly polarized photons possess specific spin angular momentum, which can directly or indirectly affect the spin state of transporting electrons, laying the foundation for the specific transmission of chiral signals. Numerous chiral materials have shown excellent responses to polarized light, and, thus, devices fabricated using these materials open avenues for utilizing polarized photons in chiral signal transmission.

### Concept of multistate spin valves based on chiral materials

**Fig. 5.**
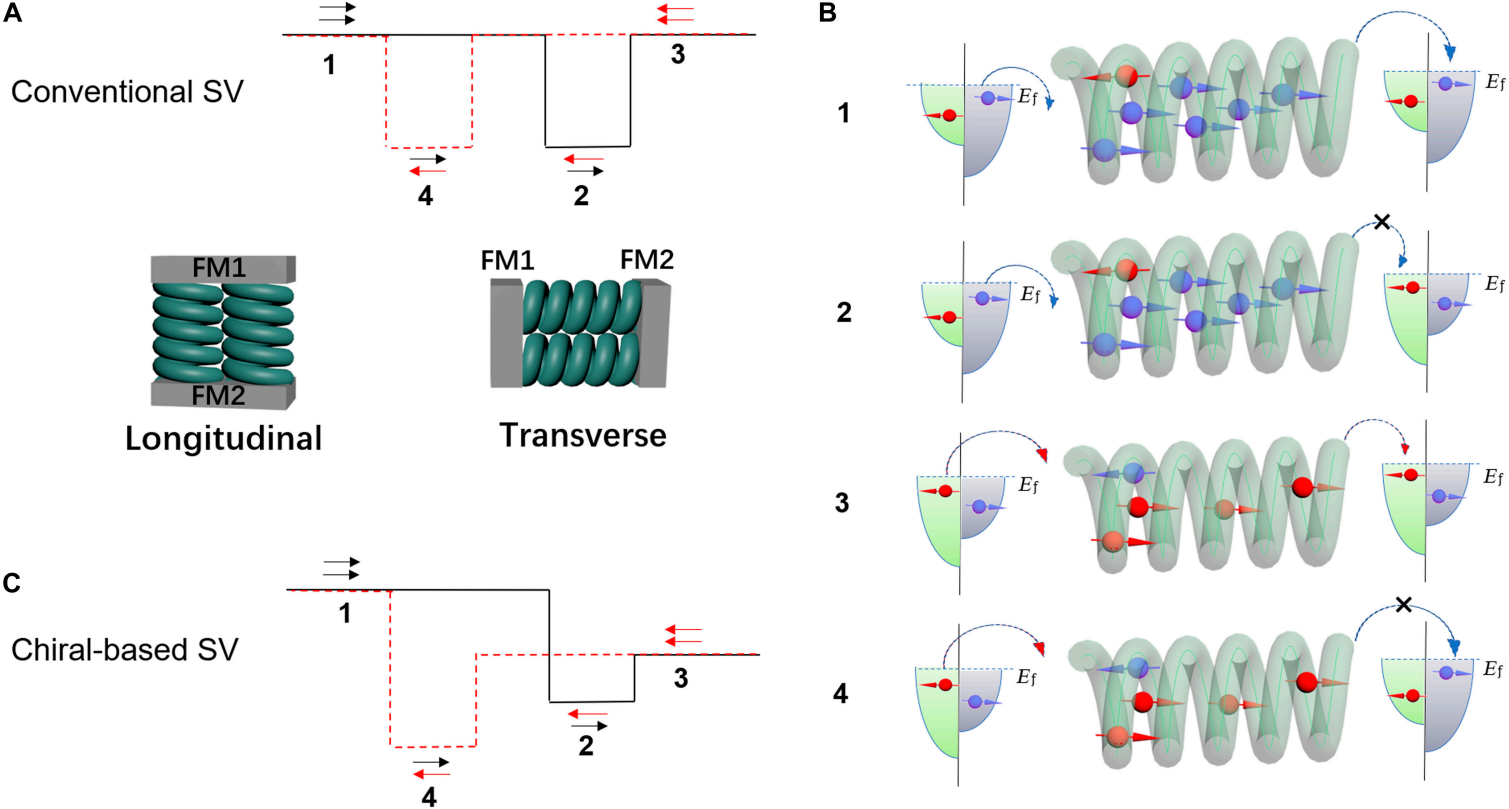
The concept of a multistate magnetoconductance (MC) device based on chiral spin characteristics. (A) The on–off MC states feature of a conventional spin valve (SV) based on ferromagnetic metal 1 (FM1)/normal medium/FM2 configuration. (B) The schematic diagram of different spin transport processes based on the cross of the chiral-induced spin selectivity (CISS) effect and the 2-circuit model. (1) FM1 and FM2 have the same magnetization, and CISS facilitates the spin transport. (2) FM1 and FM2 have different magnetization directions, and CISS facilitates the spin transport. (3) FM1 and FM2 have the same magnetization direction, and CISS restricts the spin transport. (4) FM1 and FM2 have different magnetization directions, and CISS restricts the spin transport. (C) The multiple MC states feature of a chiral-based spin valve based on FM1/chiral medium/FM2 configuration.

In principle, FET devices based on chiral structure can achieve the adjustment of chiral-related spin through the control of gate voltage, so as to ideally construct an MC device that is different from the traditional spin valves. However, even considering the weak spin–orbit coupling of organic materials and the potential long-distance maintenance of spin phase [37–40], it is still difficult to achieve such a function in a real sense. In previous work, relying on the vertical heterojunction of the FM–chiral medium–nonferromagnetic metal (NFM, the CISS effect with 2 MC states can be realized [33,34,41]. Furthermore, if the traditional spin valve effect relying on the 2-circuit model is combined with the CISS effect, it may be possible to construct a meaningful multistate MC device. As shown in Fig. 5, when the spin-polarized electrons are injected from the FM1, it will undergo CISS-related transport, exhibiting different MC signals even if the 2 different FM electrodes possess the same spin polarization. Correspondingly, if the 2 FM electrodes are oriented differently, it will also exhibit 2 configurations relying on the relative spin polarization direction. Therefore, unlike the traditional on–off configuration in spin valves with 2 MC states [42–44], the proposed chiral-based spin valve will exhibit 4 configurations. Given the current prevalence of the CISS effect at room temperature and the development of materials science, we believe that this type of chiral spin valve is achievable and has broad application potential.

## Discussion

In this study, an excellent organic semiconductor polymer, PCDTPT, was selected as a superior candidate material, conducted by tight-binding models and nonadiabatic evolution analysis, clarifying the optimal coupling size for carrier transport. Based on this, the targeted, specific chiral induction was carried out and further fabricated into an organic chiral FET device that demonstrates excellent chiral transmission characteristics. By changing the gate voltage to alter the type of carriers, the chiral-related spin of electrons and holes can be reversed in the same chiral-based device. In the dark environment, the transmission of the chiral-related signal can reach as long as 10 μm for both holes and electron transport. Besides, upon photoexcitation of organic chiral materials, another form of chiral CMC signal is obtained, which complements the situation in the dark environment due to the coexistence of spin-related transition and transport process. Leveraging the synergistic effects of spin-polarized injection and chiral spin filtering, the transmission distance of the chiral signal could be increased up to the order of millimeters, which represented the longest transmission distance achieved by these devices. Thus, the achieved long-distance chiral signal transmission, along with CPL-induced signal conversion and extension, are expected to promote the development of chiral spintronic platforms for both theoretical and practical applications. Finally, a valuable chiral spin valve was proposed to fulfill the requirements of multistate magnetoresistance for the development of chiral spintronics and related functional devices.

## Materials and Methods

### Experimental section/methods

#### Sample preparation

##### Preparation of chiral PCDTPT

PCDTPT was purchased from 1-material, 1,2-dichlorobenzene (1,2-DCB), acetonitrile (ACN), (R)-(+)-limonene, and (S)-(+)-limonene are purchased from Sigma-Aldrich Inc. All materials are used as received without further purification or treatment. First, PCDTPT (120 mg) is dissolved in 1,2-DCB (1 ml) at a concentration of 120 mg/ml; when the solution is heated in 45 °C, stirred, and then cooled to room temperature, (R)-(+)/(S)-(+)-limonene (2 ml) is added into the solution with a 2:1 volume ratio [(R)-(+) / (S)-(+)-limonene:1,2-DCB] to form a mixed solution of 40 mg/ml, respectively. The solution is stirred thoroughly to induce the formation of a chiral structure. Last, ACN (0.1 ml) is added at a 10:1 volume ratio of 1,2-DCB:ACN, and the solution is stirred until fully mixed. After a period of standing, the Chiral PCDTPT nanowire can be formed. In this process, different specifications of chiral polymer solutions can be obtained by adjusting the heating and stirring temperature, the proportion of inducers, the solution cooling time, etc. Meanwhile, frequent scanning electron microscopy (SEM) and HRTEM morphological characterizations are conducted to obtain the optimal structure. Dilution is performed by adding chlorobenzene to the prepared chiral PCDTPT solution, and a solution (5 mg/ml) was finally obtained for the preparation of FET devices. The PCDTPT films are prepared by coating the mixed solution on the glass to perform some characterization tests, including CD spectrum and SEM. To photograph HRTEM, the concentration of the chiral solution is diluted to 1 mg/ml. A reagent bottle containing deionized water is prepared in advance, 20 μl of chiral solution is extracted, and it is slowly added to the deionized water; a split interface will be formed between the chiral solution and the deionized aqueous solution. Then, a copper mesh is used to scoop up the chiral polymer from the supernatant solution. After this, the copper mesh is transferred to a nitrogen glove box for annealing at 100 °C for 10 min, removing as much solvent as possible from the polymer surface and further stabilizing the lattice structure. To obtain high-quality TEM images, numerous attempts are necessary.

#### Device preparation

Highly doped silicon is used and covered with silica as the substrate (orientation: <100>, thickness: 675 ± 25 μm, resistivity: <0.005 Ω·cm, 2,850 A ± 5% thermal oxide). The wafer is cut into a rectangular unit of 1 cm × 0.5 cm. The substrate is ultrasonically washed for 5 min using acetone, anhydrous ethanol, and deionized water, respectively. After that, the solvent is blown clean with nitrogen gas and irradiated with ultraviolet equipment to modify the silica surface, and the highly doped silicon is exposed as a gate by region etching.

A modified spin coater is used to fix the substrate, and considering the centrifugal force, it is recommended to place the sample in the symmetrical part of the circular spin turntable (10 cm in diameter). The chiral PCDTPT solution (5 mg/ml) was first centrifugally coated on the substrate at 5,000 rpm for 50 s and then annealed at 100 °C in a nitrogen atmosphere for 10 min. In addition, scraping can also be used to prepare ordered polymer films; however, the scraping parameters need to be adjusted in real time according to the molecular weight and concentration of the solution. For the solution concentration of 5 mg/ml used in this work, centrifugal spin coating may be more suitable, but for chiral solutions with higher concentrations or higher viscosity, scraping can also be a suitable option. It should be noted that to obtain the finely oriented chiral polymer film, a large number of attempts are necessary for both methods.

The prepared polymer film is fixed on a 2-dimensional material transfer table. The selected area is covered with a sheared polydimethylsiloxane (PDMS) membrane and then removed as a whole for the evaporation of the Au electrode. After this, the PDMS membrane is removed, the ruler is selected under the microscope, and the relevant area is covered with another PDMS membrane, ensuring that the 10-μm channel area is located in the overlapping area of the 2 times cover. Then, the evaporation of the Ni electrode is performed. The PDMS film is thick enough to ensure that the polymer surface in the covered area is not damaged by the thermal effects of electrode evaporation. The relevant schematics are included in the Supplementary Materials. For larger-scale channels, asymmetric electrodes can also be prepared with customized mask plates. After completing the first step of electrode evaporation, the sample is flipped to prepare the asymmetric electrode on the other side. However, this method will produce unstable errors due to operation and proficiency, but it also prepares asymmetric electrodes more conveniently. Regardless of the method used to prepare asymmetric electrodes, the same preparation parameters are used to obtain samples for SEM characterization before preparing the OFET device, which is beneficial for subsequent testing and observation of phenomena.

#### Measurements

TEM experiments were performed on a Cs-corrected FEI Titan transmission electron microscope at 300 kV. A direct-detection camera (Gatan K2) was used to acquire HRTEM images. A JASCO J-810 meter (Easton, MD, USA) with a response time of 1 s is used to characterize the CD spectra of the chiral PCDTPT films. A linear polarizer (Thorlabs) and a quarter-wave plate (Thorlabs) are used to obtain CPL in the 7-SCSpec system (Sofn Instruments Co. Ltd., China). The intensity of left-hand and right-hand CPL is calibrated by a standard Si detector (Newport 818-UV/DB). In the photocurrent tests, the polarized state of incident light is analyzed through a polarimeter (PAX1000, Thorlabs) combined with quarter-wave plates and linear polarizers. The 793-nm laser spot diameter of 2 mm can cover the working area of the OFET with different channels used in this work. The *J–V* curve of the device and MC test is characterized using a Keithley 4200 (Keithley Instruments Inc., Cleveland, OH, USA) with 2 output sources and a modular MOSFET test system and performed on the cryogenic magnetic field probe station. In different device testing processes, it is necessary to select voltage parameters reasonably, on the one hand, to control noise levels and current drift over time, and on the other hand, to control carrier concentrations to obtain comparable information.

#### Calculation details

To highlight the strong electron–lattice interactions in the 2 coupled molecular chains, an extended version of the tight-binding Su–Schrieffer–Heeger model is used. At the beginning of the dynamical simulations, we suppose that an electron has been injected into chain 1 in a localized state (i.e., a negative polaron $P^{-}$). Such a picture can be obtained by solving the Schrödinger equation of Hamiltonian *H* with ${\dot{u}}_{j,n}=0$. Based on this initial state, we further apply an electric field *E*(*t*) to drive its transport, where a nonadiabatic evolution method is employed to separately obtain the temporal evolution of the electronic state $\left|\Psi_{j,\mu }\left(t\right)\right\rangle$ and the lattice displacement $u_{j,n}\left(t\right)$ (i.e., lattice motion).

## Data Availability

The data that support the findings of this study are available from the corresponding authors upon reasonable request.
