# Supplementary material for: Switchable Ultralong Chiral Signal Transmission and Gate Tunability in Organic Chiral Semiconductor
Source: Research (Wash D C). 2026 Jun 15;9:1306. doi: 10.34133/research.1306 (PMC13266051; doi:10.34133/research.1306)
Supplement: Supplementary 1 — Figs. S1 to S58 [file research.1306.f1.docx]

Supplementary Materials

Switchable ultra-long chiral signal transmission and gate tunability in organic chiral semiconductor

Renjie Hu^1,2*^, Xiaoying Niu^1^, Xiangqian Lu^1^, Shilin Li^1^,Yuan Yu^1^, Xiangping Zhao^1^, Zhiwei Xiang^1^, Kepeng Song^3^, Ki Tae Nam^2^, Kun Gao^1^*, and Wei Qin^1^*

^1^School of Physics, State Key Laboratory of Crystal Materials, Shandong University, Jinan 250100, China

^2^Department of Materials Science and Engineering, Seoul National University, Seoul 08826, Republic of Korea

^3^School of Chemistry and Chemical Engineering, Shandong University, Jinan 250100, China

Email: [hurenjie744@gmail.com;](mailto:hurenjie744@gmail.com;) [gk@sdu.edu.cn;](mailto:gk@sdu.edu.cn;) [wqin@sdu.edu.cn](mailto:wqin@sdu.edu.cn)

**Contents**

**1** **The Tight-binding Model and Nonadiabatic Evolution Method**  **3**

**2 Preparation of samples and devices 10**

**3 Proof of spin-polarized electron injection and discussion of CISS 13**

**4 Basic characterization of the OFET devices 16**

**5 Dynamical chiral signal change of the OFET device with temperature and current intensity in the dark environment 19**

**6 Dynamical chiral signal change of the OFET device with gate regulation under laser illumination 42**

**7 The transmission limit of the chiral signal under laser illumination 56**

**8 Stability of chiral structures and devices 58**

**1. The Tight-binding Model and Nonadiabatic Evolution Method**


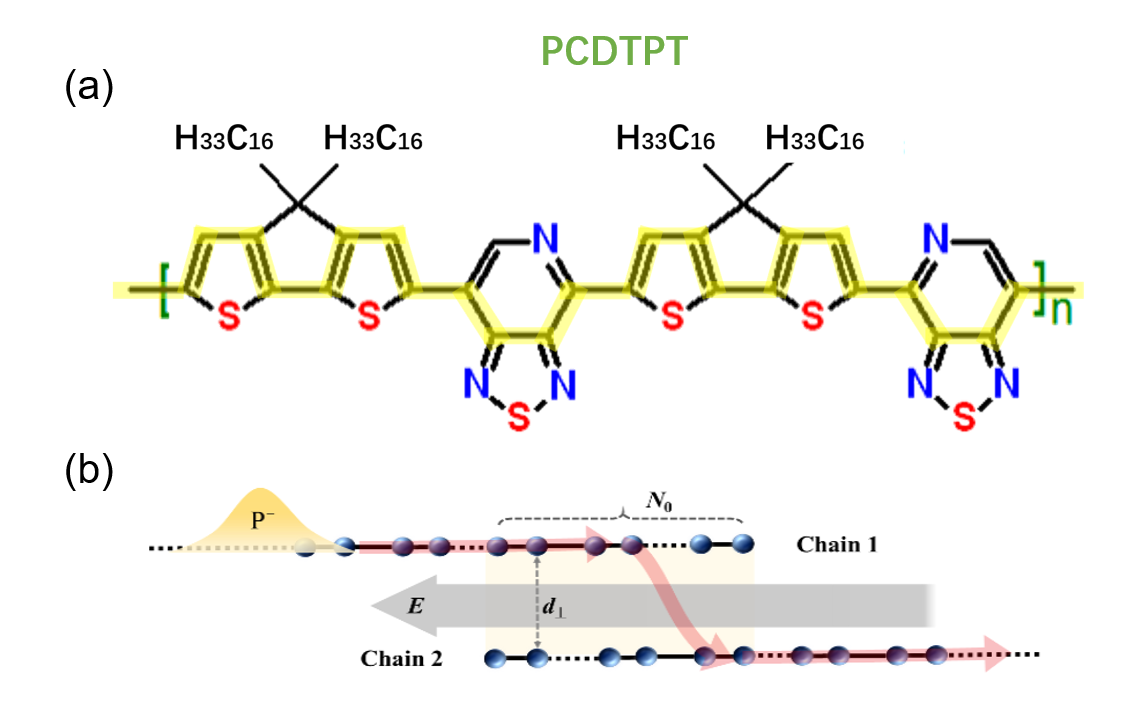


Figure S1: **(a)** Chemical structure of a PCDTPT molecule and the simplified model description along the conjugated carbon backbone, highlighted by yellow. **(b)** Schematic diagram of two simplified PCDTPT molecular chains with a parallel electric field *E* included, where the two chains are coupled in parallel and the size difference in the coupling region is emphasized.

We construct two parallel aligned PCDTPT molecules as the model system, where the size of their coupling region is variable. For simplicity, both of the two PCDTPT molecules are modeled as one-dimensional molecular chains (denoted as chain 1 and chain 2) along their conjugated carbon backbones, as sketched in Figure S1a. To highlight the strong electron-lattice interactions in such a system, an extended version of the well-known Su-Schrieffer-Heeger (SSH) model is employed^1^. The Hamiltonian of the two coupled molecular chains can be written as

$H=H_{1}+H_{2}+H_{\text{1-2}},$ (1)

where $H_{j}$ (*j*=1, 2 shows the chain index) describes an isolated molecular chain, consisting of two parts

$H_{j}=H_{j,e}+H_{j,lattice}.$ (2)

$H_{j,e}$ shows the electronic part,

$H_{j,e}=-\sum_{n} \left[ t_{0}-\alpha(u_{j,n+1}-u_{j,n})-(-1)^{n}t_{e} \right](C_{j,n+1}^{+}C_{j,n}+C_{j,n}^{+}C_{j,n+1}),$ (3)

$t_{0}$ is the hopping integral between the nearest sites for a uniform bond structure, $\alpha$ the electron-lattice interaction constant, $u_{j,n}$ the displacement of a unit at site *n*, $t_{e}$ the symmetry-breaking parameters introduced to reflect the lattice feature of the molecule, and $C_{j,n}^{+}$ ($C_{j,n}$) the creation (annihilation) operator of an electron at site *n*.

$H_{j,lattice}$ describes the classical treatment for the elastic potential energy and kinetic energy of a site, written as

$H_{j,lattice}=\frac{1}{2}K\sum_{n} (u_{j,n+1}-u_{j,n})^{2}+\frac{1}{2}M\sum_{n} \dot{u}_{j,n}^{2},$ (4)

*K* denotes the elastic constant, and *M* the mass of a site.

$H_{1-2}$ in Equation (1) describes the interactions between the two chains, expressed as

$H_{\text{1-2}}=-\sum_{m} t_{\perp}(C_{1,m}^{+}C_{2,m+N_{0}}+C_{2,m+N_{0}}^{+}C_{1,m}).$ (5)

$t_{\perp}=(t_{0}/10)\exp[1-2d_{\perp}]$ represents the interchain hopping integral between the nearest sites of the two chains, determined by the intermolecular distance $d_{\perp}$. *N*_0_ denotes the total site number in the coupling region. $\sum_{m}$ means the summation only for the sites over the coupling region between molecular chains.

At the beginning of the dynamical simulations, we suppose that an electron has been injected into chain 1 in a localized state (*i.e*., a negative polaron $P^{-}$), centered at site 40 [see Figure S1b]. Such a picture can be obtained by solving the Schrödinger equation of Hamiltonian *H* with $\dot{u}_{j,n}=0$. Based on this initial state of our model system, we further apply an electric field *E*(*t*) with the direction opposite to the molecular chains, as presented in Figure S1. Here, to mitigate the impact of a sudden application of the electric field on the system, *E*(*t*) is chosen as a semi-Gaussian form centered at time *t*_c_=75 fs with a width *t*_w_=25 fs,

$E(t)=\left\{ \begin{aligned} &-E_{0}\exp\left[ -(t-t_{c})^{2}/t_{w}^{2} \right],\quad t\leq t_{c}, \\ &-E_{0},\quad\quad\quad\quad\quad\quad\quad\quad t>t_{c}. \end{aligned} \right.$ (6)

*E*_0_ refers to the electric field strength. Thus, contribution of the electric field to the model system can be described as

$H_{E}=\left\{ \begin{aligned} &E(t)\sum_{n} e(na+u_{j,n})(C_{j,n}^{+}C_{j,n}-1),\quad\quad\quad\quad j=1, \\ &E(t)\sum_{n} e\left[ (n-N_{0})a+u_{j,n} \right](C_{j,n}^{+}C_{j,n}-1),\quad j=2. \end{aligned} \right.$ (7)

*e* indicates the electronic charge and *a* is the lattice constant. When the electric field *E*(*t*) is turned on, the initial negative polaron will experience an evolution, that is, driven to transport along the molecular chains, as described by the red arrowed curves in Figure S1b. Here, we employ a nonadiabatic evolution method similar to References 2 and 3, by which the temporal evolution of the electronic state $\left| \Psi_{j,\mu}(t) \right\rangle$ and the lattice displacement $u_{j,n}(t)$ (i.e., lattice motion) can be separately obtained. The lattice motion is classically described by the Newtonian equation of motion,

 (8)

in which the density matrix $\rho_{j,n,n^{'}}$ is defined as

$\rho_{j,n,n^{'}}=\sum_{\mu} \Psi_{j,\mu}(n,t)f_{\mu}\Psi_{j,\mu}^{*}(n^{'},t).$ (9)

$\Psi_{j,\mu}(n,t)=\left\langle n | \Psi_{j,\mu}(t) \right\rangle$ is the projection of electronic state $\left| \Psi_{j,\mu}(t) \right\rangle$ on the Wannier state of site *n*. $f_{\mu}$ is a time-independent distribution function, which is set as 0, 1, or 2, depending on the initial state occupation. Evolution of $\Psi_{j,\mu}(n,t)$ follows the time-dependent Schrödinger equation

 (10)

in which $j$ and $j^{'}$ mean different chains ($j^{'}=2$ in the case of $j=1$; and $j^{'}=1$ in the case of $j=2$). In the case of $j=1$, $n^{'}=n$; while in the case of $j=2$, $n^{'}=n-N_{0}$.

Equations (8) and (10) can be numerically solved by the Runge-Kutta method of order eight with step-size control. In all simulations, the total number of lattice sites for each chain is set as *N*=120. Referring to polyacetylene^4^, the values of model parameters are set as $t_{0}=2.5 \text{eV}$, $t_{e}=0.02 \text{eV}$, $K=2100 \text{eV/n}\text{m}^{2}$, $a=0.122 \text{nm}$, $M=1.35\times10^{5} \text{eV}\cdot\text{f}\text{s}^{2}\text{/n}\text{m}^{2}$, and $d_{\perp}=1 \text{nm}$.

**Results and Discussions**

By employing the tight-binding model and nonadiabatic evolution method, we simulate the transport dynamics of a negative polaron along the modeled molecular chains. Here, the size effect of their coupling region is focused on. At the initial time, we suppose that a negative polaron is formed in chain 1 with the center located at site 40 (far from the coupling region between chain 1 and chain 2), while chain 2 remains in the ground state. Upon application of an electric field, the negative polaron begins to transport along the chains. Through systematic simulations under a fixed intermolecular distance, we find that whether the negative polaron can scatter through the coupling region and continue to transport in chain 2 is closely related to the site number (*N*_0_) in the coupling region. As presented in Figure 1c, we compare the minimum critical electric field (*E*_c_), required for the negative polaron to traverse the coupling region and continue to transport under different values of *N*_0_. It is obvious that the strength of *E*_c_ first weakens and then strengthens with increasing the value of *N*_0_, indicating the existence of an optimal site number *N*_0c_ in the coupling region, which promotes the ease of interchain polaron transport. A further examination for the spatial size *r* of the coupling region [$r=(N_{\text{0c}}-1)a$, $a=0.122 \text{nm}$ indicates the lattice constant], corresponding to this optimal site number *N*_0c_, reveals its correlation with the spatial localization of the polaron. The inset in Figure 1c shows the lattice displacement order parameter $y_{n}=(-1)^{n}(2u_{n}-u_{n+1}-u_{n-1})$ of the polaron formed in chain 1, which presents an apparent spatial localization characteristic. Here, we introduce the polaron width *w*, defined as the spatial region spanned by $y_{n}$ varying from $-0.9y_{0}$ to $0.9y_{0}$ ($y_{0}$ represents the lattice displacement order parameter characterized by uniform dimerization). As indicated by the rectangular shaded area, the polaron width is about $w=30a$, which is comparable to the spatial size *r* of the optimal coupling region. In cases of $r<w$, it is difficult for the polaron to extend from chain 1 to chain 2 through the coupling region. Figure S2a demonstrates the polaron transport dynamics along the chains with *N*_0_ = 20 (i.e., $r=19a$). We can see that, after the polaron arrives at the right end of chain 1, it remains largely localized in chain 1, and a strong electric field is required to drive its extension into chain 2 for continued transport, as shown Figure S2b.


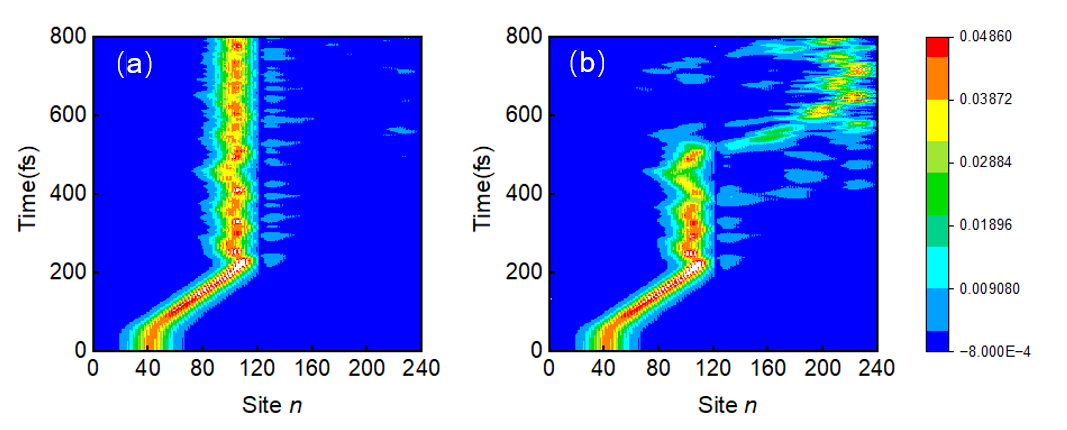


Figure S2. The polaron transport dynamics along the chains, where we show the evolution of the net charge density $q_{n}=e(\rho_{n,n}-1)$ ($\rho_{n,n}$ denotes the density matrix defined as Equation (9)). **(a)** and **(b)** shows the result with *N*_0_= 20 and the electric field separately chosen to be $E_{0}=15 \text{mV/nm}$ and $E_{0}=16 \text{mV/nm}$.

However, in cases of $r>w$ (e.g., $r=39a$), although the polaron can extend into chain 2, it tends to remain trapped within the coupling region^5, 6^, resulting in an oscillation behavior between chains, as presented in Figure S3c. It implies that a stronger electric field is required to drive the polaron out of the coupling region with a much larger size (Figure S3d). Only in the case of $r\approx w$, can the polaron not only extend readily from chain 1 to chain 2, but also escape efficiently from the coupling region, such that requires a weakest electric field to keep transport along the chains, as depicted in Figure S3a and S3b.


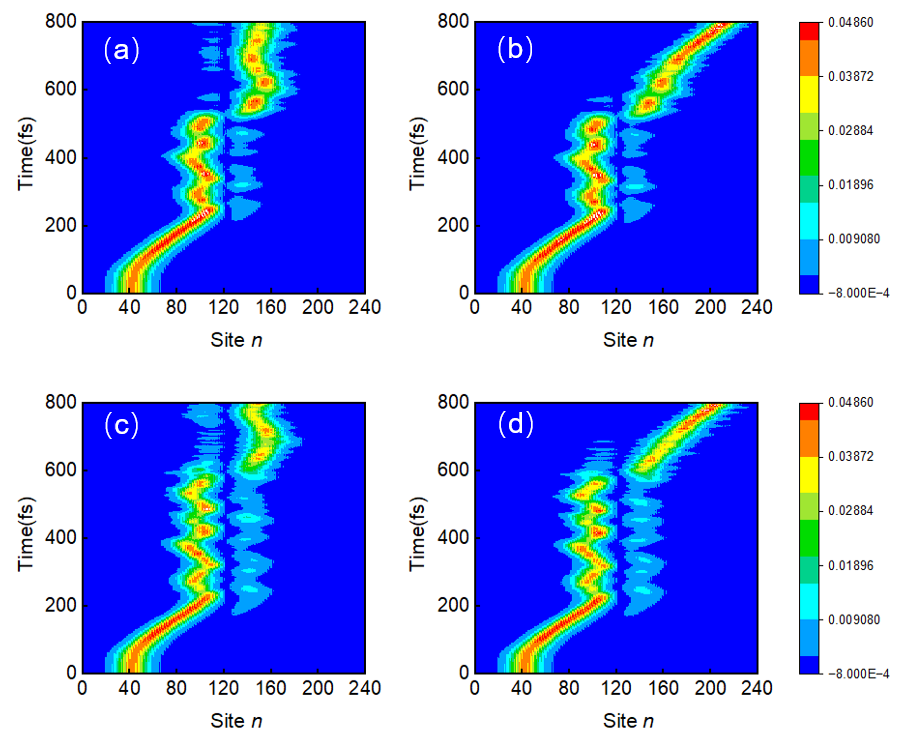


Figure S3. The polaron transport dynamics along the chains, where we show the evolution of the net charge density $q_{n}=e(\rho_{n,n}-1)$ ($\rho_{n,n}$ denotes the density matrix defined as Equation (9)). **(a)** and **(b)** shows the result with *N*_0_=30 and the electric field separately chosen to be $E_{0}=6 \text{mV/nm}$ and$E_{0}=7 \text{mV/nm}$; **(c)** and **(d)** shows the result with *N*_0_=40 and the electric field separately chosen to be $E_{0}=8 \text{mV/nm}$ and $E_{0}=9 \text{mV/nm}$.

Furthermore, we are attempting to gain a preliminary computational understanding of the role of photoexcitation in our work. By employing a tight-binding model, we first performed a static simulation of the polaron excited state in Figure S4**a**, and the results are shown in Figure S4**b.** Comparing the lattice displacement order parameter of the polaron and its excited state$y_{n}=(-1)^{n}(2u_{n}-u_{n+1}-u_{n-1})$, we can see that the delocalization of the polaron is significantly enhanced after excitation. This inevitably facilitates the intramolecular and intermolecular transport of the polaron, making it easier for the polaron to achieve directional transport under the drive of an external field.


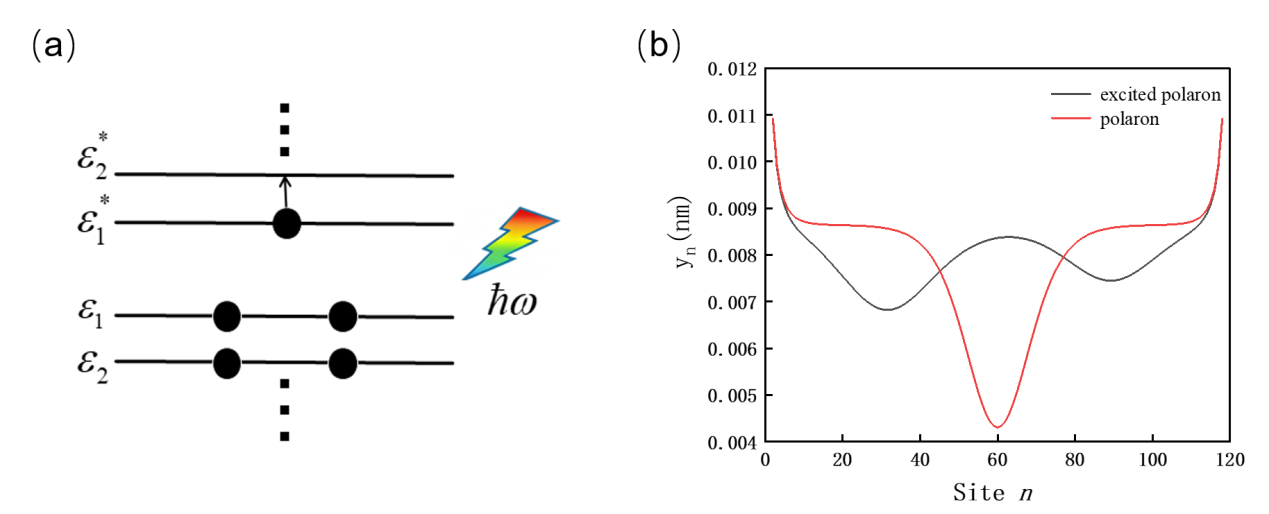


Figure S4. **(a)** Schematic diagram of energy levels excited by polarons.

Figure S4. **(b)** Lattice displacement order parameters of polarons and polaron excited states in a single chain.

To verify this idea, we applied a Gaussian femtosecond pulse electric field with a center of $t_{c1}$ and a width of $t_{w1}$to simulate the optical field, based on the original model, as follows:

$$E_{1}(t)=E_{1}\exp\left[ -(t-t_{\text{c1}})^{2}/t_{\text{w1}}^{2} \right]\cos(\omega t),$$

Where $E_{1}$ and $\omega$ describe the pulse electric field intensity and the photoexcitation frequency, respectively. Here, we first apply the optical field, taking $E_{1}=50 \text{mV/nm}$，$t_{c1}=\text{60} \text{fs}$，$t_{w1}=30 \text{fs}$，$\omega=0.1 \text{eV}$.

After the system stabilizes, we activate the driving electric field, which takes the following form:

$$E_{2}(t)=\left\{ \begin{aligned} &-E_{2}\exp\left[ -(t-t_{\text{c2}})^{2}/t_{\text{w2}}^{2} \right],\quad t\leq t_{\text{c2}}, \\ &-E_{2},\quad\quad\quad\quad\quad\quad\quad\quad t>t_{\text{c2}}. \end{aligned} \right.$$

Where $E_{2}$、$t_{\text{c2}}$、$t_{\text{w2}}$ represent the driving electric field strength, center, and width, respectively. Here, we take $t_{\text{c2}}=120 \text{fs}$，$t_{\text{w2}}=30 \text{fs}$, so the contribution of the electric field to the system can be described as:

$$H_{E}=\left\{ \begin{aligned} &(E_{1}(t)+E_{2}(t))\sum_{n} e(na+u_{j,n})(C_{j,n}^{+}C_{j,n}-1),\quad\quad\quad\quad j=1, \\ &(E_{1}(t)+E_{2}(t))\sum_{n} e\left[ (n-N_{0})a+u_{j,n} \right](C_{j,n}^{+}C_{j,n}-1),\quad j=2. \end{aligned} \right.$$

Where *e* is the electron charge, *a* represents the lattice constant, *j* is the molecular chain index, $u_{j,n}$ represents the displacement at lattice point *n*, and $N_{0}$epresents the total number of lattice points in the coupling region.

Preliminary results show that, in the original optimal coupling region $N_{0}=30$, the critical electric field Ec for polarons to achieve intermolecular transport decreased from 7 mV/nm to 4.5 mV/nm after applying a light field, as shown in Figure S5. This indicates that after applying a light field, the polarons' delocalization is enhanced due to excitation, and the electric field required for intermolecular transport becomes weaker. In the next step, we can build upon this foundation by selecting appropriate model parameters to conduct system simulations and study the complete picture of polaron transport in different molecular systems (including chiral molecules) under light field modulation.


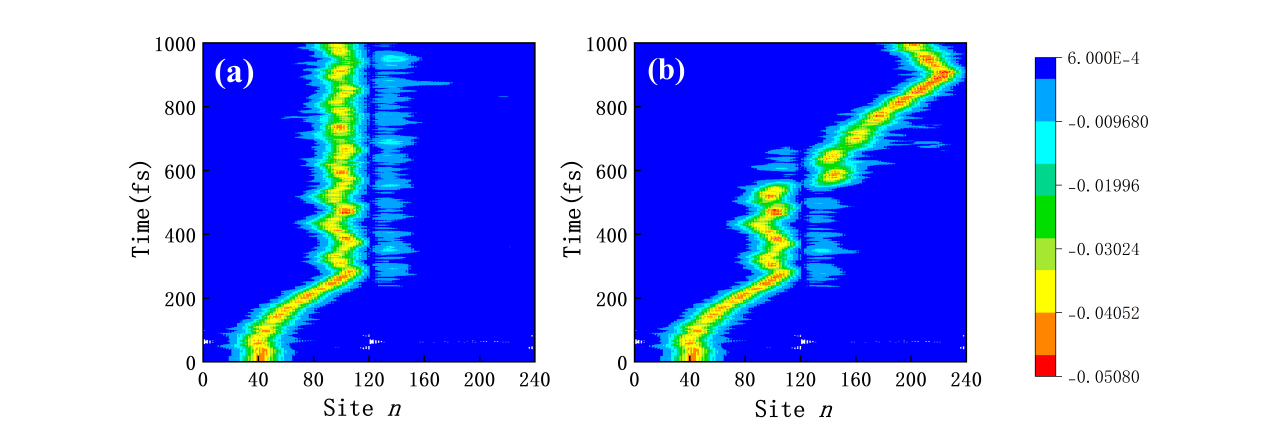


Figure S5. The polaron migration dynamics along the chains, where we show the evolution of the net charge density $q_{n}=e(1-\rho_{n,n})$ ($\rho_{n,n}$ denotes the density matrix defined as $\rho_{j,n,n^{'}}=\sum_{\mu} \Psi_{j,\mu}(n,t)f_{\mu}\Psi_{j,\mu}^{*}(n^{'},t)$). **(a)** and **(b)** shows the result after applying the optical field, with *N*_0_=30 and the electric field separately chosen to be $E_{2}=4 \text{mV/nm}$ and $E_{2}=4.5 \text{mV/nm}$.

**2 Preparation of samples and devices**


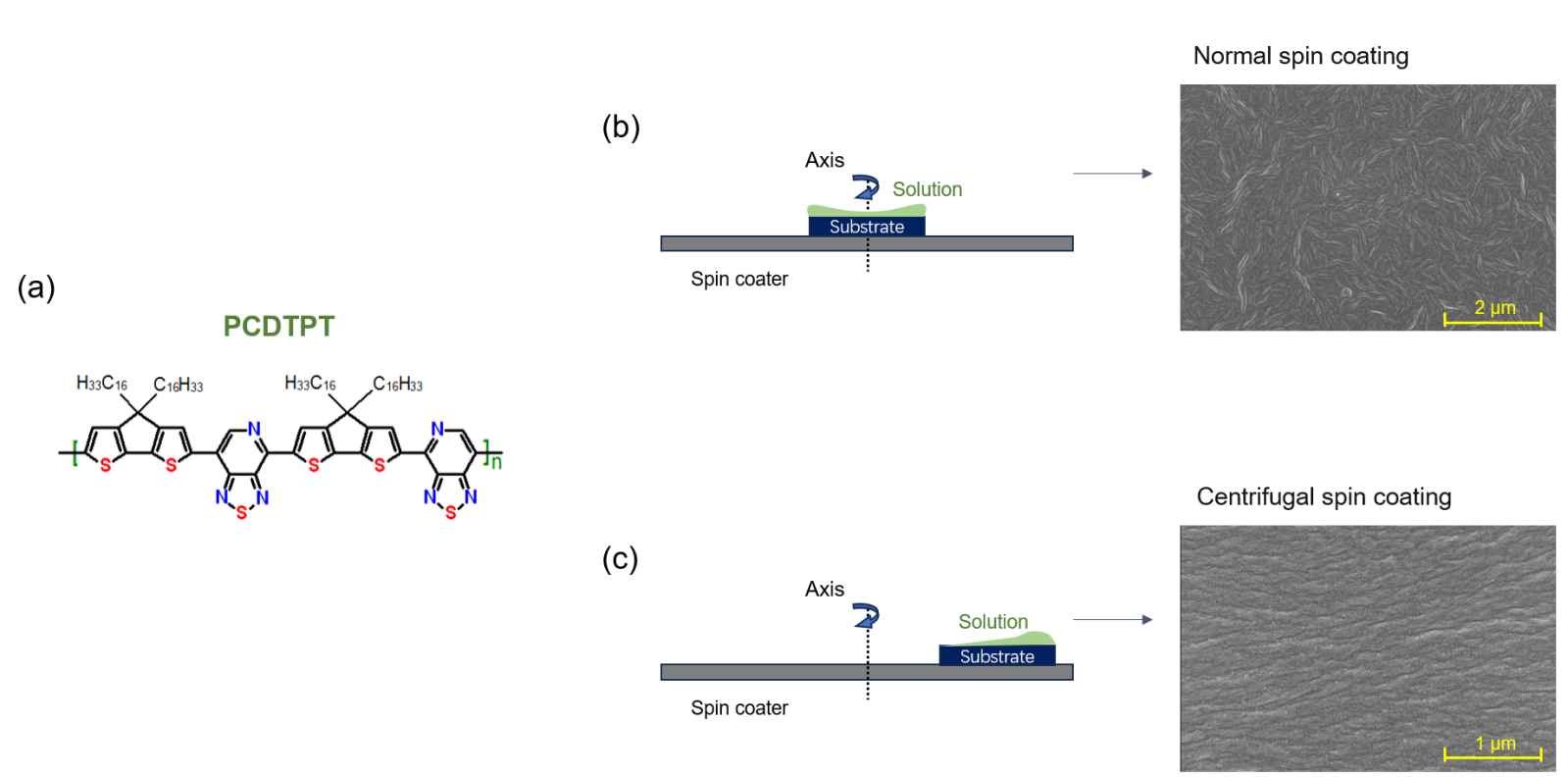


Figure S6: **The polymer** **structure and film sample preparation.** Normal spin coating (up figure) and centrifugal spin coating^7^ (down Figure), and corresponding SEM images.


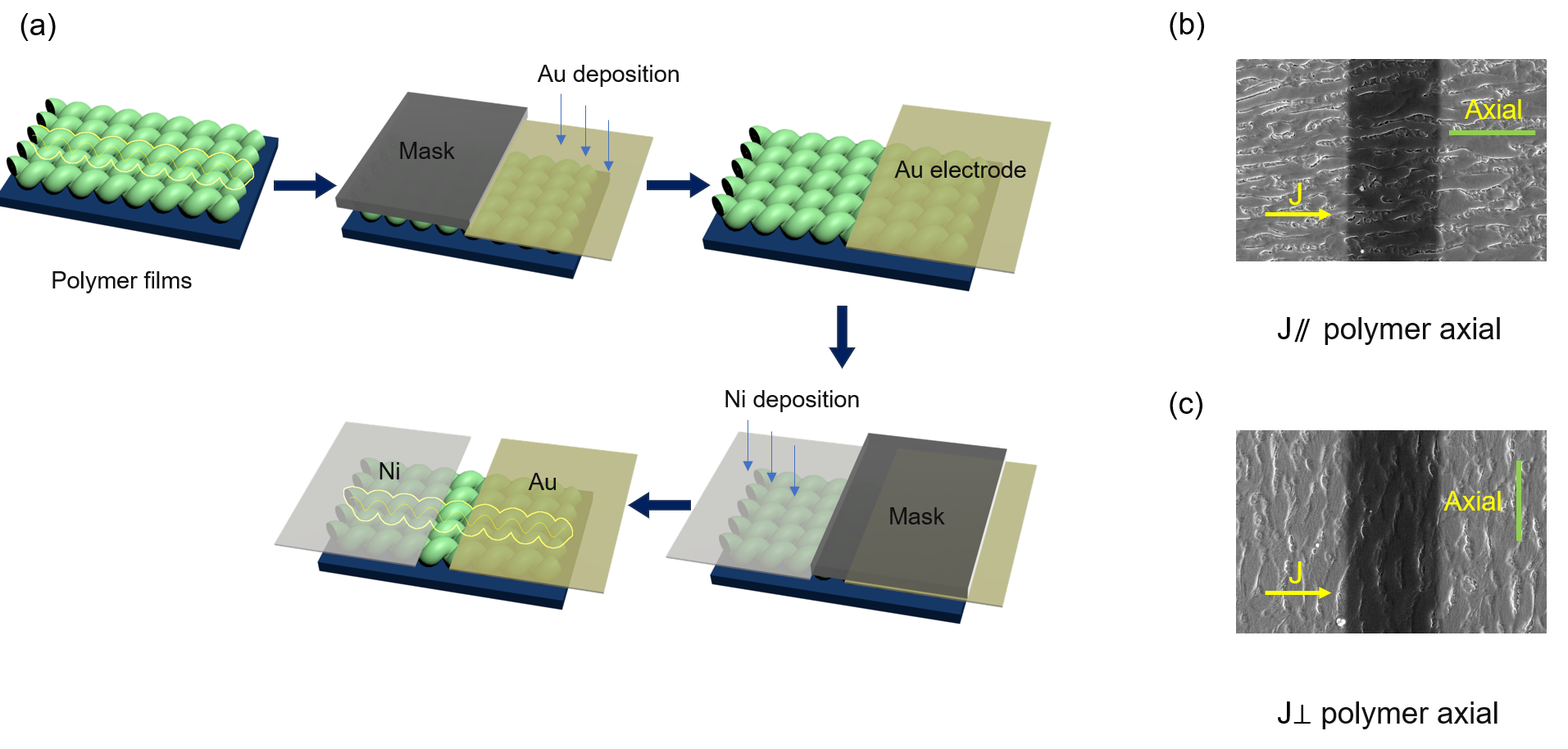


Figure S7: **Preparation of asymmetric electrodes (a)** The preparation process of asymmetrical electrode Au-Ni by the half-step mask method^8^, and the smallest channel is 10 μm. SEM of the polymer surface after the evaporation of the electrode. Two different configurations can be obtained: **(b)** the current direction is parallel to the polymer axial direction, and **(c)** the current direction is perpendicular to the polymer axial direction.


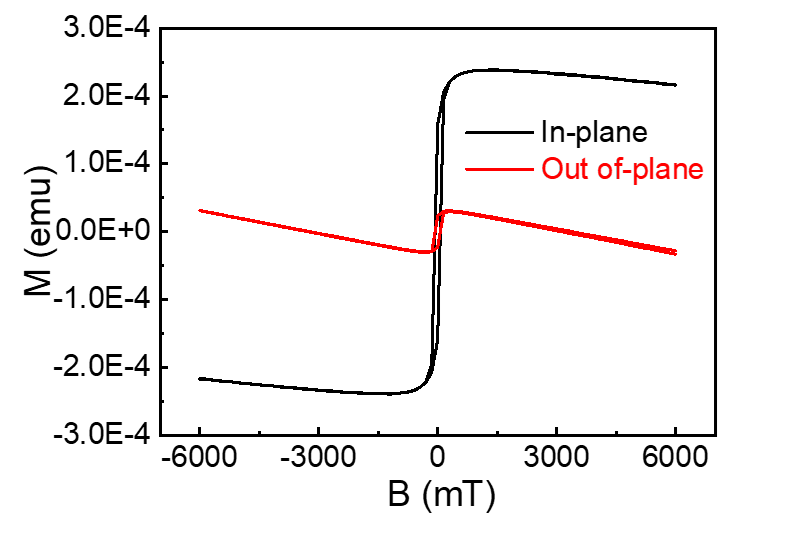


Figure S8: **The magnetization curve of the Ni electrode (30 nm)**. The characteristics of easy in-plane magnetization meet the requirement to inject in-plane spin polarization currents.

**3 Proof of spin-polarized electron injection and discussion of CISS**

In our work, the successful injection of spin-polarized electrons from ferromagnetic (FM) electrodes into chiral polymers is a prerequisite for subsequent studies, so it is necessary to confirm this fact. The combination of the sandwich structure of the asymmetrical electrode (typically FM/NFM) and the chiral material was used to verify the existence of the CISS effect in previous research^9, 10, 11^, and this can be the direct evidence that spin-polarized electrons are injected into the chiral layer from the magnetic electrode. Although the FET device and the sandwich device have different carrier transport modes, they share the same interface in terms of carrier injection (Figure S9). It is hoped that the CISS effect can be used to demonstrate the injection of spin-polarized electrons in our work.


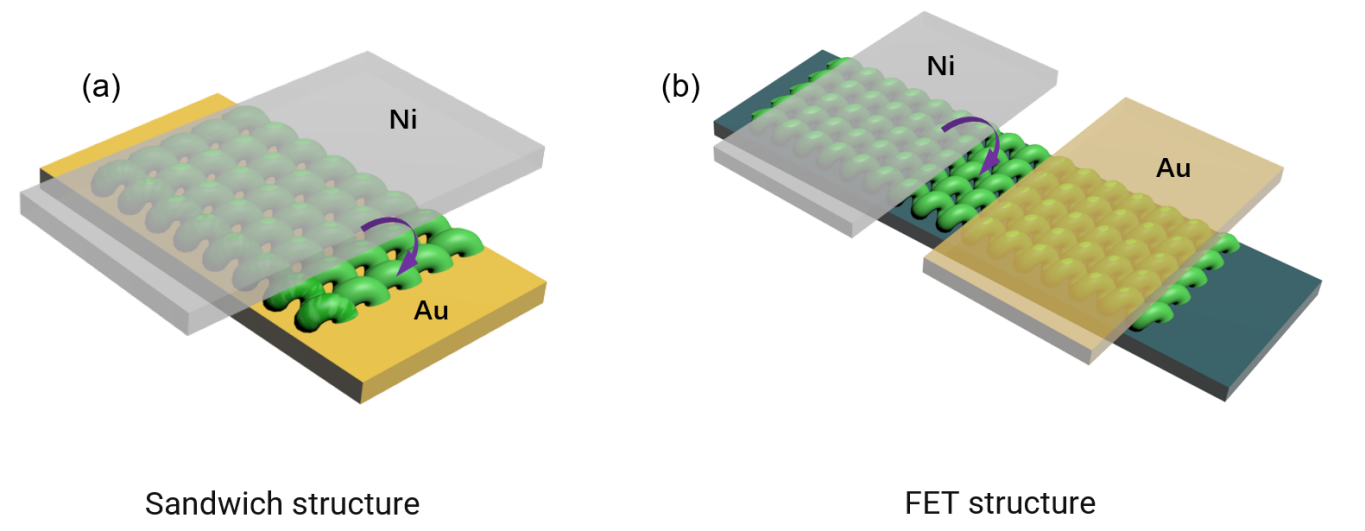


Figure S9: **Schematic diagram of different device structures.** The sandwich structure **(a)** with the carrier out-of-plane transport, and the FET structure **(b)** with the in-plane transport have the same spin injection interface.

First, we tested the J-V curve of the device, which exhibits nonlinearity and deteriorates as the temperature decreases (Figure S10), conforming to the carrier transport mode of the polymer material^12, 13^.


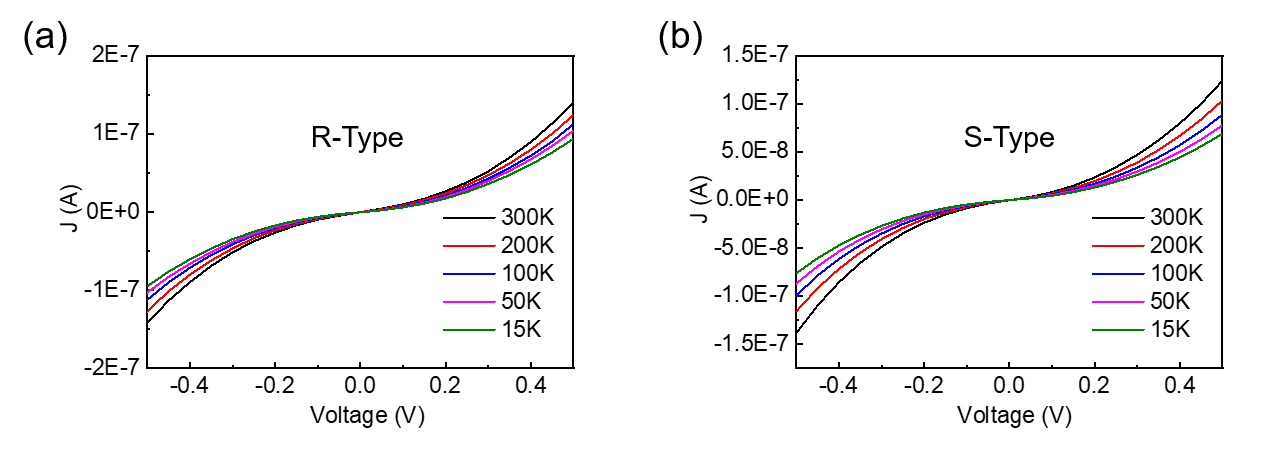


Figure S10: **J-V curves of sandwich devices with Ni-Au electrodes**. **(a)** R-Type device, **(b)** S-Type device.

Considering the orientation of the chiral axis, we have adopted three different magnetic field directions here to control the direction of spin polarization. As shown in Figure S11, the direction of the applied magnetic field is along the *x*, *y*, and *z* axes, respectively. Unfortunately, in either configuration, we do not find the occurrence of the CISS effect (Figure S11), that is, the magnitude of the current will change significantly with the direction of electrode magnetization. In the traditional CISS effect, when spin-polarized electrons flow through the chiral axis, electrons with different spin orientations will behave differently due to the presence of spin-orbit coupling and spin-momentum locking. Therefore, the direction of charge flow, the direction of the chiral axis, and the direction of spin polarization should be collinear with each other^11, 14, 15^. Based on this, the chiral axis along the z-direction was mostly used in early CISS studies^16, 17^, but recent studies have also shown that the characteristics of CISS can be detected even if the chiral axis is in the plane^18, 19^. Discussions on these issues are ongoing, but at least in our devices, the CISS effect cannot be used to demonstrate that the spin-polarized electrons are successfully injected from the Ni electrode into the chiral polymer.


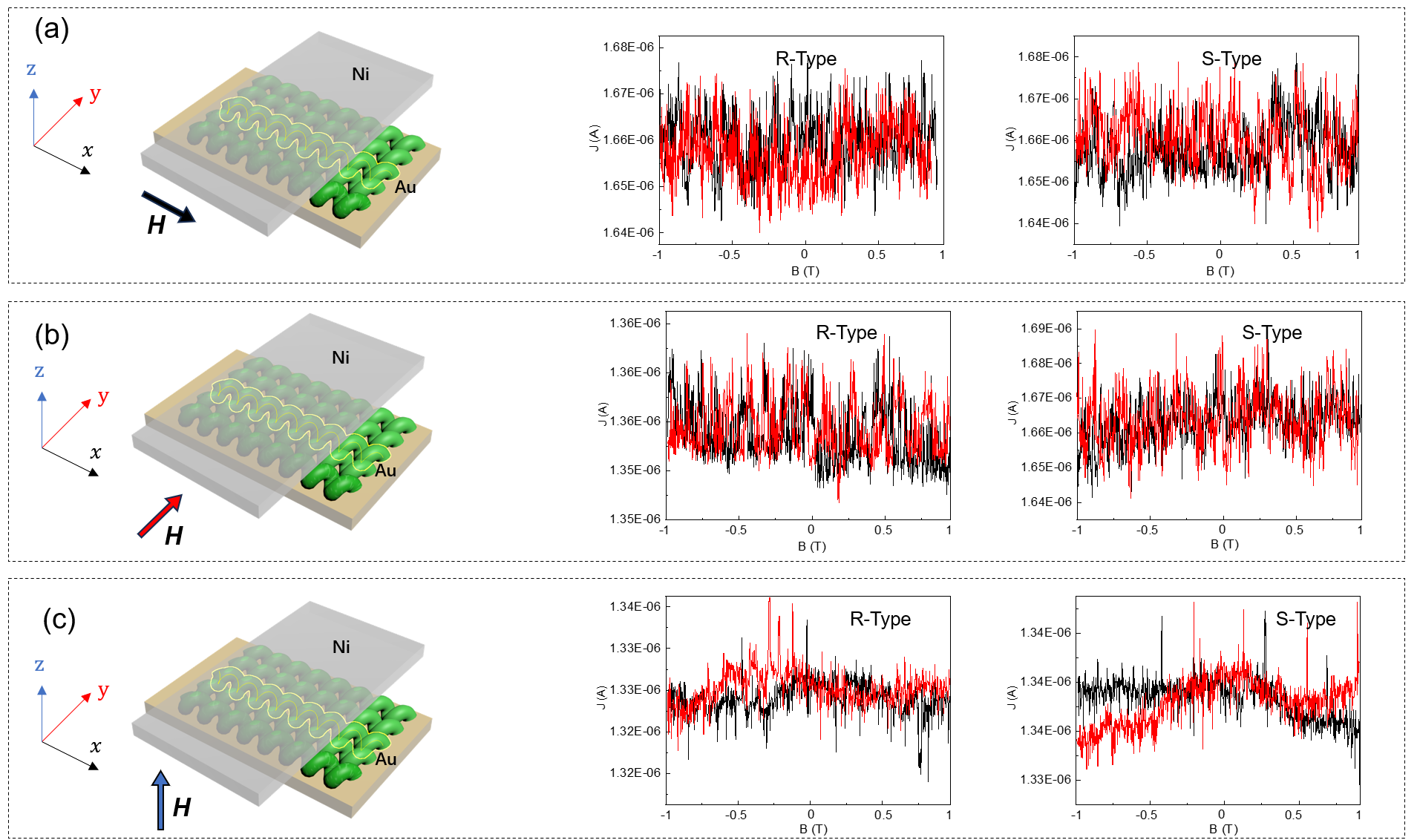


Figure S11: **Magnetic field effect sandwich devices with Ni-Au electrodes.** Left part: the schematic diagram of the device and relative magnetic field angles. Middle part: the current change of the R-type device with the magnetic field. Right part: the current change of the S-type device with the magnetic field. **(a)** The direction of the magnetic field is in-plane and parallel to the chiral axis. **(b)** The direction of the magnetic field is in-plane and perpendicular to the chiral axis. **(c)** The direction of the magnetic field is out-of-plane.

In addition to the use of asymmetric electrodes device FM/Chiral material/NFM, which are commonly used in CISS studies, we further used the typical spin valve structure FM1/ Chiral material/FM2, which is commonly used in spintronics^20^ (Figure S12), to verify the injection of spin-polarized electrons.


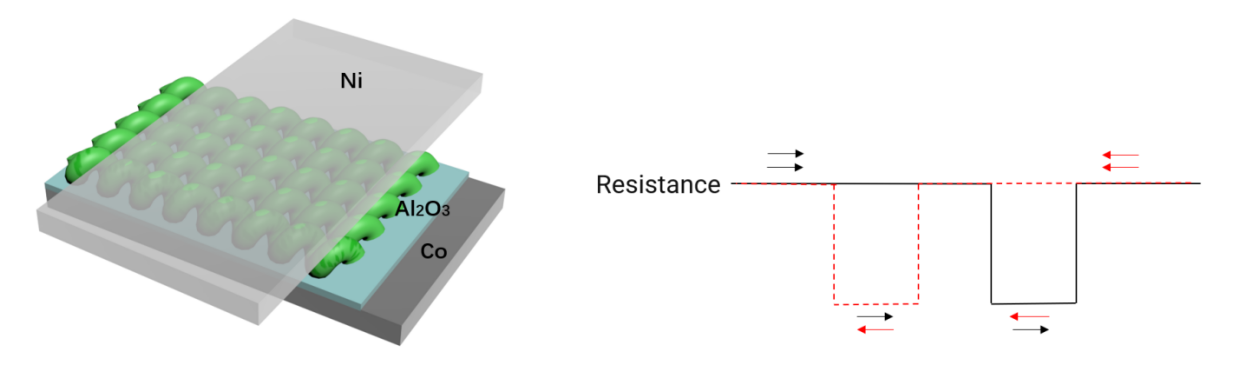


Figure S12: **Schematic diagram of the device using a conventional spin valve structure.** The red and black arrows in the right part represent the relative magnetization orientation of the nickel and cobalt electrodes, respectively.

First, we still verified the J-V curve of the device (Figure S13), which is consistent with the basic characteristics of carrier transport in polymers.


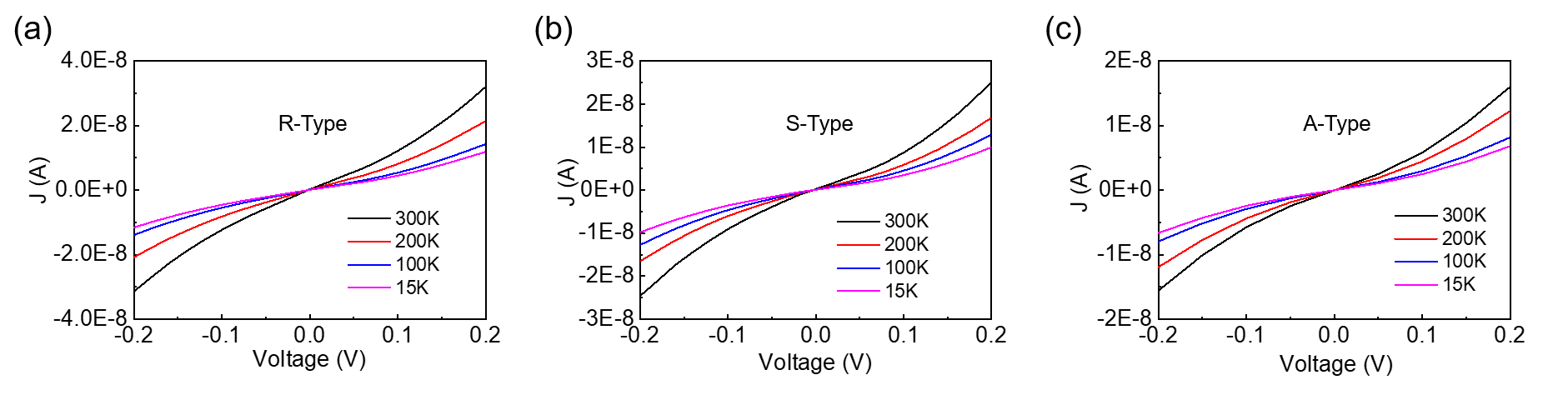


Figure S13: **The J-V curves of spin valve devices.** **(a)** R-Type, **(b)** S-Type, and **(c)** Achiral-Type.

Through the cyclic magnetic field sweep, the normal spin valve characteristic curve can be obtained (Figure S14), that is, when the Ni electrode and the Co electrode are counterparallel magnetized, the device has a bigger resistance, which is a robust proof that the spin polarized current can be well injected into the polymer through the Ni electrode, even if there is no additional transport layer between the Ni electrode and the chiral polymer.


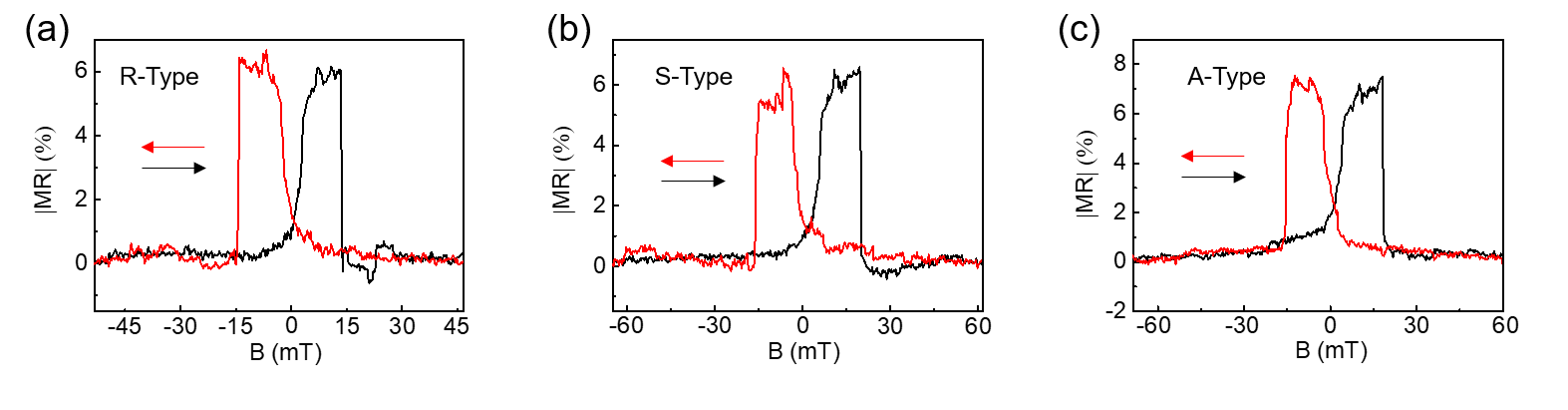


Figure S14: **The characteristic curve of the spin valve device.** Black and red arrows represent the direction of the magnetic field sweep. **(a)** R-Type, **(b)** S-Type, and **(c)** Achiral-Type. In all devices, the operating current is controlled at around 10^-6^ A.

In summary, although we have used the common asymmetric electrode (Ni-Au) sandwich devices, the CISS effect has not been observed, and the specific mechanism is still contradictory, but through the traditional spin valve structure (Ni-Co), we can still prove the successful injection of spin-polarized electrons, which lays a solid foundation for subsequent research.

**4 Basic characterization of the OFET devices**


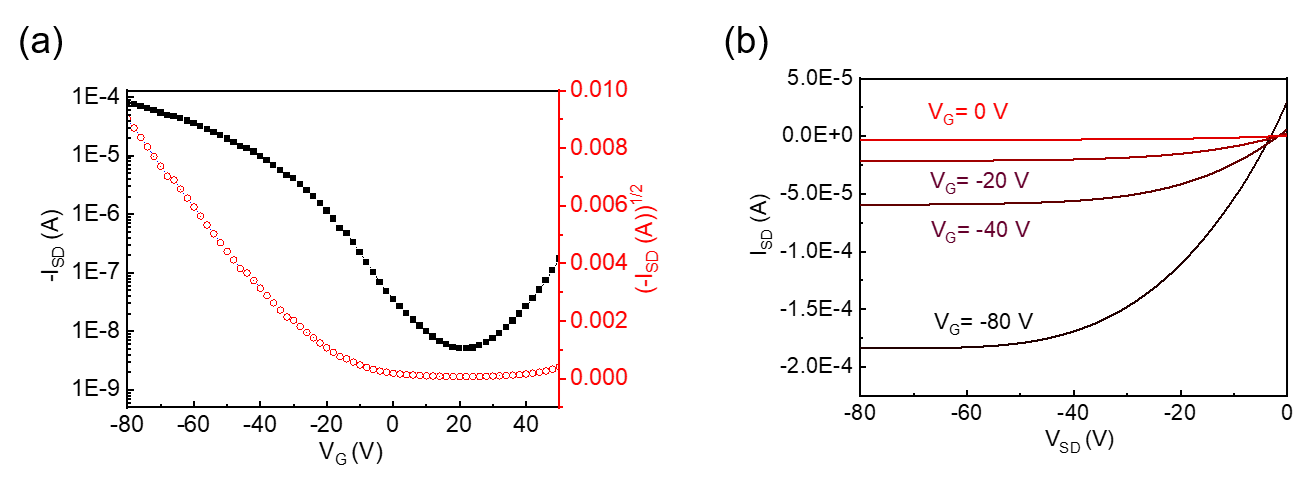


Figure S15: **Basic characterization of the OFET devices (R-Type) with 10 μm channel. (a)** Curves of FET transfer characteristics at 300 K, with V_SD_= -60 V. **(b)** Transport characteristic curves at 300 K.


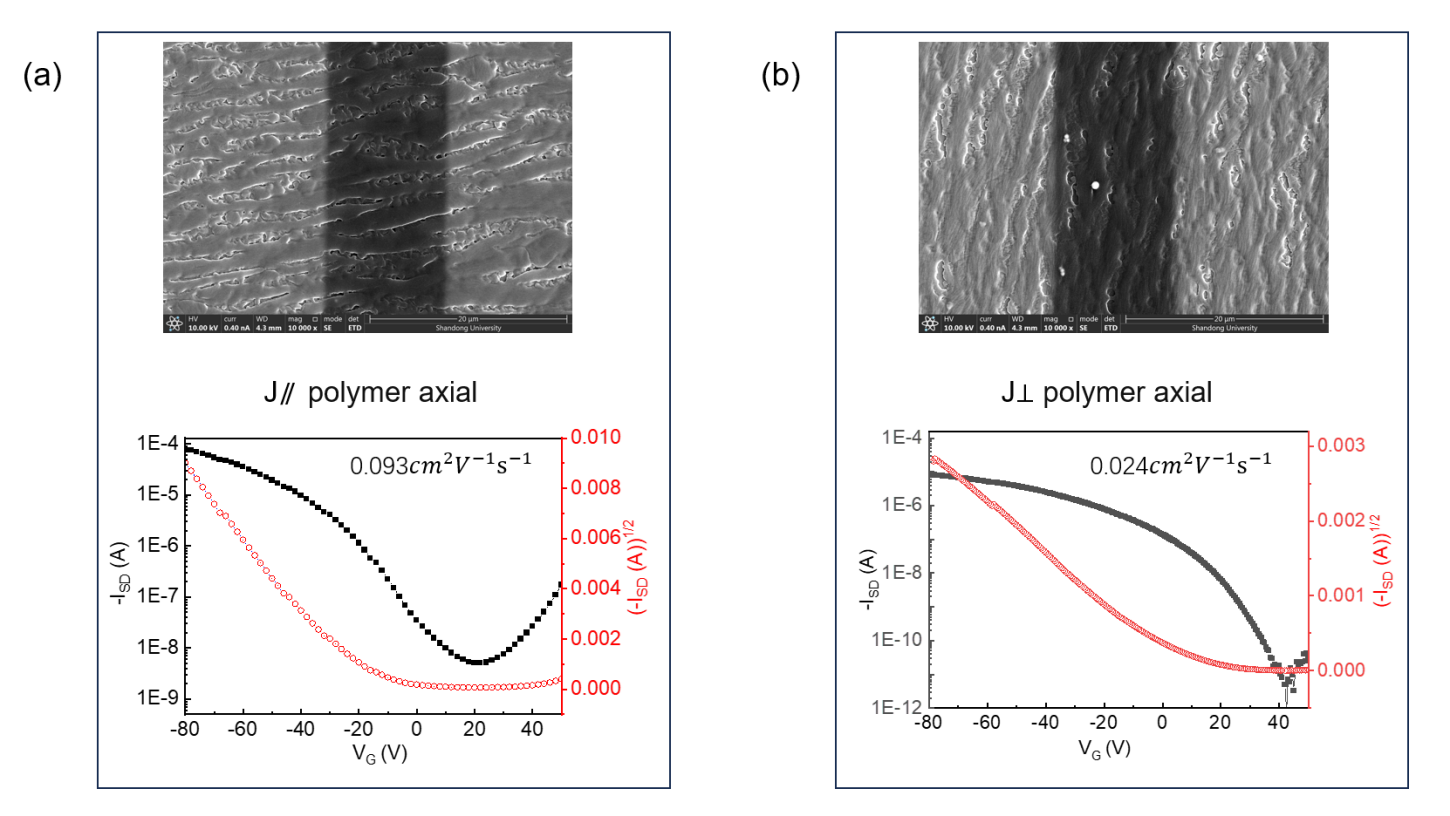


Figure S16: **Transfer characteristic curves of devices with different configurations (R-Type). (a)** Transfer characteristic curve in configuration J *∥* polymer axial, the mobility is 0.093$cm^{2}V^{-1}s^{-1}$. **(b)** Transfer characteristic curve in configuration J ⊥ polymer axial, the mobility is 0.024$cm^{2}V^{-1}s^{-1}$.

As shown in Figure S17a, for the chiral R-type device in a dark environment (hole transport), the CMC signal increases over the entire negative magnetic field region, regardless of whether the magnetic field is increasing or decreasing. When the direction of the magnetic field switches from negative to positive, the CMC signal weakens. In contrast, the S-type device presents an opposite trend and has a relatively minor effect compared to the R-type device. However, for an achiral-type device, the curve of the MC is consistent with the applied magnetic field, indicating no correlation with the field direction. By extracting the CMC signal under one complete magnetic field period, we noticed that the CMC signals of the R-type (S-type) devices could not be superpositioned under the reciprocating sweeps of positive and negative magnetic fields, showing a clockwise (counterclockwise) shape (right part of Figure S17a). This phenomenon is completely different from the CISS effect, where the signal shape is consistent with the magnetization curve of the magnetic electrode. Interestingly, under laser illumination, CMC signals (Figure S17b) present a pronounced difference from those in the dark (Figure S17a). For R-type devices, the strength of the CMC is weaker under a positive magnetic field than under a negative magnetic field, while the opposite is true for S-type devices. However, in achiral FETs, there is no difference in the response of the magnetic field direction. Therefore, the evolution of spins should undergo a more complex dynamics in chiral FET with different scenario.


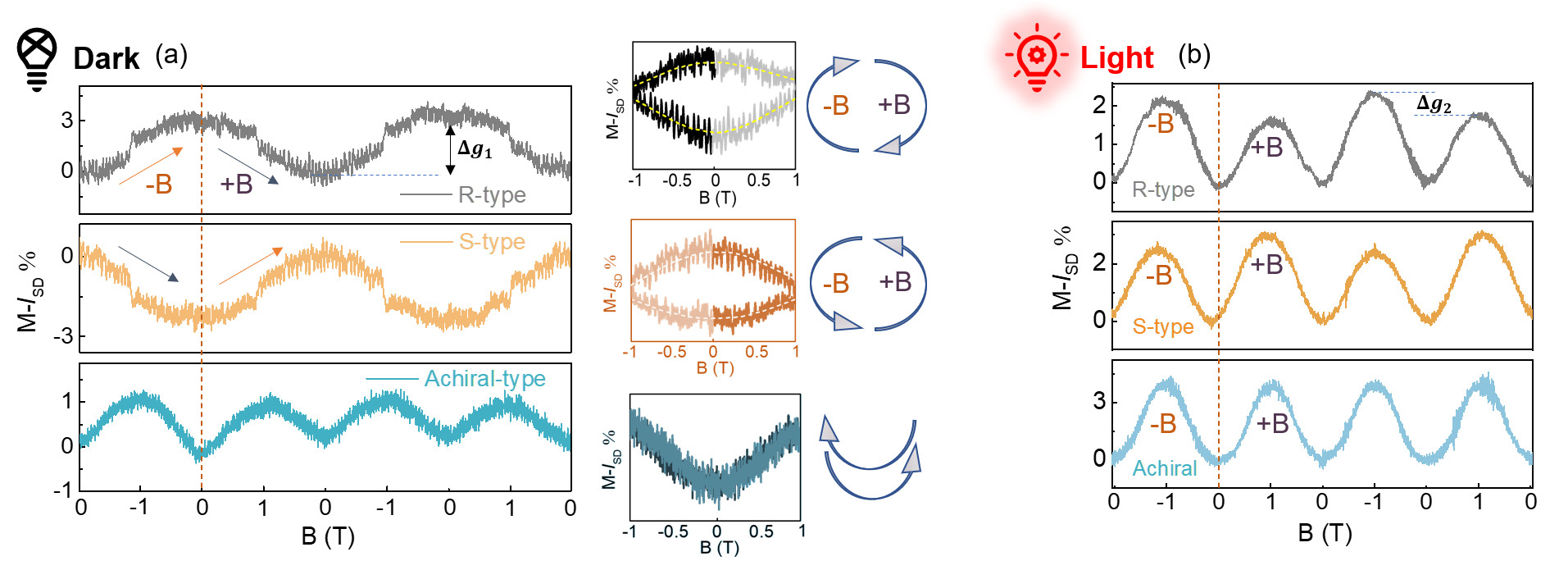


Figure S17: **(a)** The CMC signal (hole transport) under multiple cycles of external magnetic fields for R/S/Achiral-type devices in the dark environment (left part). Here, $\Delta g_{1}$ is the maximum change of CMC ($\Delta g_{1}$=CMC_max_-CMC_min_). In the right part, the CMC signal is extracted over a full period. V_G_= -5 V, V_SD_= -0.005 V in all the three devices. **(b)** The CMC signal under multiple cycles of external magnetic fields for R/S/Achiral-type devices under photoexcitation, the laser wavelength is 793 nm. V_G_= -3 V, V_SD_= -0.05 V in all the three devices, $\Delta g_{2}$=2(CMC_B=-1T_ -CMC_B=+1T_)/ (CMC_B=-1T_ +CMC_B=+1T_). All measurements were performed at 300 K.


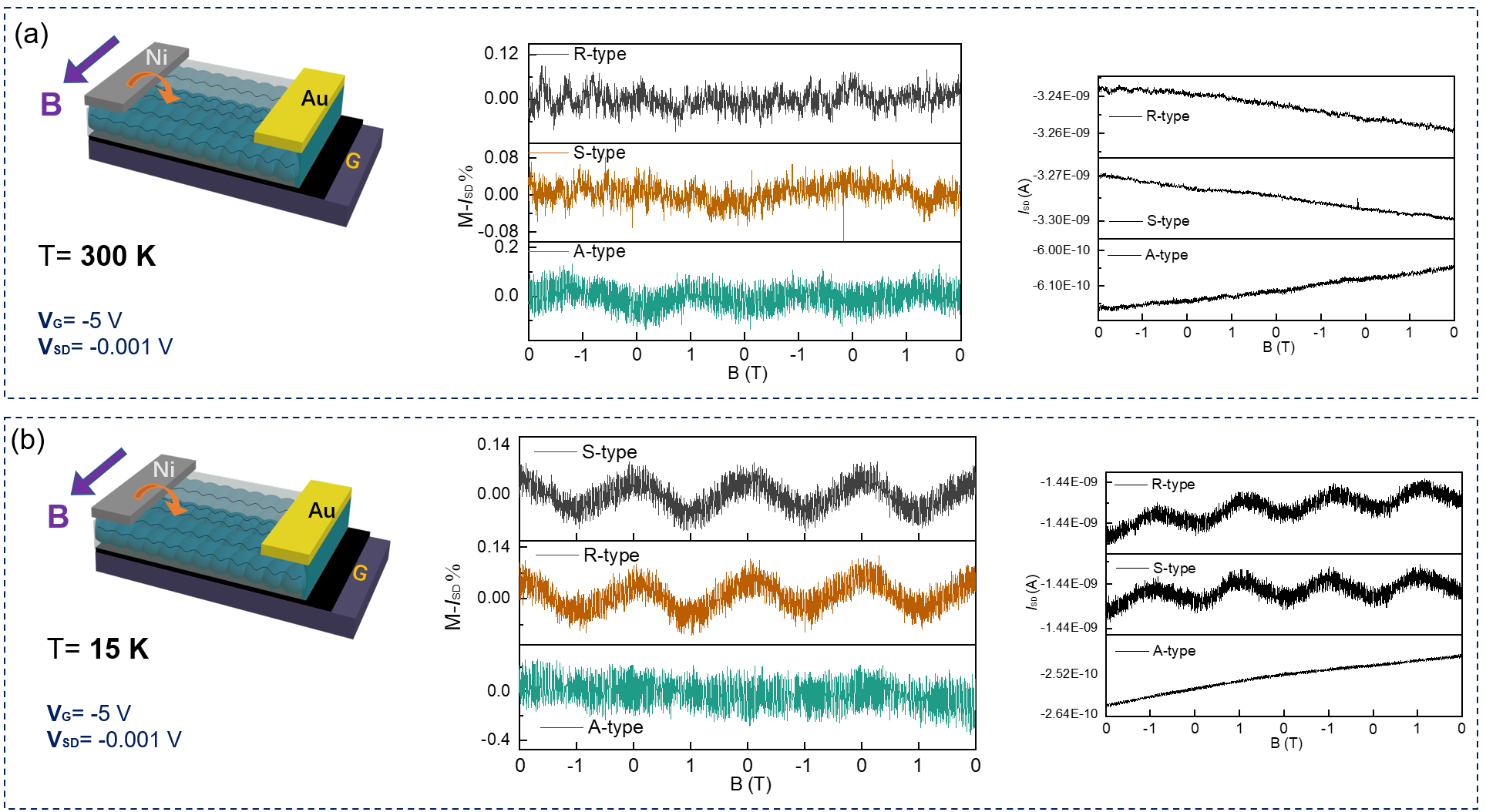


Figure S18: **(a)** Left part: Schematic diagram of hole transport in a FET device, the direction of the magnetic field is in plane and perpendicular to the direction of carrier transport. Middle part: The percentage change in the current of the FET with different chirality as a function of the magnetic field. Right part: The original curve of the current as a function of the magnetic field. The temperature is 300 K. **(b)** Left part: Schematic diagram of hole transport in a FET device, the direction of the magnetic field is perpendicular to the direction of carrier transport. Middle part: The percentage change in the current of the FET with different chirality as a function of the magnetic field. Right part: The original curve of the current as a function of the magnetic field. The temperature is 15 K.

**5 Dynamical chiral signal change of the OFET device with temperature and current intensity in the dark environment**

From Figures S19 to S41, a series of CMC signal changes dynamically with current intensity and temperature in a dark environment are shown (20 μm channel). For clarity, here is a brief explanation.

Figures S19-22: **S-type** device, carriers are injected from the **Ni** electrode.

Figures S25-29: **S-type** device, carriers are injected from the **Au** electrode.

Figures S30-34: **R-type** device, carriers are injected from the **Ni** electrode.

Figures S35-39: **R-type** device, carriers are injected from the **Au** electrode.

Figures S40-41: **A-type** device.

In most cases, the Figure is configured as follows. Left part: Schematic diagram of carrier injection and transport. Middle part: The percentage change in the current of the FET with the magnetic field. Right part: The raw curve of the current changes with the magnetic field.

**Figure S17-20: S-type device, carriers are injected from the Ni electrode.**


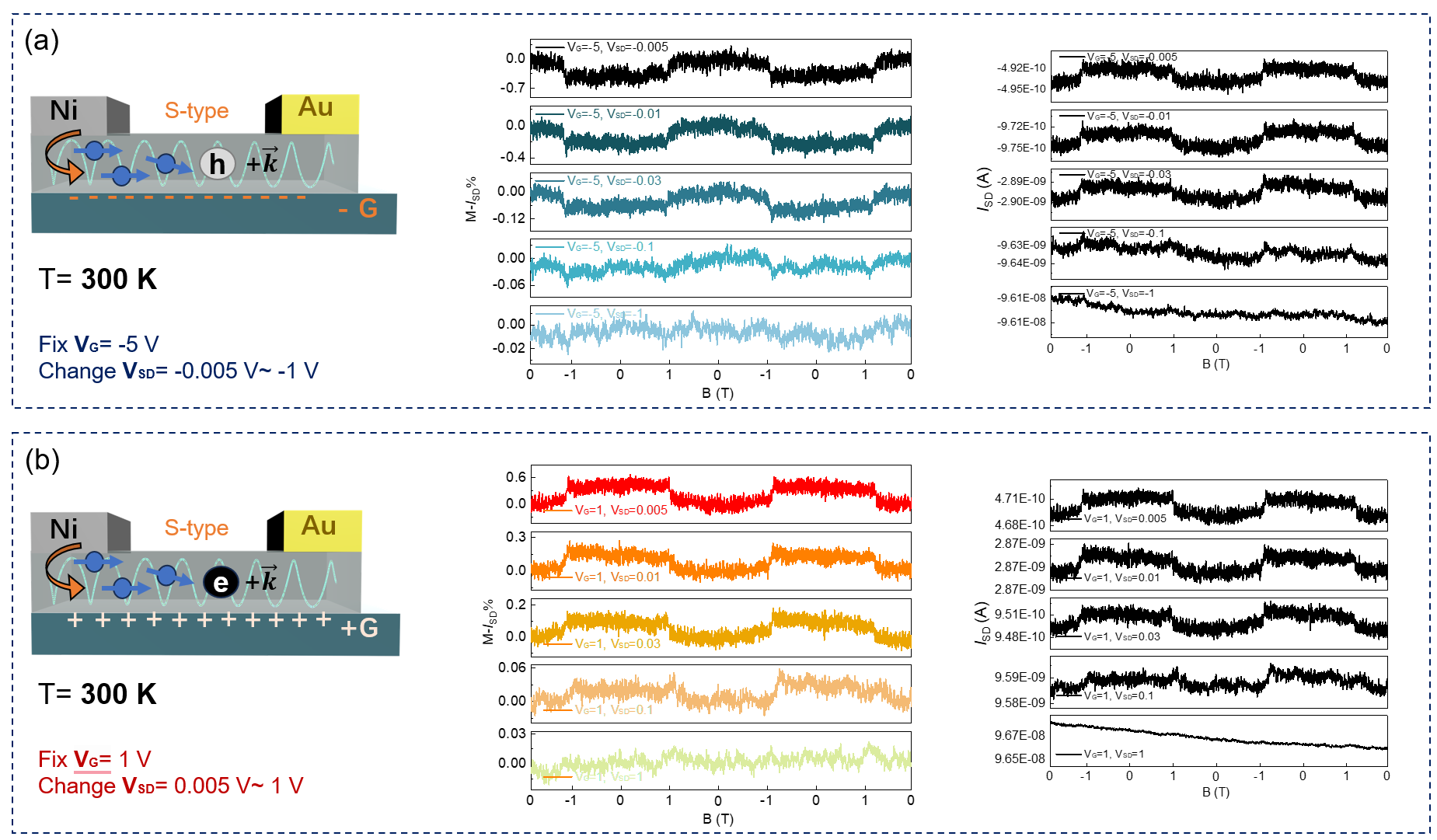


Figure S19: **(a)** **Left part**: Schematic diagram of hole transport in a FET device, the direction of the magnetic field is parallel to the direction of carrier transport. **Middle part**: The percentage change in the current of the FET with different source voltage (V_SD_) as a function of the magnetic field. **Right part**: The original curve of the current as a function of the magnetic field. **(b)** **Left part**: Schematic diagram of electron transport in a FET device, the direction of the magnetic field is parallel to the direction of carrier transport. **Middle part**: The percentage change in the current of the FET with different source voltage (V_SD_) as a function of the magnetic field. **Right part**: The original curve of the current as a function of the magnetic field.

The temperature is 300K.


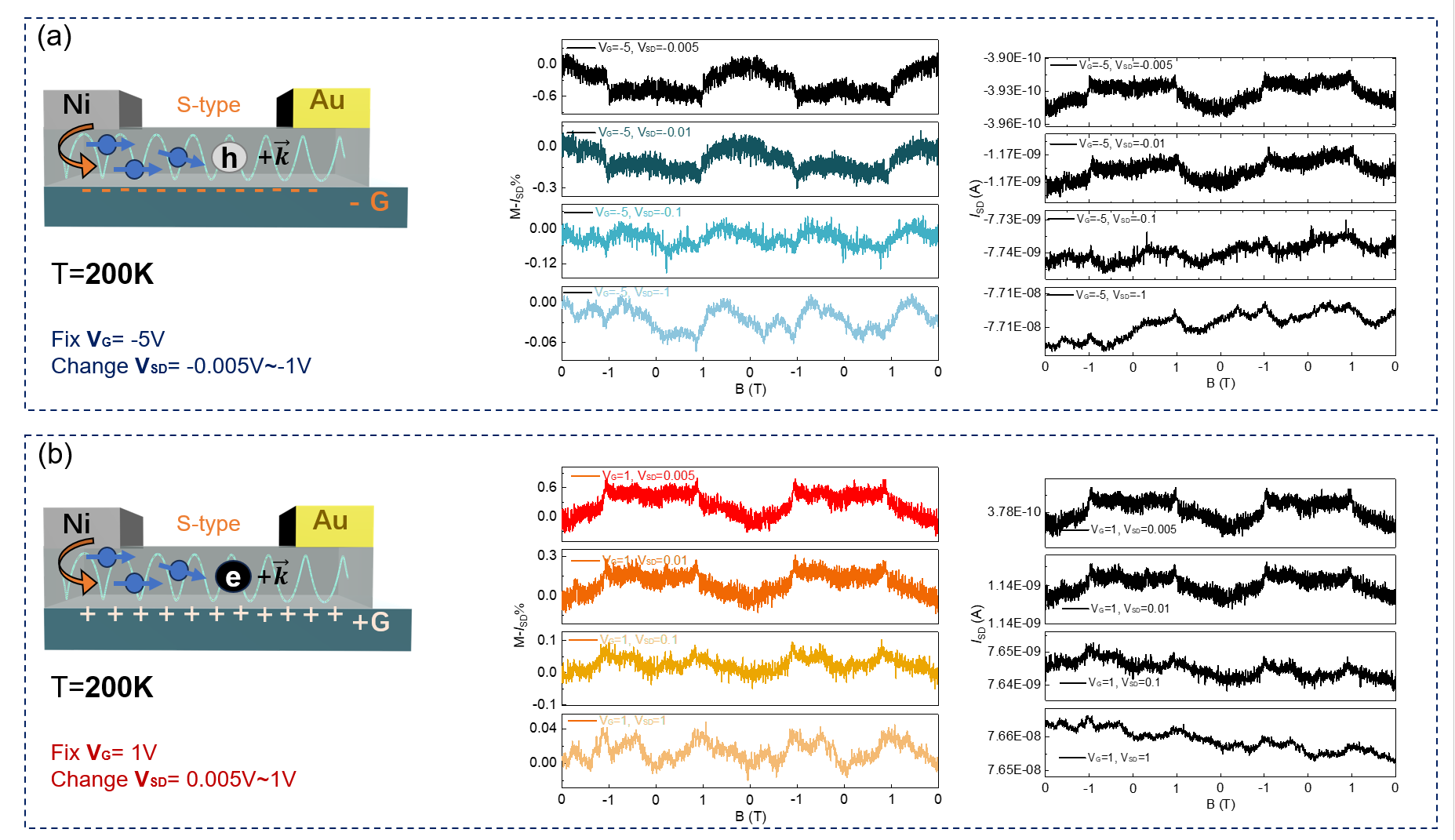


Figure S20: **(a)** **Left part**: Schematic diagram of hole transport in a FET device, the direction of the magnetic field is parallel to the direction of carrier transport. **Middle part**: The percentage change in the current of the FET with different source voltage (V_SD_) as a function of the magnetic field. **Right part**: The original curve of the current as a function of the magnetic field. **(b)** **Left part**: Schematic diagram of electron transport in a FET device, the direction of the magnetic field is parallel to the direction of carrier transport. **Middle part**: The percentage change in the current of the FET with different source voltage (V_SD_) as a function of the magnetic field. **Right part**: The original curve of the current as a function of the magnetic field.

The temperature is 200 K.


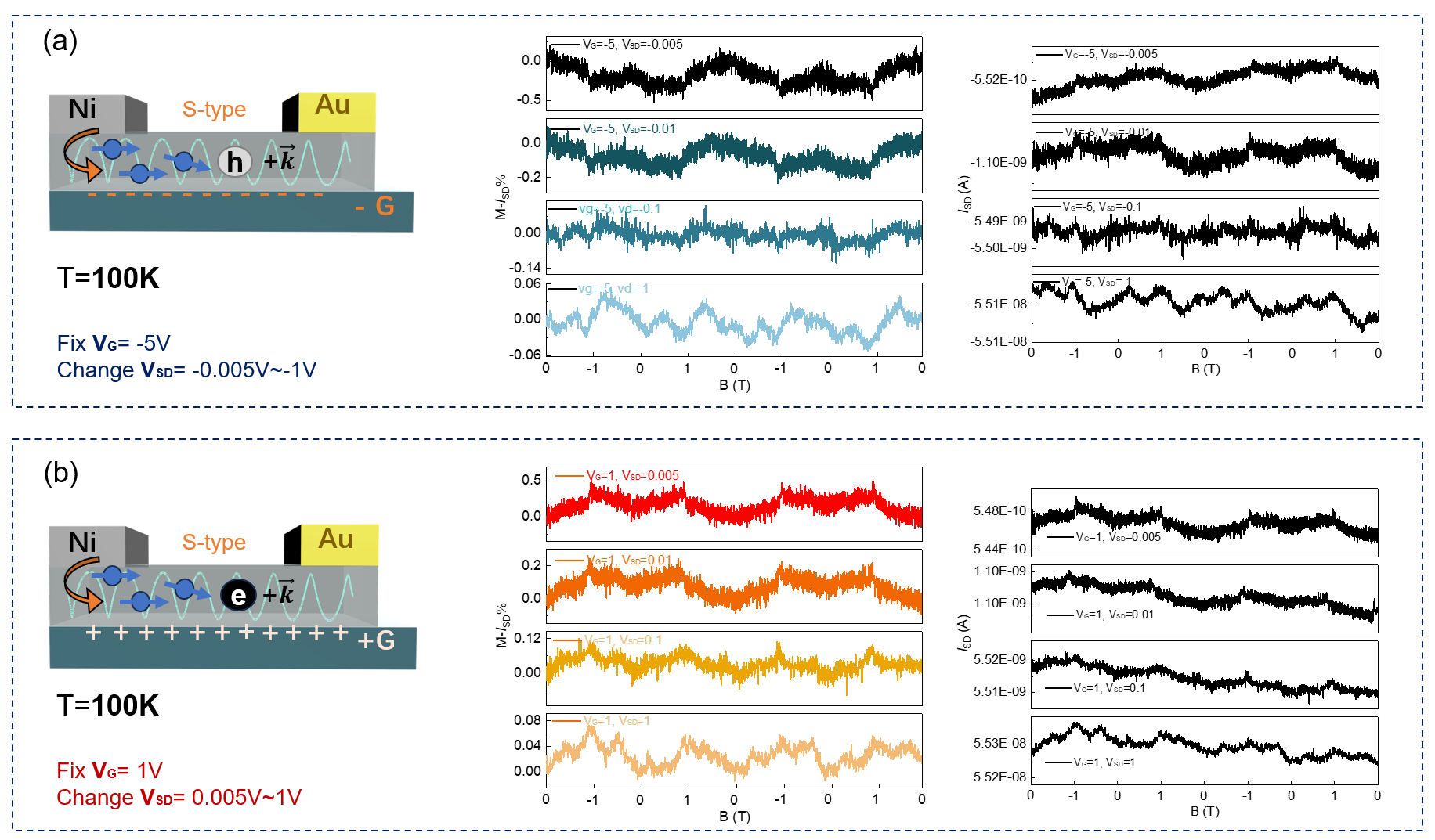


Figure S21: **(a)** **Left part**: Schematic diagram of hole transport in a FET device, the direction of the magnetic field is parallel to the direction of carrier transport. **Middle part**: The percentage change in the current of the FET with different source voltage (V_SD_) as a function of the magnetic field. **Right part**: The original curve of the current as a function of the magnetic field. **(b)** **Left part**: Schematic diagram of electron transport in a FET device, the direction of the magnetic field is parallel to the direction of carrier transport. **Middle part**: The percentage change in the current of the FET with different source voltage (V_SD_) as a function of the magnetic field. **Right part**: The original curve of the current as a function of the magnetic field.

The temperature is 100 K.


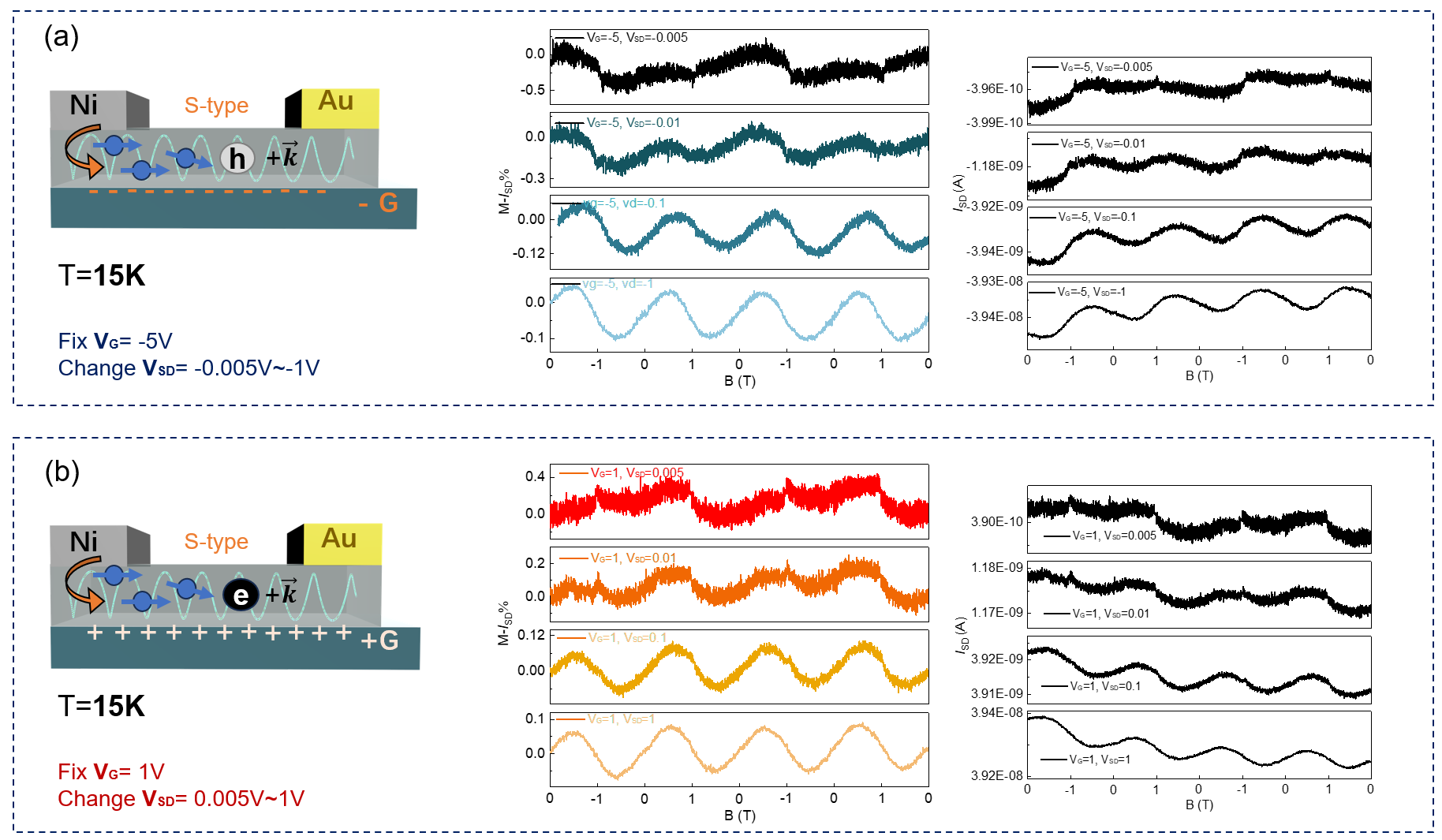


Figure S22: **(a)** **Left part**: Schematic diagram of hole transport in a FET device, the direction of the magnetic field is parallel to the direction of carrier transport. **Middle part**: The percentage change in the current of the FET with different source voltage (V_SD_) as a function of the magnetic field. **Right part**: The original curve of the current as a function of the magnetic field. **(b)** **Left part**: Schematic diagram of electron transport in a FET device, the direction of the magnetic field is parallel to the direction of carrier transport. **Middle part**: The percentage change in the current of the FET with different source voltage (V_SD_) as a function of the magnetic field. **Right part**: The original curve of the current as a function of the magnetic field.

The temperature is 15 K.


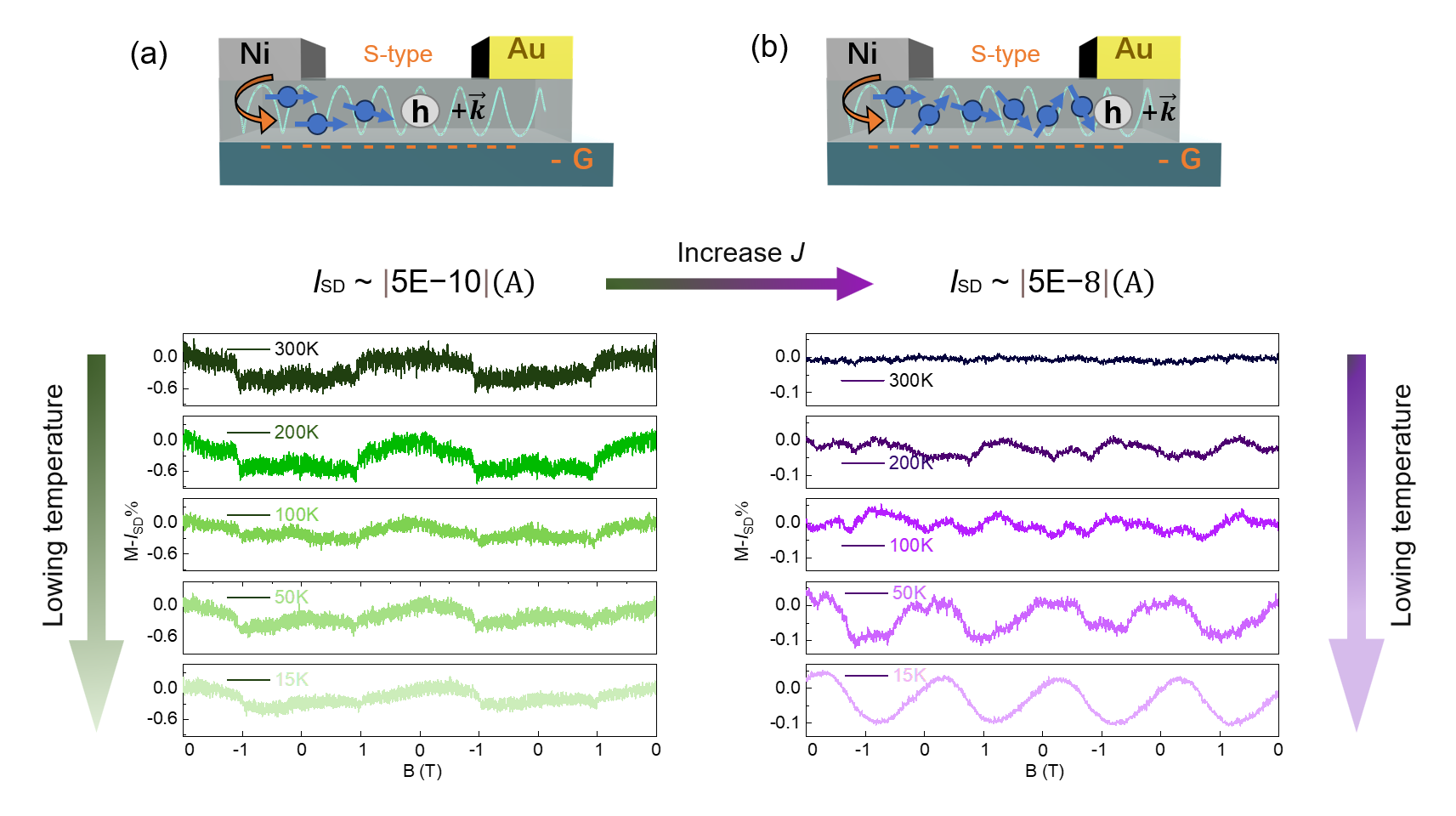


Figure S23: **(a)** At different temperatures, the percentage change of the hole current with the magnetic field, the gate voltage, and the source voltage are set to V_G_= -5 V and V_SD_= - 0.005 V, respectively, to control the absolute value of the current to stabilize at the level of 5E-10 A. **(b)** At different temperatures, the percentage change of the hole current with the magnetic field, the gate voltage and the source voltage are set to V_G_= -5 V and V_SD_= -1 V, respectively, to control the absolute value of the current to stabilize at the level of 5E-8 A. The increase in the number of carriers leads to an enhanced spin-dependent scattering, which makes the magneto-conduction signal gradually transition from chiral correlation to chiral independence

.


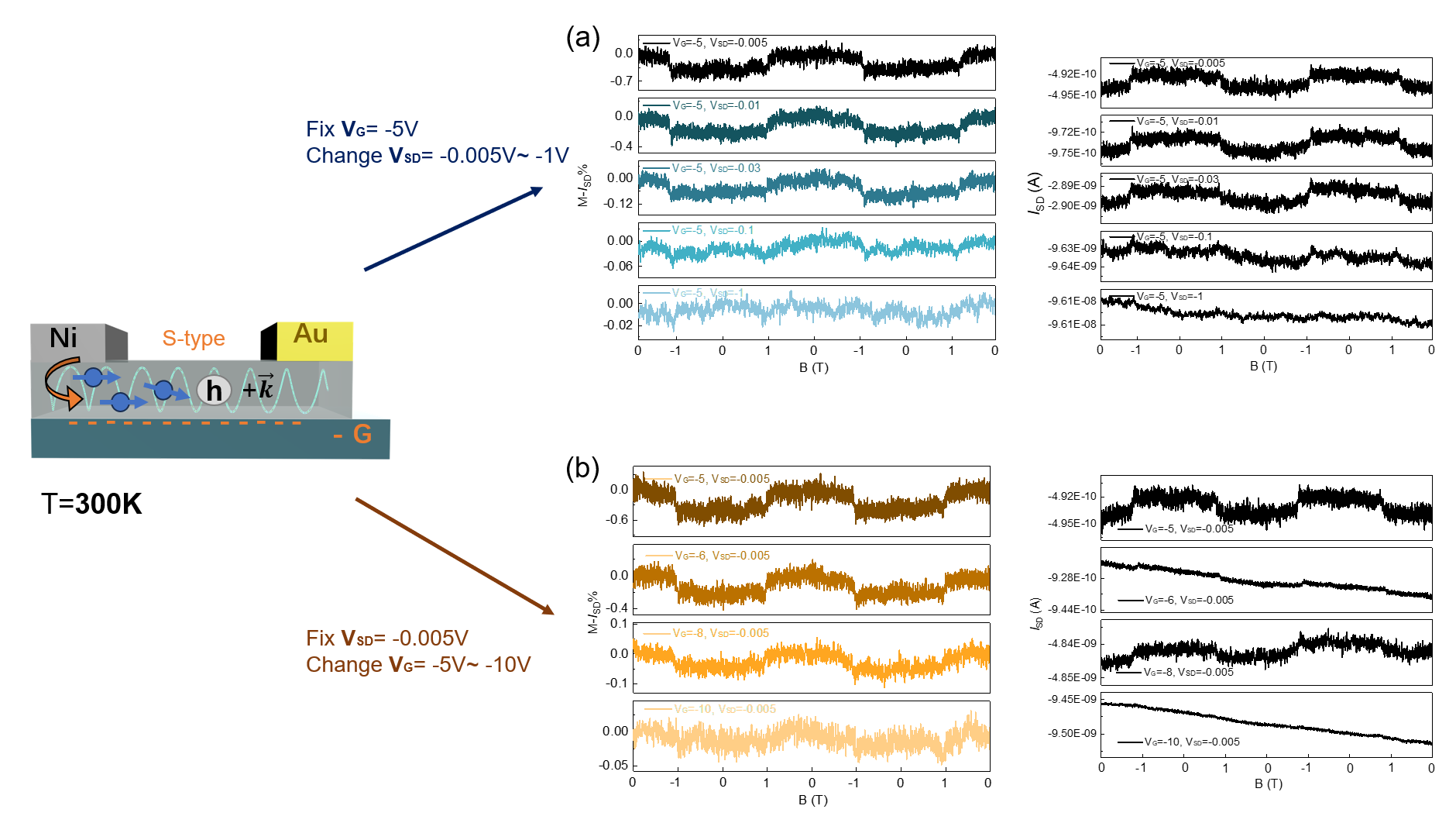


Figure S24: Comparison of the CMC signal controlled by **(a)** source voltage, and controlled by **(b)** gate voltage. Both conditions result in attenuation of the chiral-dependent MC signal. The temperature is 300 K.

**Figures S25-29: S-type device, carriers are injected from the Au electrode**


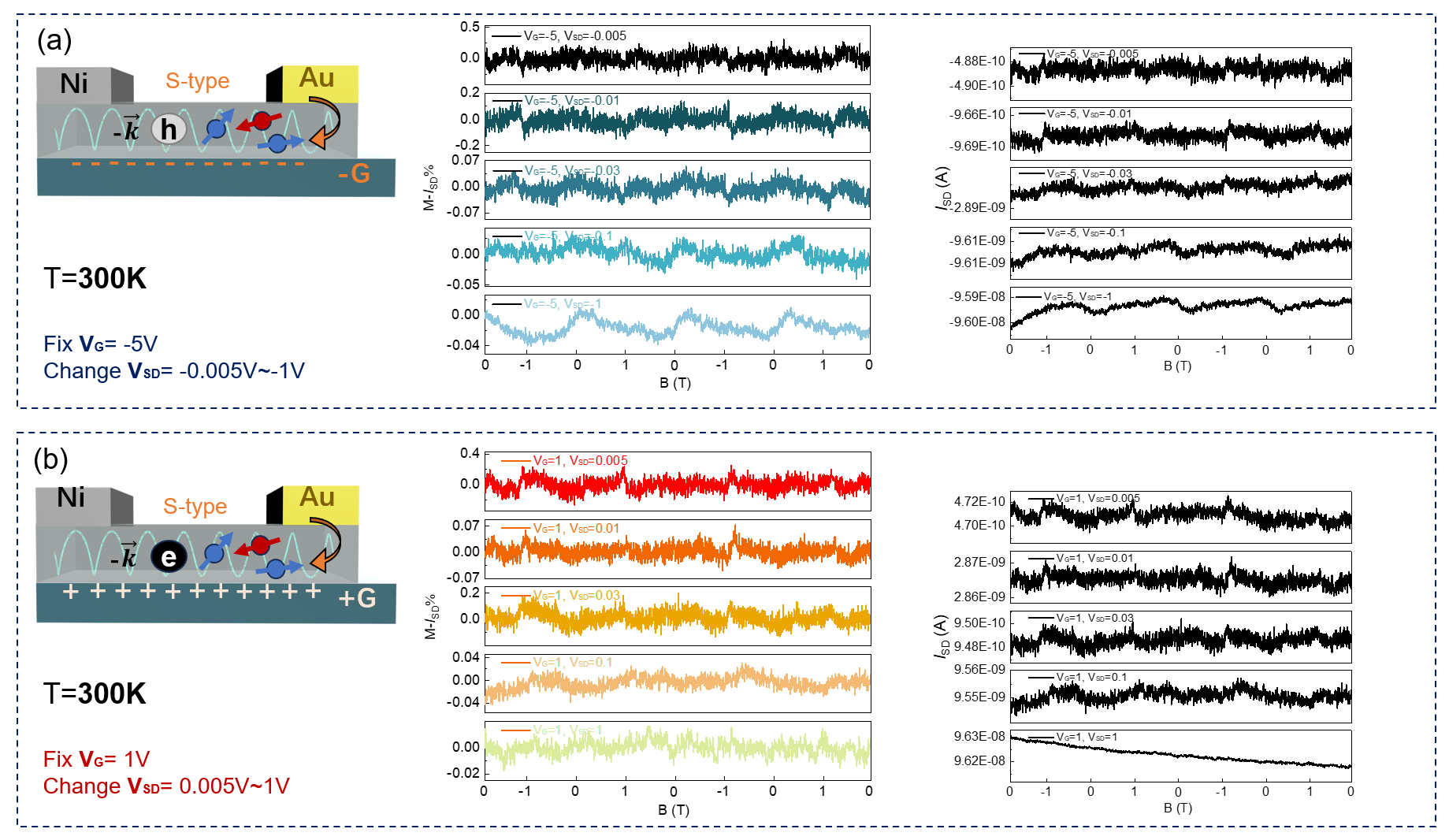


Figure S25: **(a)** **Left part**: Schematic diagram of hole transport in a FET device, the direction of the magnetic field is parallel to the direction of carrier transport. **Middle part**: The percentage change in the current of the FET with different source voltage (V_SD_) as a function of the magnetic field. **Right part**: The original curve of the current as a function of the magnetic field. **(b)** **Left part**: Schematic diagram of electron transport in a FET device, the direction of the magnetic field is parallel to the direction of carrier transport. **Middle part**: The percentage change in the current of the FET with different source voltage (V_SD_) as a function of the magnetic field. **Right part**: The original curve of the current as a function of the magnetic field.

The temperature is 300 K.


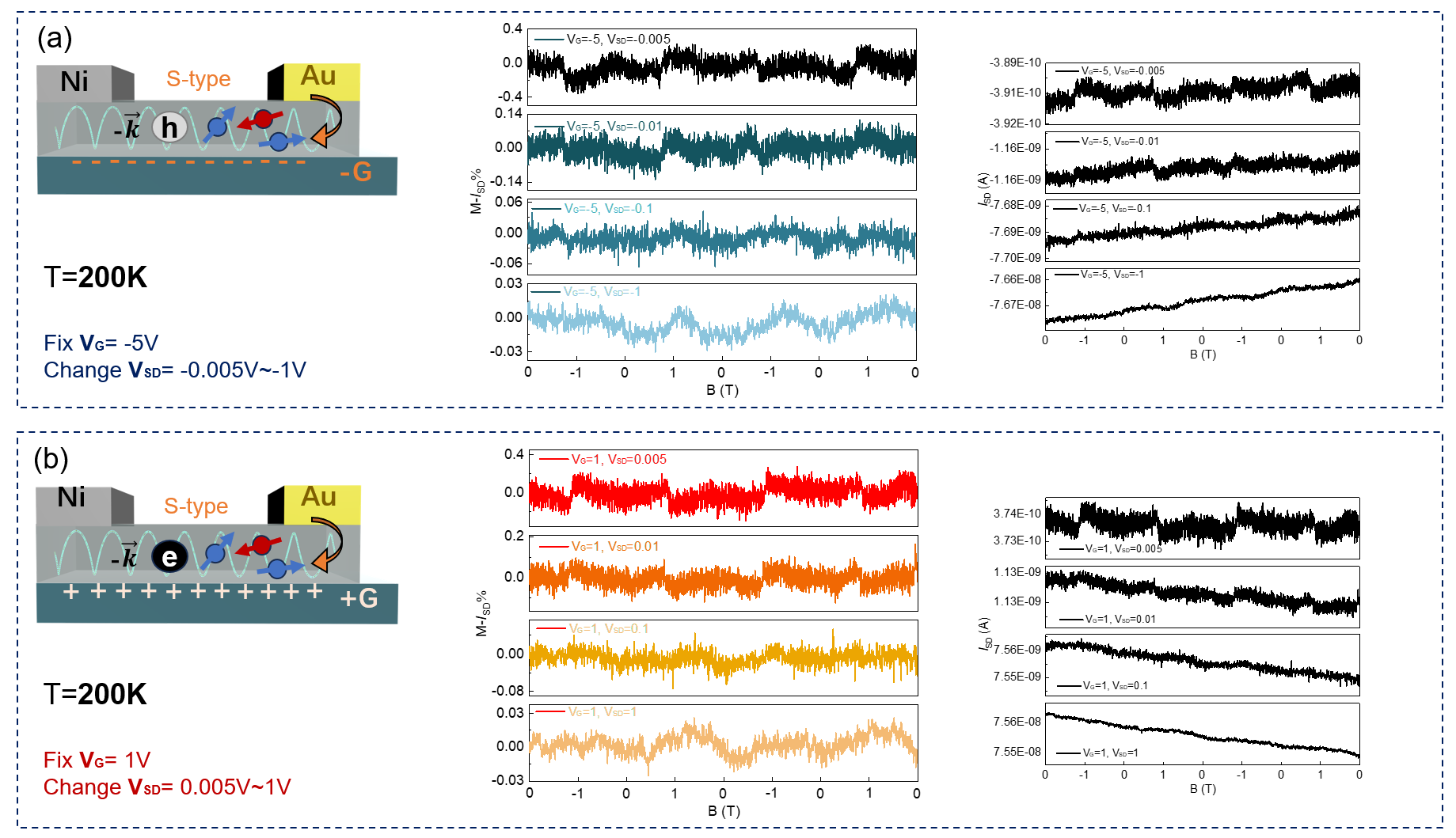


Figure S26: **(a)** **Left part**: Schematic diagram of hole transport in a FET device, the direction of the magnetic field is parallel to the direction of carrier transport. **Middle part**: The percentage change in the current of the FET with different source voltage (V_SD_) as a function of the magnetic field. **Right part**: The original curve of the current as a function of the magnetic field. **(b)** **Left part**: Schematic diagram of electron transport in a FET device, the direction of the magnetic field is parallel to the direction of carrier transport. **Middle part**: The percentage change in the current of the FET with different source voltage (V_SD_) as a function of the magnetic field. **Right part**: The original curve of the current as a function of the magnetic field.

The temperature is 200 K.


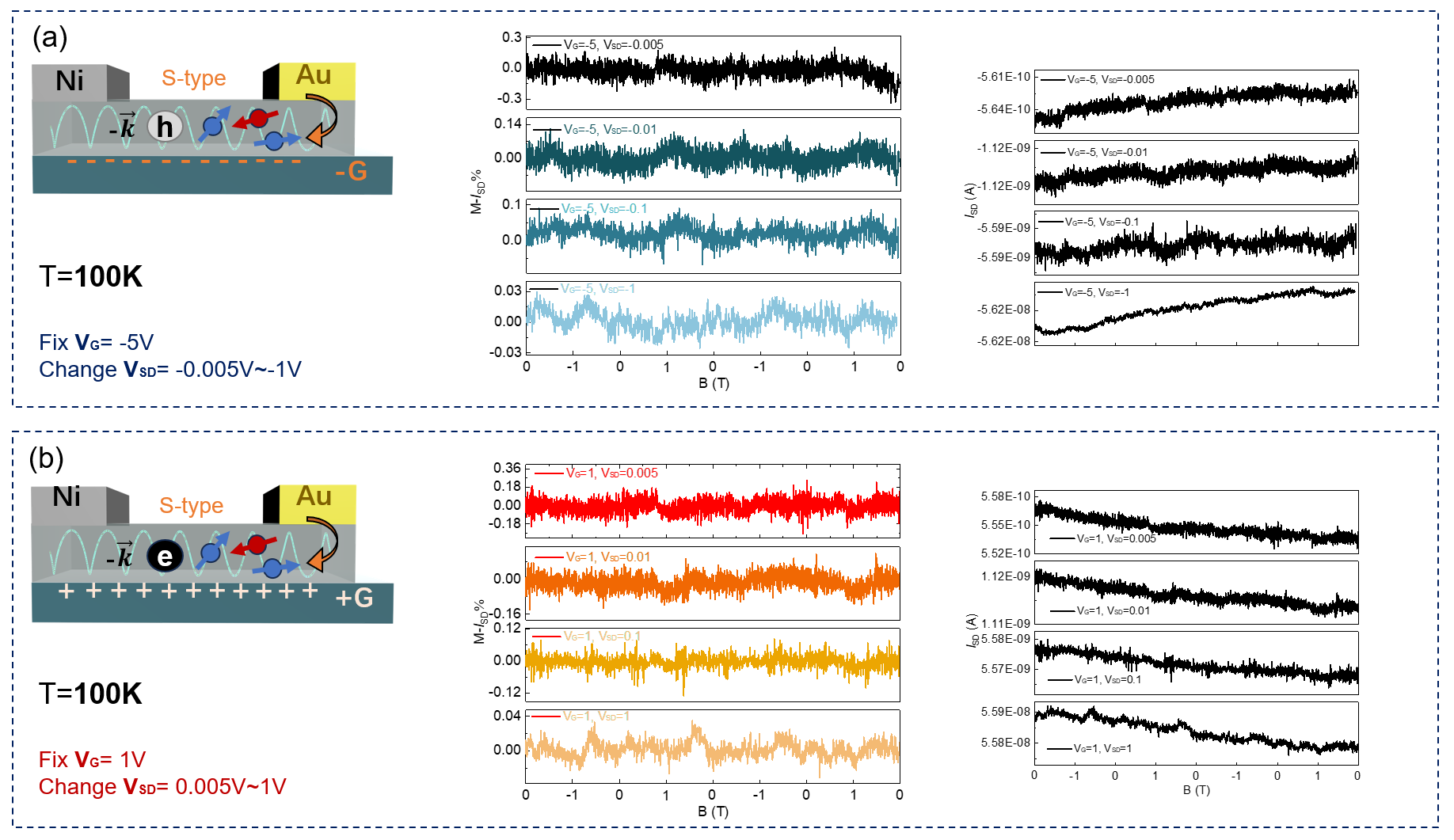


Figure S27: **(a)** **Left part**: Schematic diagram of hole transport in a FET device, the direction of the magnetic field is parallel to the direction of carrier transport. **Middle part**: The percentage change in the current of the FET with different source voltage (V_SD_) as a function of the magnetic field. **Right part**: The original curve of the current as a function of the magnetic field. **(b)** **Left part**: Schematic diagram of electron transport in a FET device, the direction of the magnetic field is parallel to the direction of carrier transport. **Middle part**: The percentage change in the current of the FET with different source voltage (V_SD_) as a function of the magnetic field. **Right part**: The original curve of the current as a function of the magnetic field.

The temperature is 100 K.


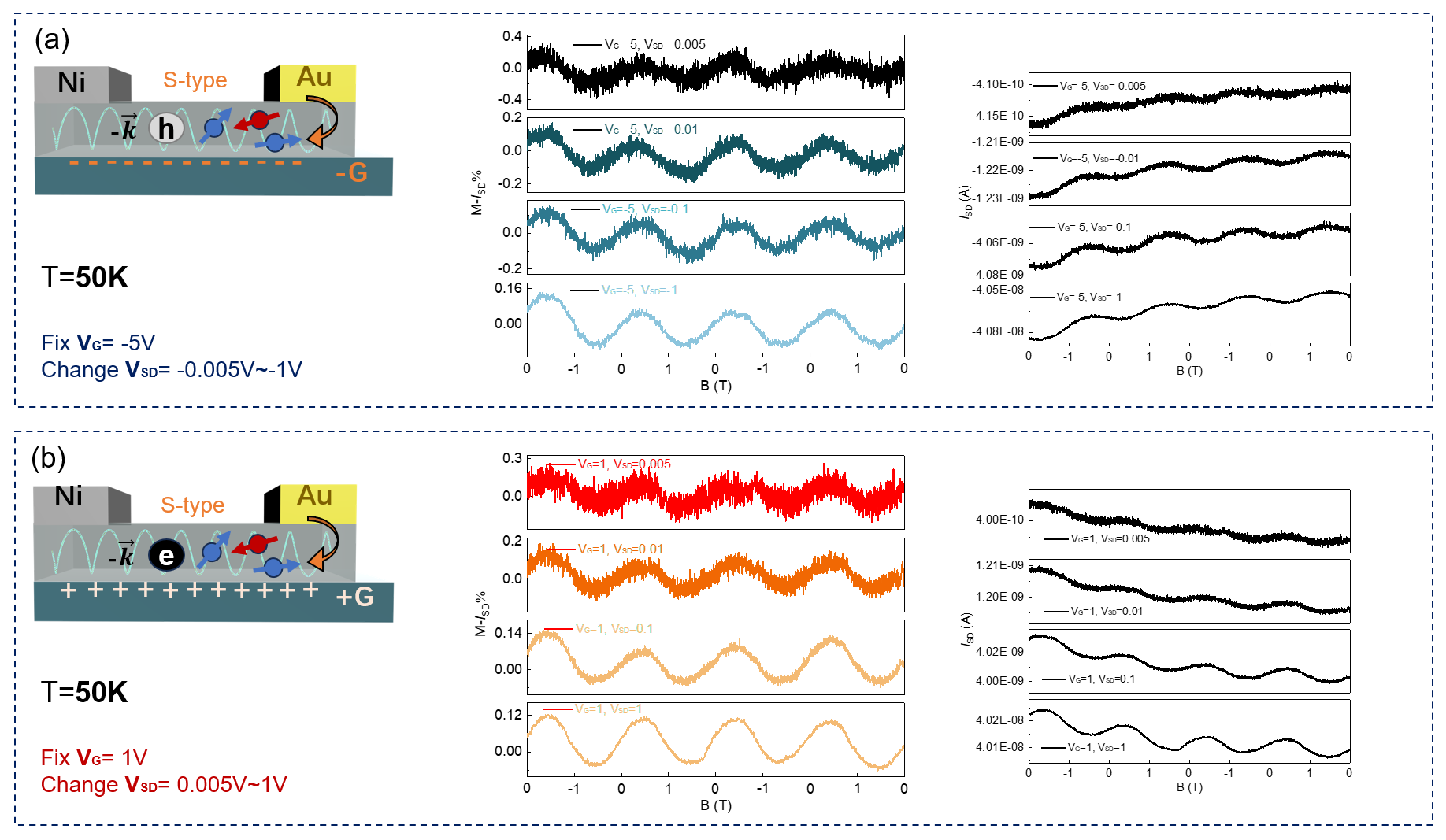


Figure S28: **(a)** **Left part**: Schematic diagram of hole transport in a FET device, the direction of the magnetic field is parallel to the direction of carrier transport. **Middle part**: The percentage change in the current of the FET with different source voltage (V_SD_) as a function of the magnetic field. **Right part**: The original curve of the current as a function of the magnetic field. **(b)** **Left part**: Schematic diagram of electron transport in a FET device, the direction of the magnetic field is parallel to the direction of carrier transport. **Middle part**: The percentage change in the current of the FET with different source voltage (V_SD_) as a function of the magnetic field. **Right part**: The original curve of the current as a function of the magnetic field.

The temperature is 50 K.


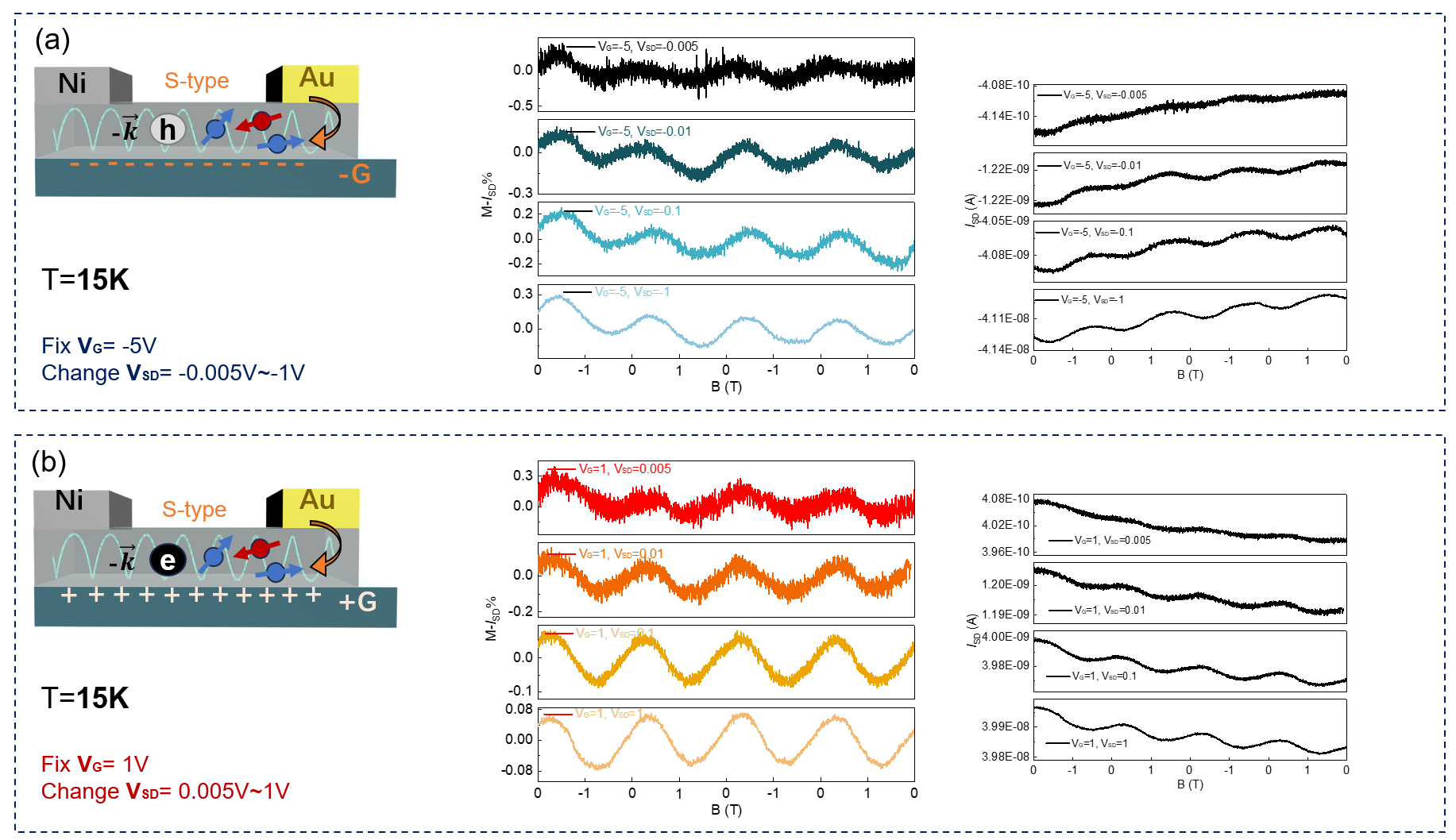


Figure S29: **(a)** **Left part**: Schematic diagram of hole transport in a FET device, the direction of the magnetic field is parallel to the direction of carrier transport. **Middle part**: The percentage change in the current of the FET with different source voltage (V_SD_) as a function of the magnetic field. **Right part**: The original curve of the current as a function of the magnetic field. **(b)** **Left part**: Schematic diagram of electron transport in a FET device, the direction of the magnetic field is parallel to the direction of carrier transport. **Middle part**: The percentage change in the current of the FET with different source voltage (V_SD_) as a function of the magnetic field. **Right part**: The original curve of the current as a function of the magnetic field.

The temperature is 15 K.

**Figures S30-34: R-type device, carriers are injected from the Ni electrode**


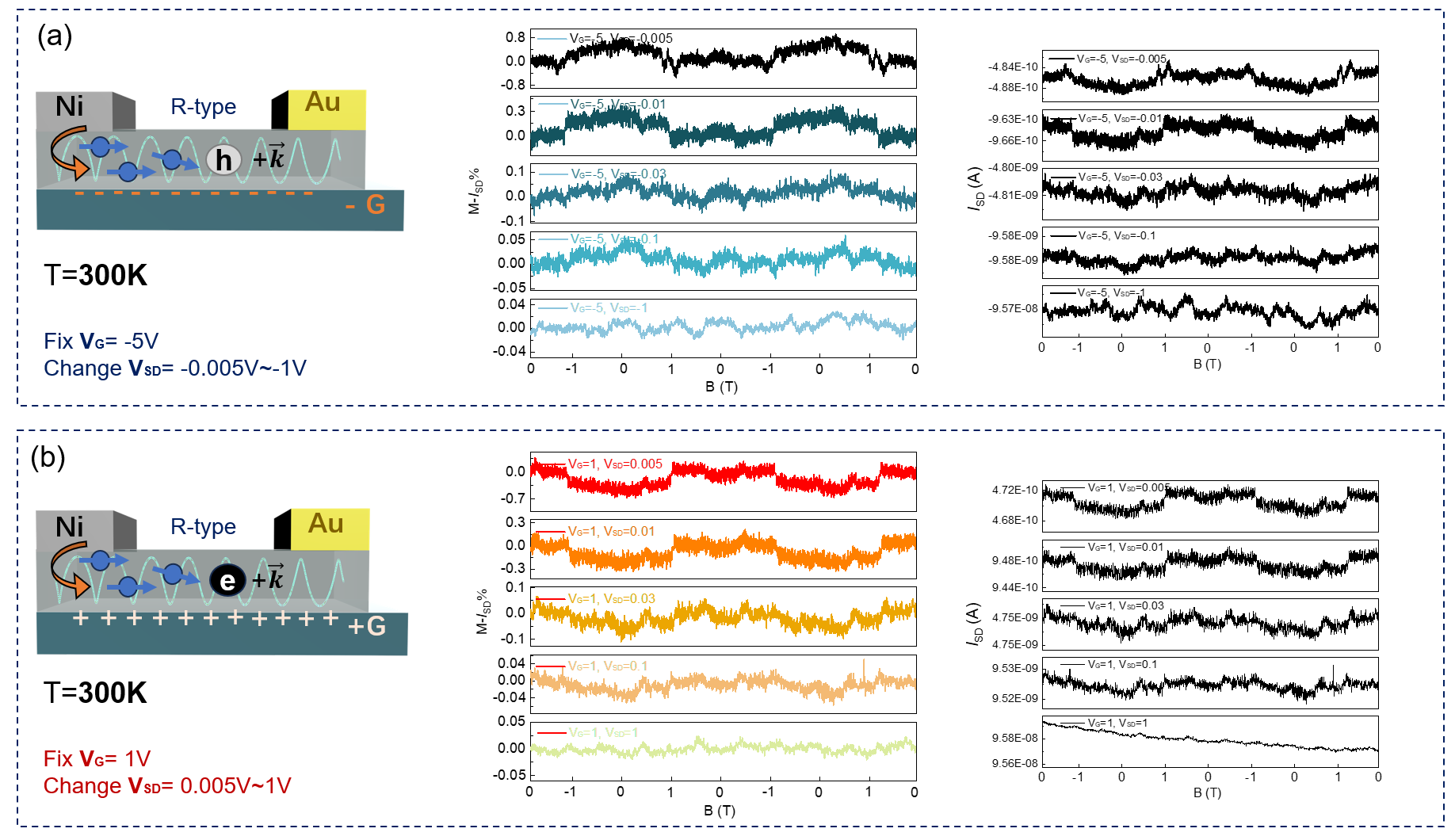


Figure S30: **(a)** **Left part**: Schematic diagram of hole transport in a FET device, the direction of the magnetic field is parallel to the direction of carrier transport. **Middle part**: The percentage change in the current of the FET with different source voltage (V_SD_) as a function of the magnetic field. **Right part**: The original curve of the current as a function of the magnetic field. **(b)** **Left part**: Schematic diagram of electron transport in a FET device, the direction of the magnetic field is parallel to the direction of carrier transport. **Middle part**: The percentage change in the current of the FET with different source voltage (V_SD_) as a function of the magnetic field. **Right part**: The original curve of the current as a function of the magnetic field.

The temperature is 300 K.


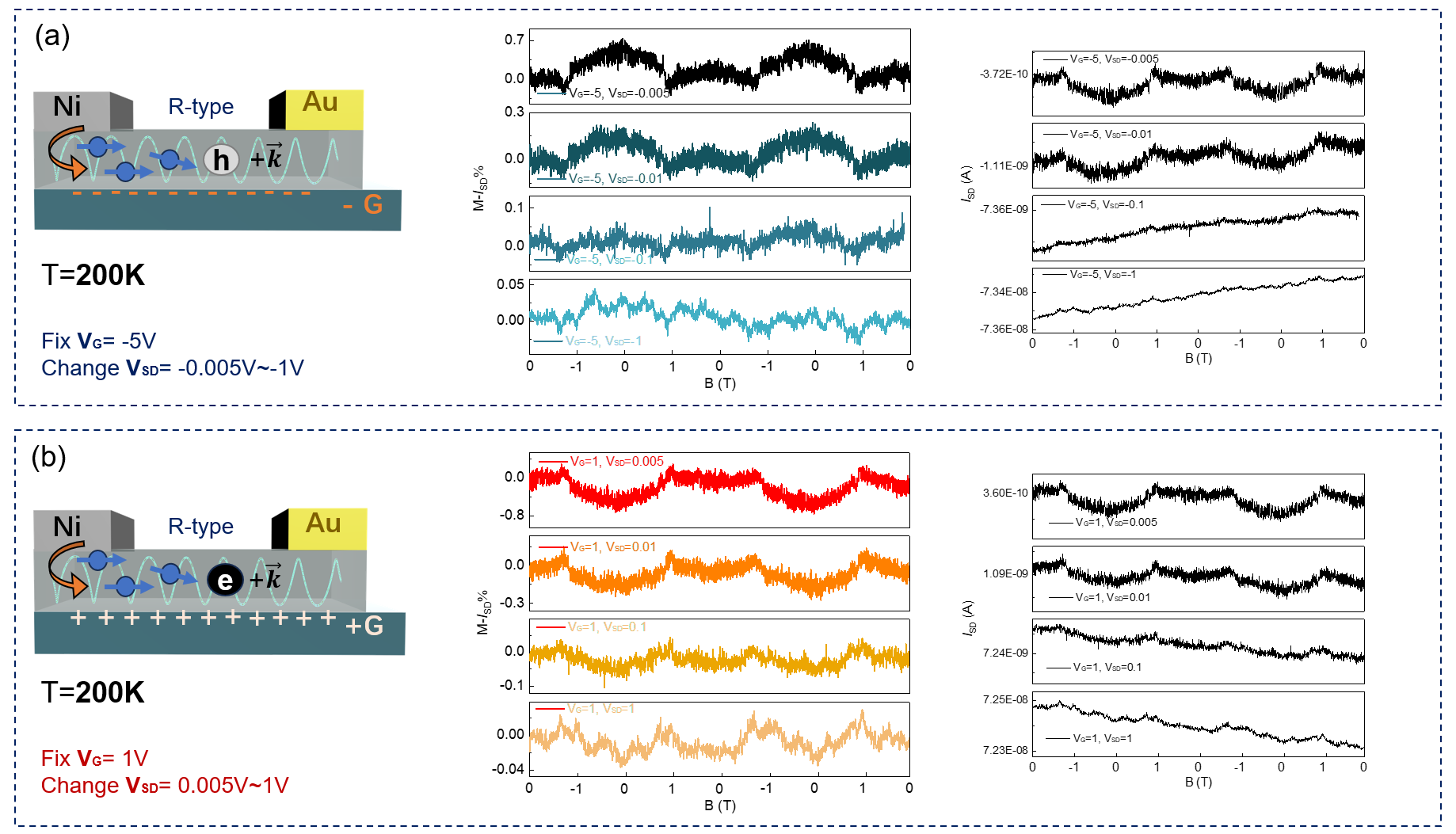


Figure S31: **(a)** **Left part**: Schematic diagram of hole transport in a FET device, the direction of the magnetic field is parallel to the direction of carrier transport. **Middle part**: The percentage change in the current of the FET with different source voltage (V_SD_) as a function of the magnetic field. **Right part**: The original curve of the current as a function of the magnetic field. **(b)** **Left part**: Schematic diagram of electron transport in a FET device, the direction of the magnetic field is parallel to the direction of carrier transport. **Middle part**: The percentage change in the current of the FET with different source voltage (V_SD_) as a function of the magnetic field. **Right part**: The original curve of the current as a function of the magnetic field.

The temperature is 200 K.


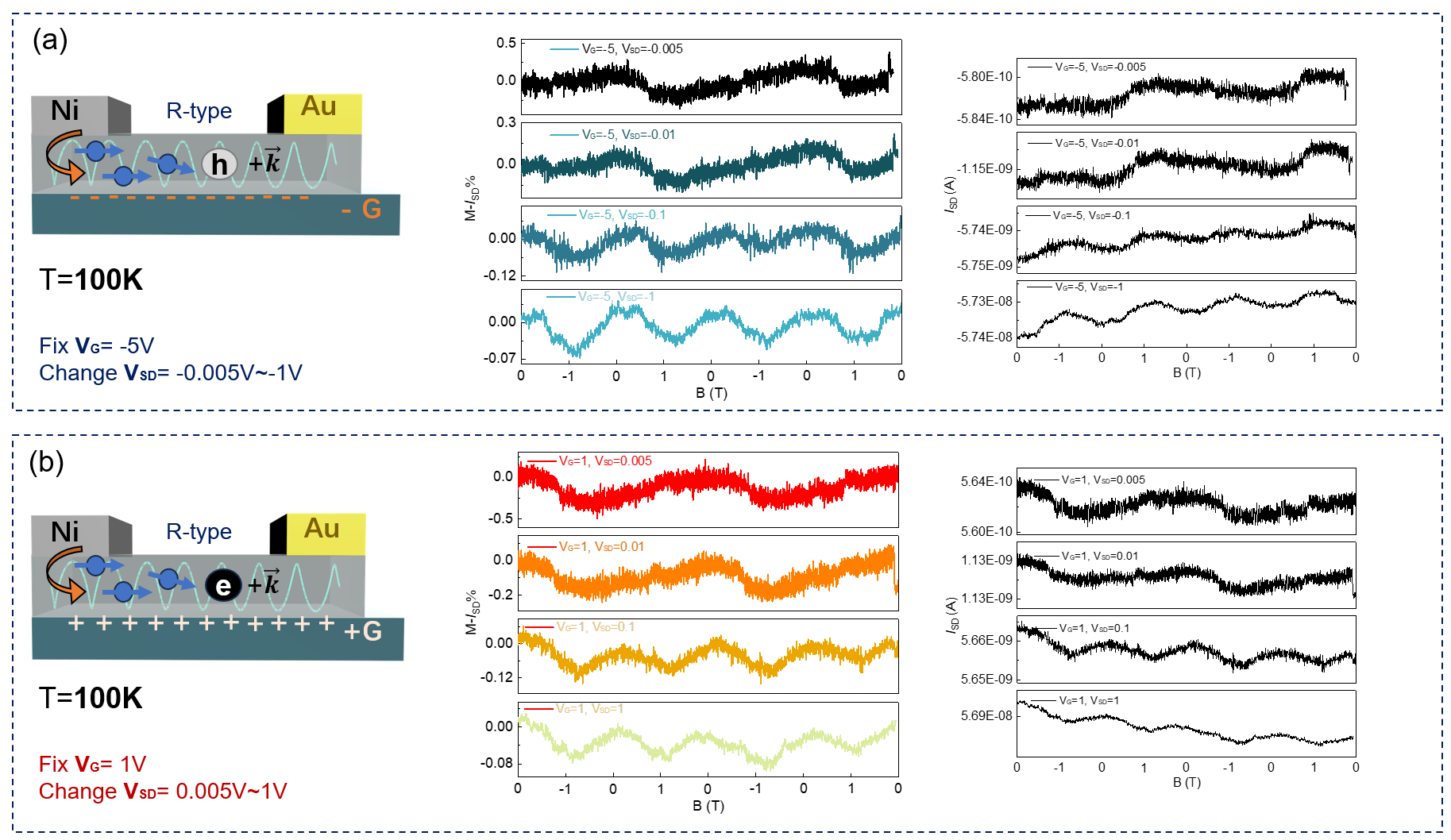


Figure S32: **(a)** **Left part**: Schematic diagram of hole transport in a FET device, the direction of the magnetic field is parallel to the direction of carrier transport. **Middle part**: The percentage change in the current of the FET with different source voltage (V_SD_) as a function of the magnetic field. **Right part**: The original curve of the current as a function of the magnetic field. **(b)** **Left part**: Schematic diagram of electron transport in a FET device, the direction of the magnetic field is parallel to the direction of carrier transport. **Middle part**: The percentage change in the current of the FET with different source voltage (V_SD_) as a function of the magnetic field. **Right part**: The original curve of the current as a function of the magnetic field.

The temperature is 100 K.


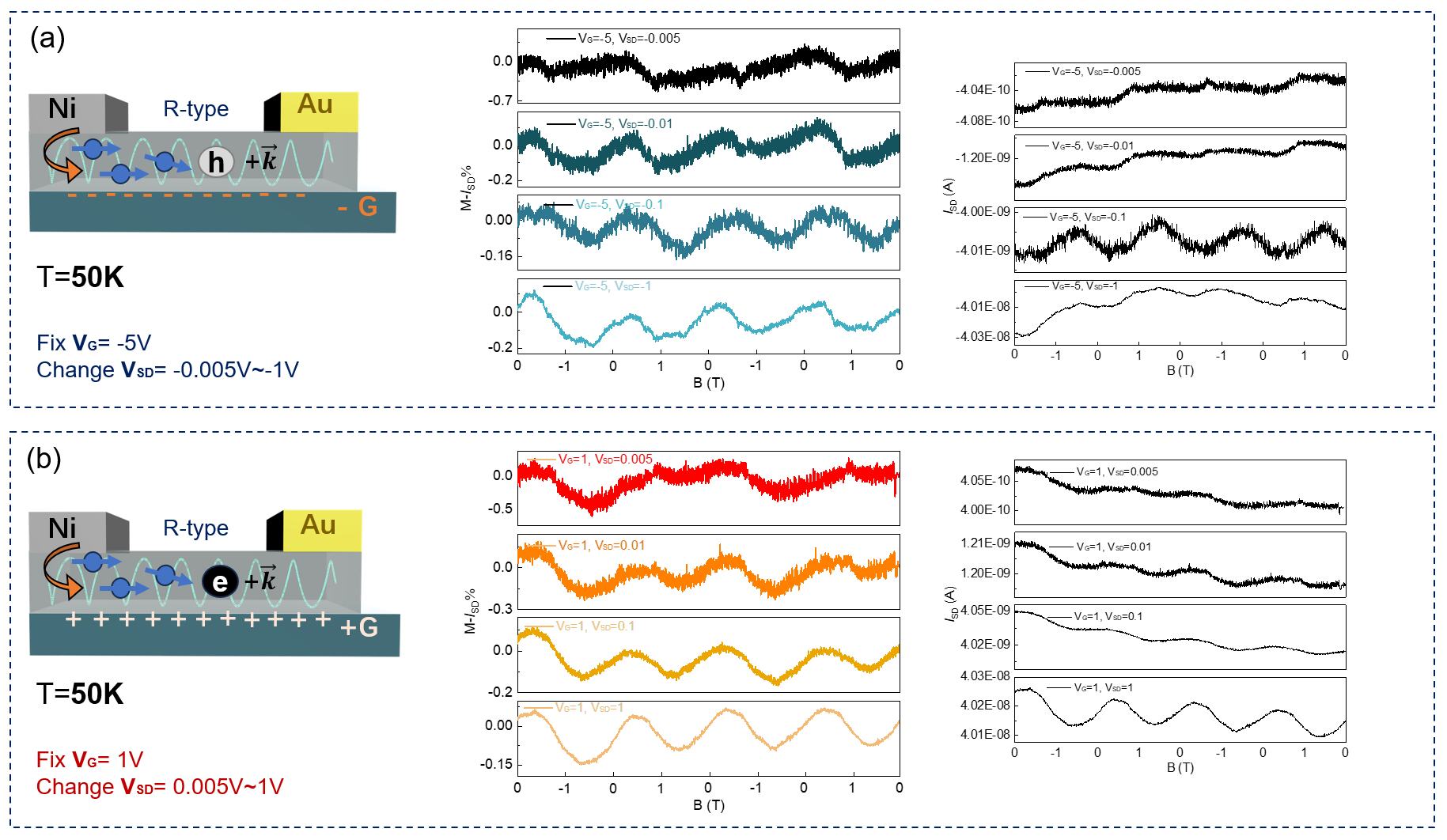


Figure S33: **(a)** **Left part**: Schematic diagram of hole transport in a FET device, the direction of the magnetic field is parallel to the direction of carrier transport. **Middle part**: The percentage change in the current of the FET with different source voltage (V_SD_) as a function of the magnetic field. **Right part**: The original curve of the current as a function of the magnetic field. **(b)** **Left part**: Schematic diagram of electron transport in a FET device, the direction of the magnetic field is parallel to the direction of carrier transport. **Middle part**: The percentage change in the current of the FET with different source voltage (V_SD_) as a function of the magnetic field. **Right part**: The original curve of the current as a function of the magnetic field.

The temperature is 50 K.


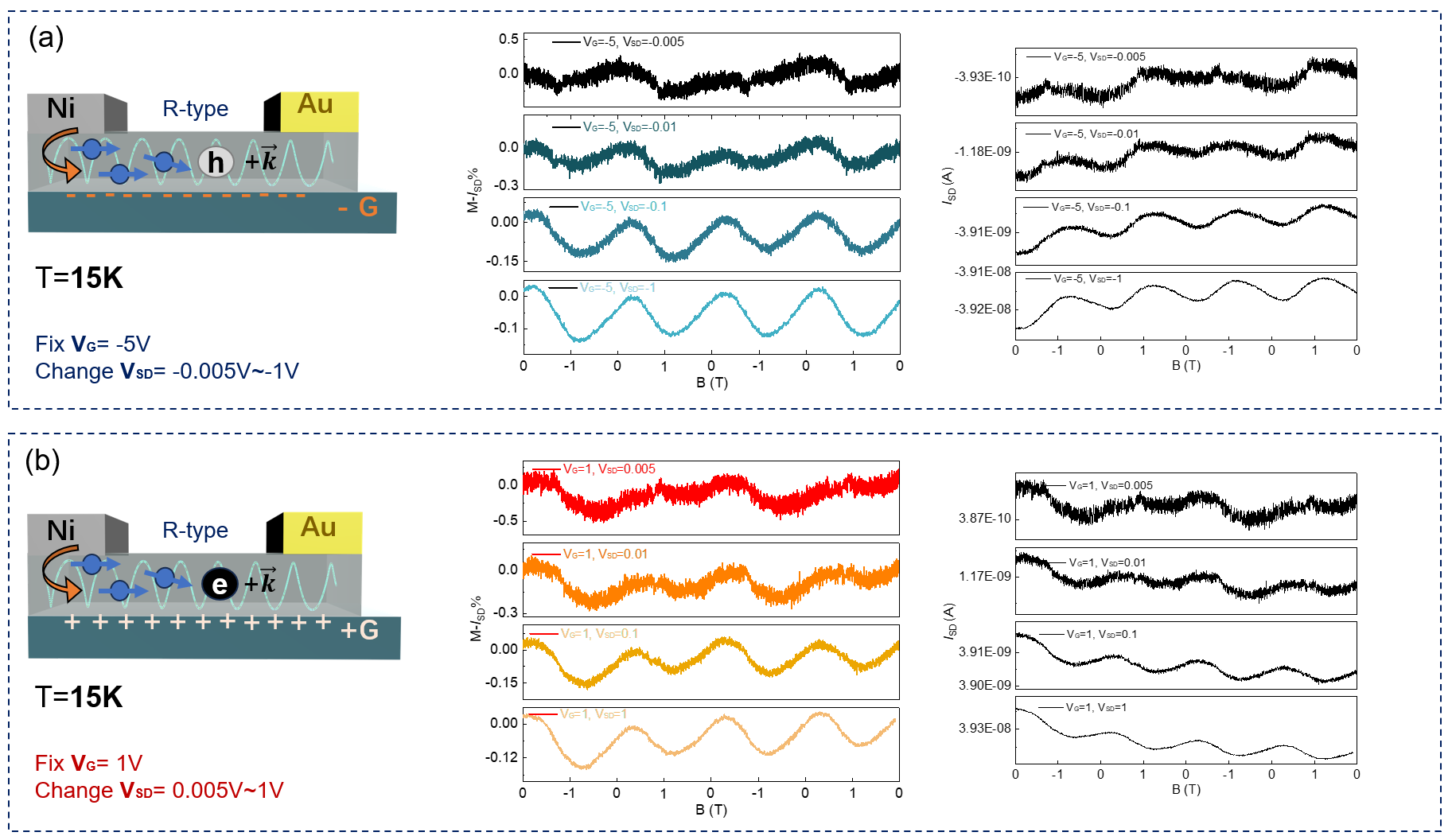


Figure S34: **(a)** **Left part**: Schematic diagram of hole transport in a FET device, the direction of the magnetic field is parallel to the direction of carrier transport. **Middle part**: The percentage change in the current of the FET with different source voltage (V_SD_) as a function of the magnetic field. **Right part**: The original curve of the current as a function of the magnetic field. **(b)** **Left part**: Schematic diagram of electron transport in a FET device, the direction of the magnetic field is parallel to the direction of carrier transport. **Middle part**: The percentage change in the current of the FET with different source voltage (V_SD_) as a function of the magnetic field. **Right part**: The original curve of the current as a function of the magnetic field.

The temperature is 15 K.

**Figures S35-39: R-type device, carriers are injected from the Au electrode**


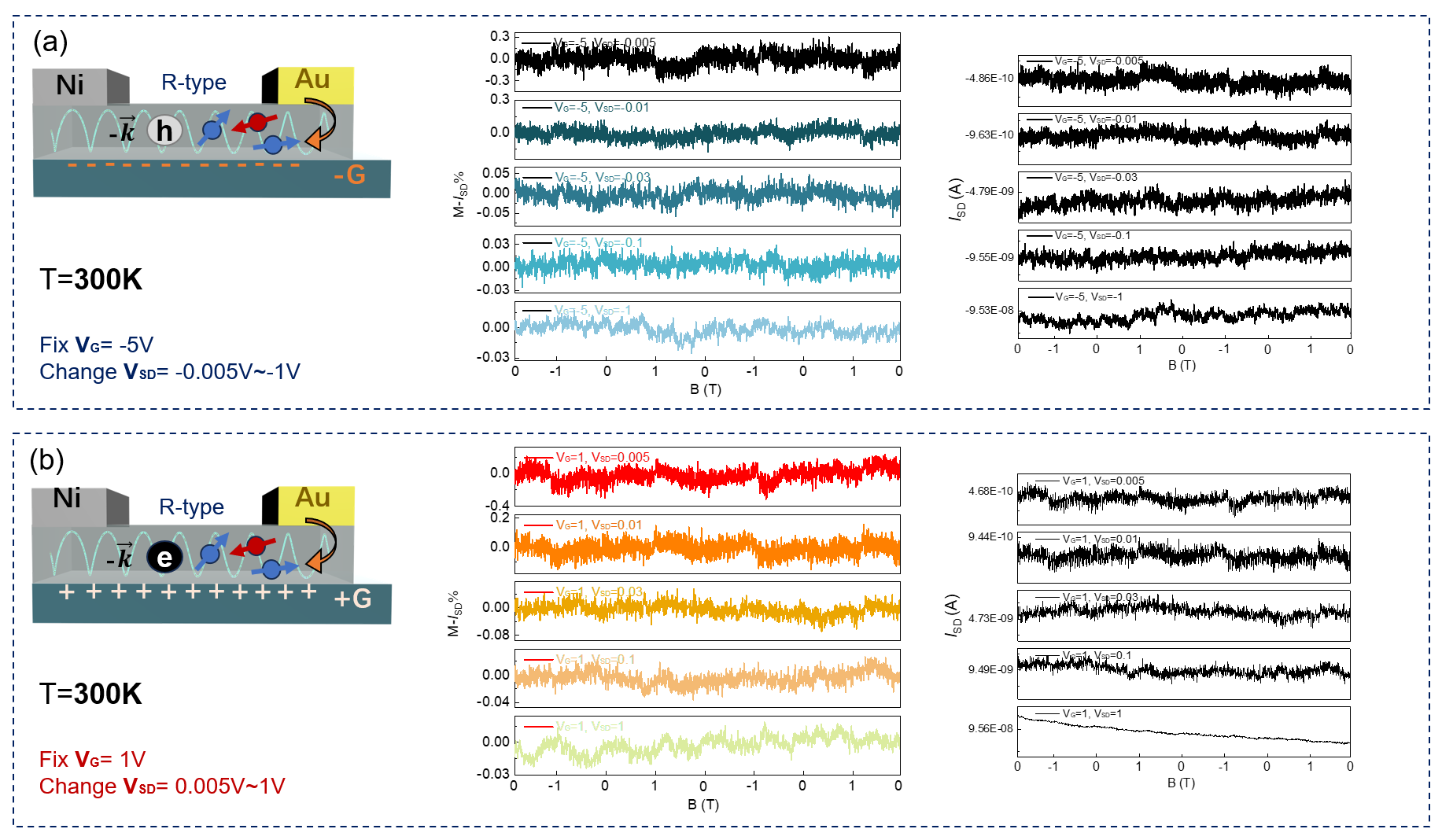


Figure S35: **(a)** **Left part**: Schematic diagram of hole transport in a FET device, the direction of the magnetic field is parallel to the direction of carrier transport. **Middle part**: The percentage change in the current of the FET with different source voltage (V_SD_) as a function of the magnetic field. **Right part**: The original curve of the current as a function of the magnetic field. **(b)** **Left part**: Schematic diagram of electron transport in a FET device, the direction of the magnetic field is parallel to the direction of carrier transport. **Middle part**: The percentage change in the current of the FET with different source voltage (V_SD_) as a function of the magnetic field. **Right part**: The original curve of the current as a function of the magnetic field.

The temperature is 300 K.


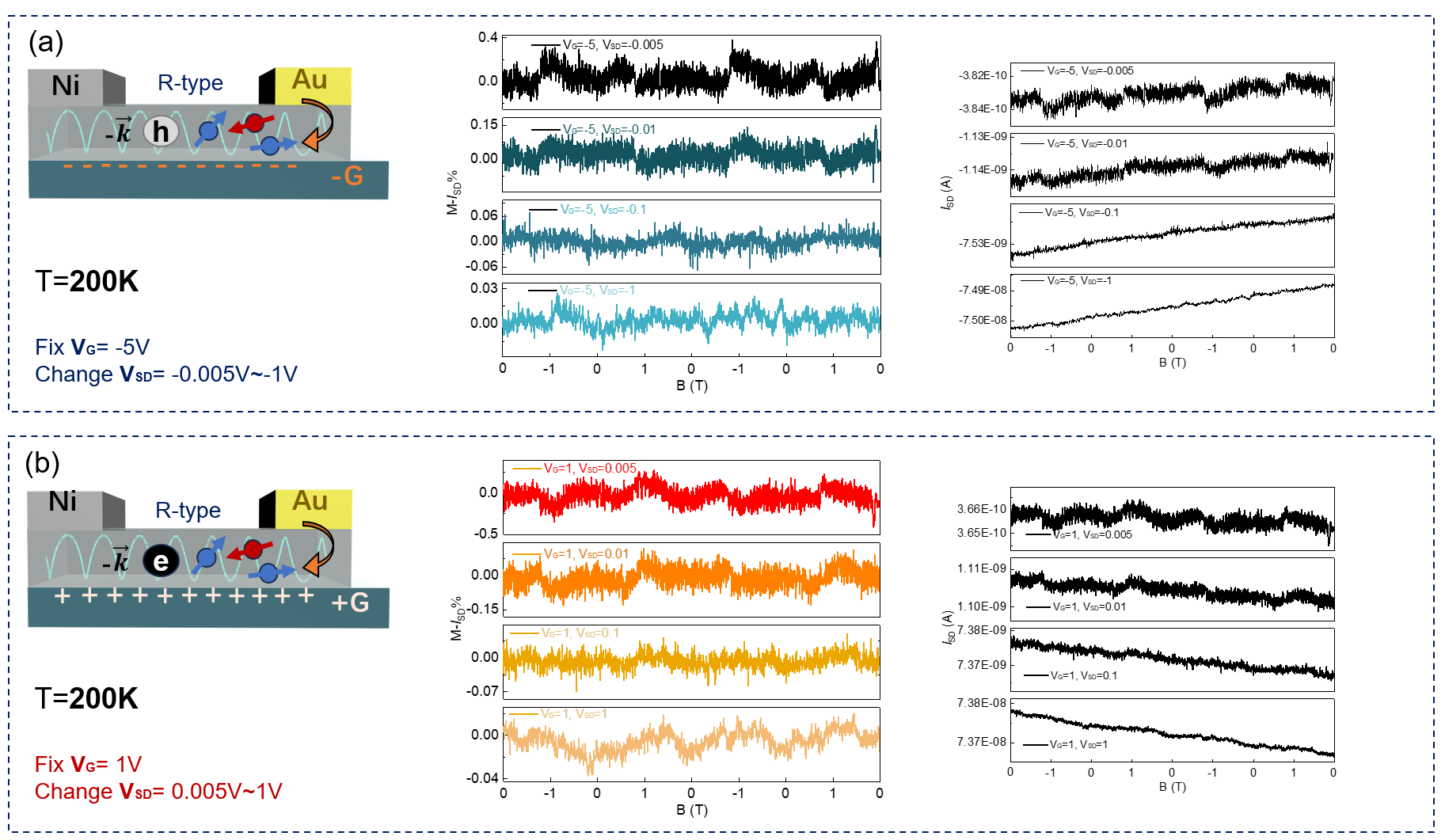


Figure S36: **(a)** **Left part**: Schematic diagram of hole transport in a FET device, the direction of the magnetic field is parallel to the direction of carrier transport. **Middle part**: The percentage change in the current of the FET with different source voltage (V_SD_) as a function of the magnetic field. **Right part**: The original curve of the current as a function of the magnetic field. **(b)** **Left part**: Schematic diagram of electron transport in a FET device, the direction of the magnetic field is parallel to the direction of carrier transport. **Middle part**: The percentage change in the current of the FET with different source voltage (V_SD_) as a function of the magnetic field. **Right part**: The original curve of the current as a function of the magnetic field.

The temperature is 200 K.


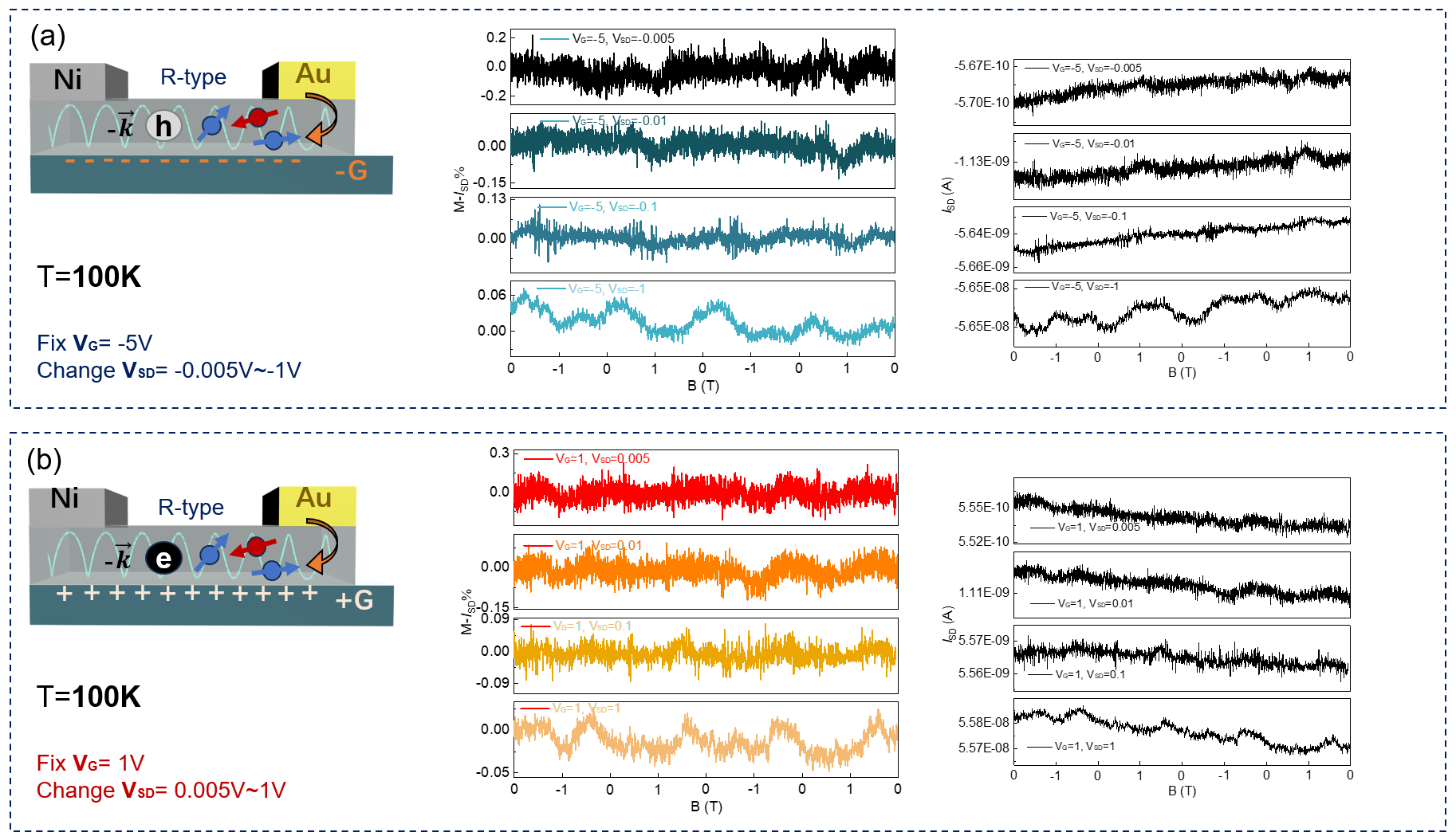


Figure S37: **(a)** **Left part**: Schematic diagram of hole transport in a FET device, the direction of the magnetic field is parallel to the direction of carrier transport. **Middle part**: The percentage change in the current of the FET with different source voltage (V_SD_) as a function of the magnetic field. **Right part**: The original curve of the current as a function of the magnetic field. **(b)** **Left part**: Schematic diagram of electron transport in a FET device, the direction of the magnetic field is parallel to the direction of carrier transport. **Middle part**: The percentage change in the current of the FET with different source voltage (V_SD_) as a function of the magnetic field. **Right part**: The original curve of the current as a function of the magnetic field.

The temperature is 100 K.


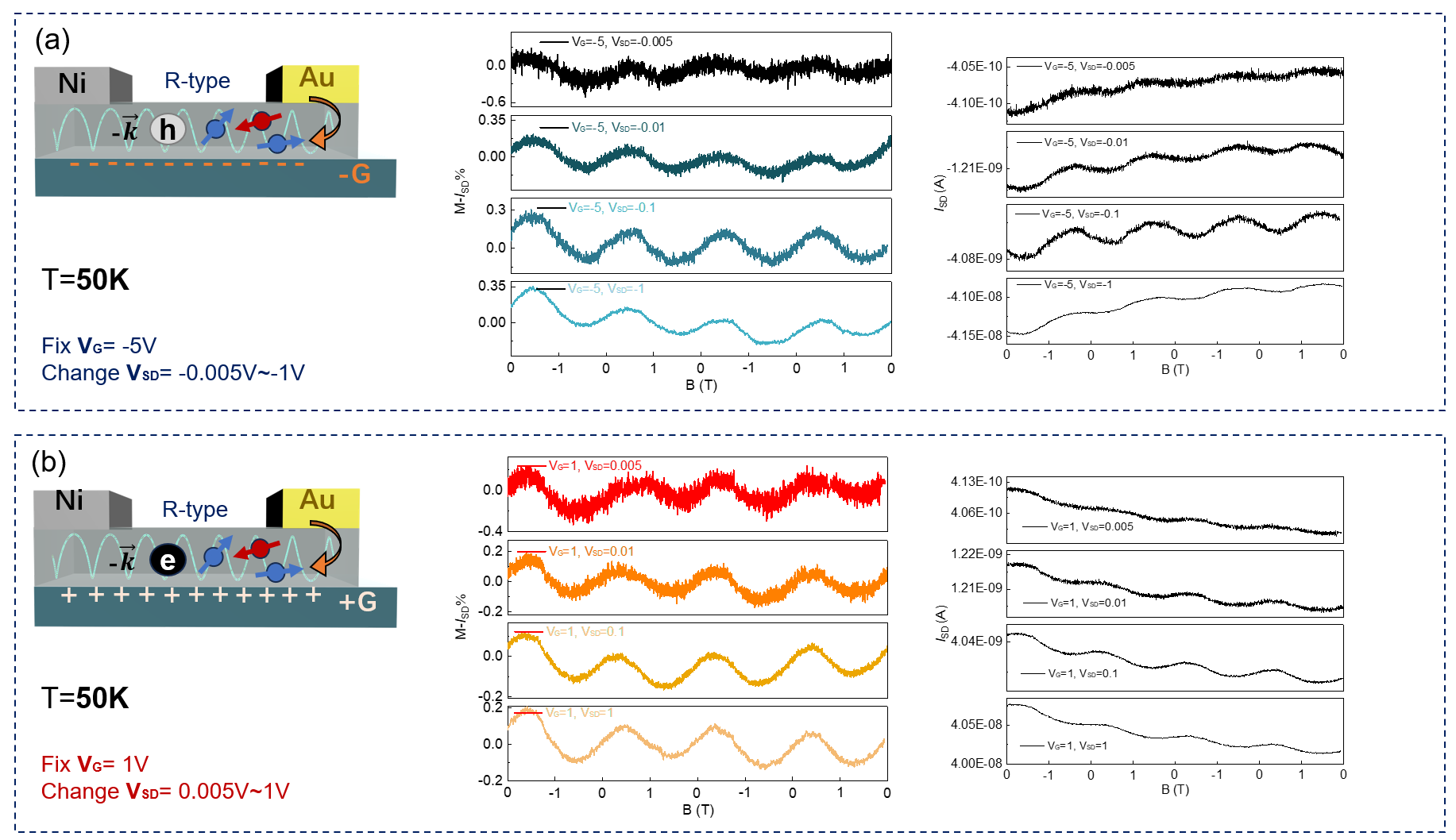


Figure S38: **(a)** **Left part**: Schematic diagram of hole transport in a FET device, the direction of the magnetic field is parallel to the direction of carrier transport. **Middle part**: The percentage change in the current of the FET with different source voltage (V_SD_) as a function of the magnetic field. **Right part**: The original curve of the current as a function of the magnetic field. **(b)** **Left part**: Schematic diagram of electron transport in a FET device, the direction of the magnetic field is parallel to the direction of carrier transport. **Middle part**: The percentage change in the current of the FET with different source voltage (V_SD_) as a function of the magnetic field. **Right part**: The original curve of the current as a function of the magnetic field.

The temperature is 50 K.


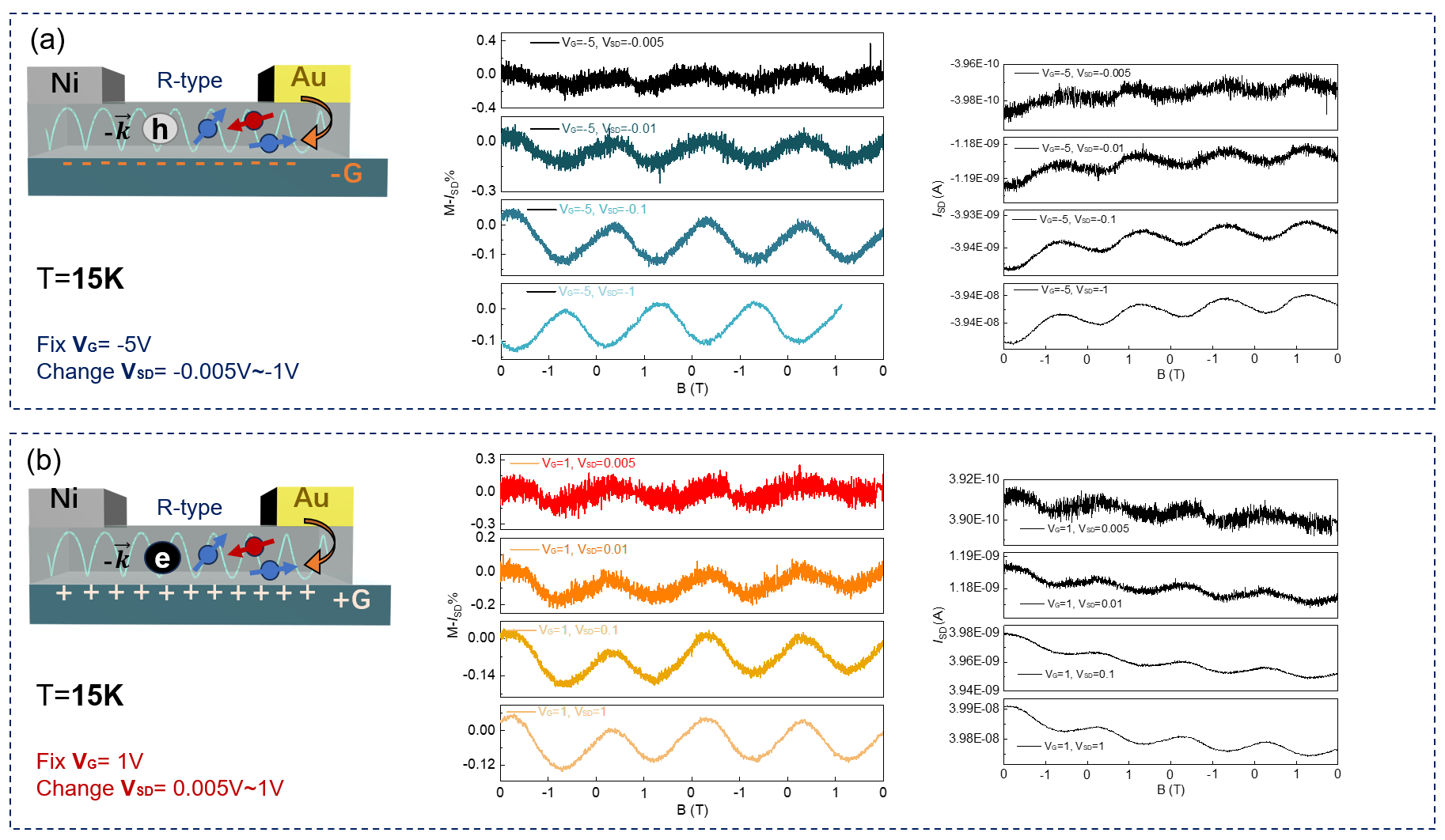


Figure S39: **(a)** **Left part**: Schematic diagram of hole transport in a FET device, the direction of the magnetic field is parallel to the direction of carrier transport. **Middle part**: The percentage change in the current of the FET with different source voltage (V_SD_) as a function of the magnetic field. **Right part**: The original curve of the current as a function of the magnetic field. **(b)** **Left part**: Schematic diagram of electron transport in a FET device, the direction of the magnetic field is parallel to the direction of carrier transport. **Middle part**: The percentage change in the current of the FET with different source voltage (V_SD_) as a function of the magnetic field. **Right part**: The original curve of the current as a function of the magnetic field.

The temperature is 15 K.

**Figures S40-41: A-type device**


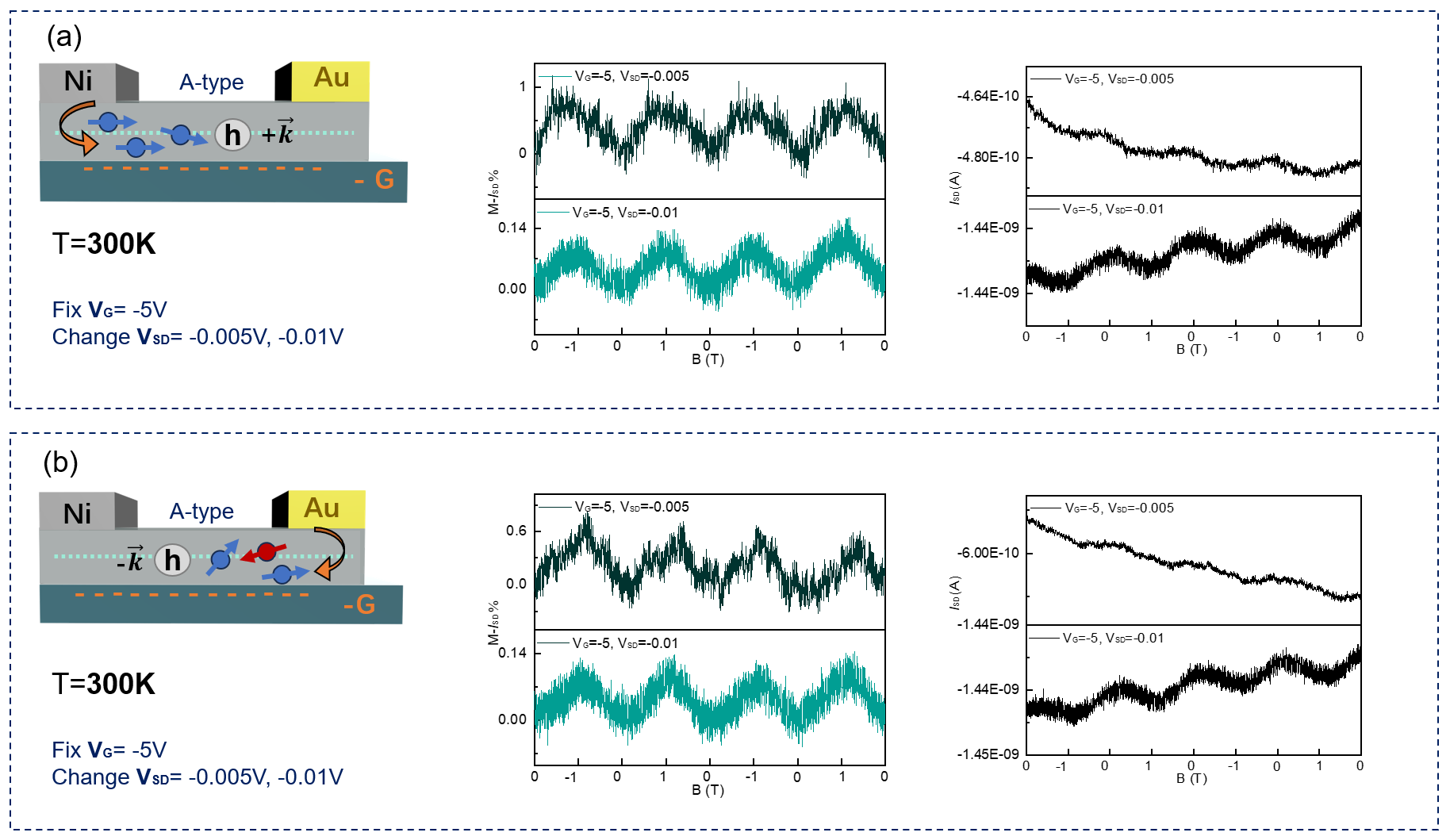


Figure S40: **(a)** **Left part**: Schematic diagram of hole transport in a FET device injected from Ni electron, the direction of the magnetic field is parallel to the direction of carrier transport. **Middle part**: The percentage change in the current of the FET with different source voltage (V_SD_) as a function of the magnetic field. **Right part**: The original curve of the current as a function of the magnetic field. **(b)** **Left part**: Schematic diagram of hole transport in a FET device injected from Au electron, the direction of the magnetic field is parallel to the direction of carrier transport. **Middle part**: The percentage change in the current of the FET with different source voltage (V_SD_) as a function of the magnetic field. **Right part**: The original curve of the current as a function of the magnetic field.

The temperature is 300 K.


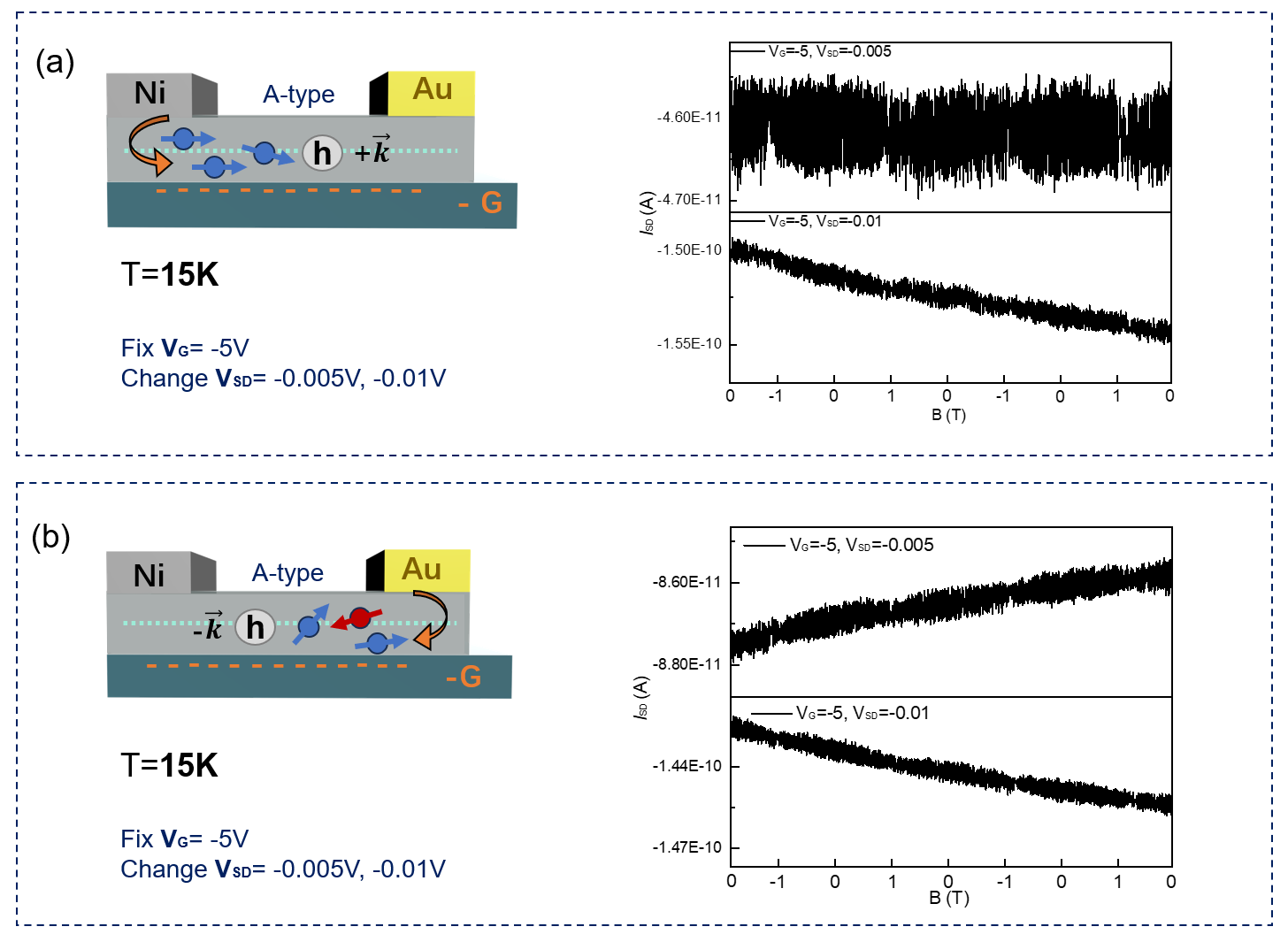


Figure S41: **(a)** **Left part**: Schematic diagram of hole transport in an FET device injected from Ni electron, the direction of the magnetic field is parallel to the direction of carrier transport. **Middle part**: The percentage change in the current of the FET with different source voltage (V_SD_) as a function of the magnetic field. **Right part**: The original curve of the current as a function of the magnetic field. **(b)** **Left part**: Schematic diagram of hole transport in a FET device injected from Au electron, the direction of the magnetic field is parallel to the direction of carrier transport. **Right part**: The original curve of the current as a function of the magnetic field. The temperature is 15 K.

**6 Dynamical chiral signal change of the OFET device with gate regulation under laser illumination**

From Figures S42 to S49, a series of CMC signal changes dynamically with current intensity and temperature under illumination are shown (10μm channel).

**Figure S42-S45, R-type device**

This part of the supplementary material demonstrates the CMC effect of the R-type FET device under illumination, including the change with gate and source voltage, and the response to different polarized lights. The wavelength of the laser is 793 nm. During all tests, the direction of the magnetic field is kept collinear with the direction of carrier transport.


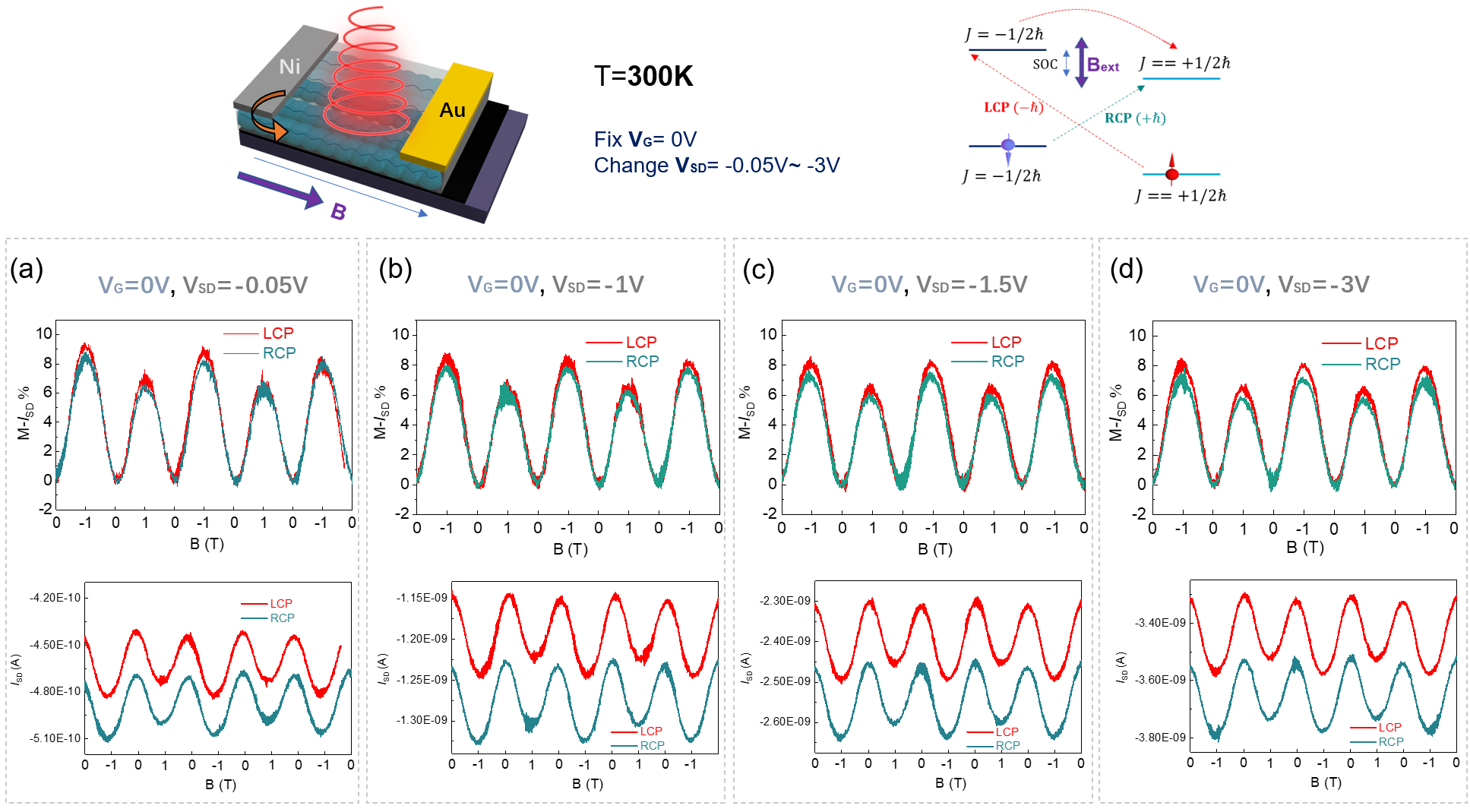


Figure S42: R-type device**,** the gate voltage is fixed at 0V, with the source voltage changed. In all the figures, the top part represents the percentage change in the current of the device with a magnetic field under polarized light, and the bottom part represents the raw data of the current change with the magnetic field under polarized light. **(a)** V_G_= 0 V, V_SD_= -0.05 V. **(b)** V_G_= 0 V, V_SD_= -1 V. **(c)** V_G_= 0 V, V_SD_= -1.5 V. **(d)** V_G_= 0 V, V_SD_= -3 V.

The temperature is 300 K.


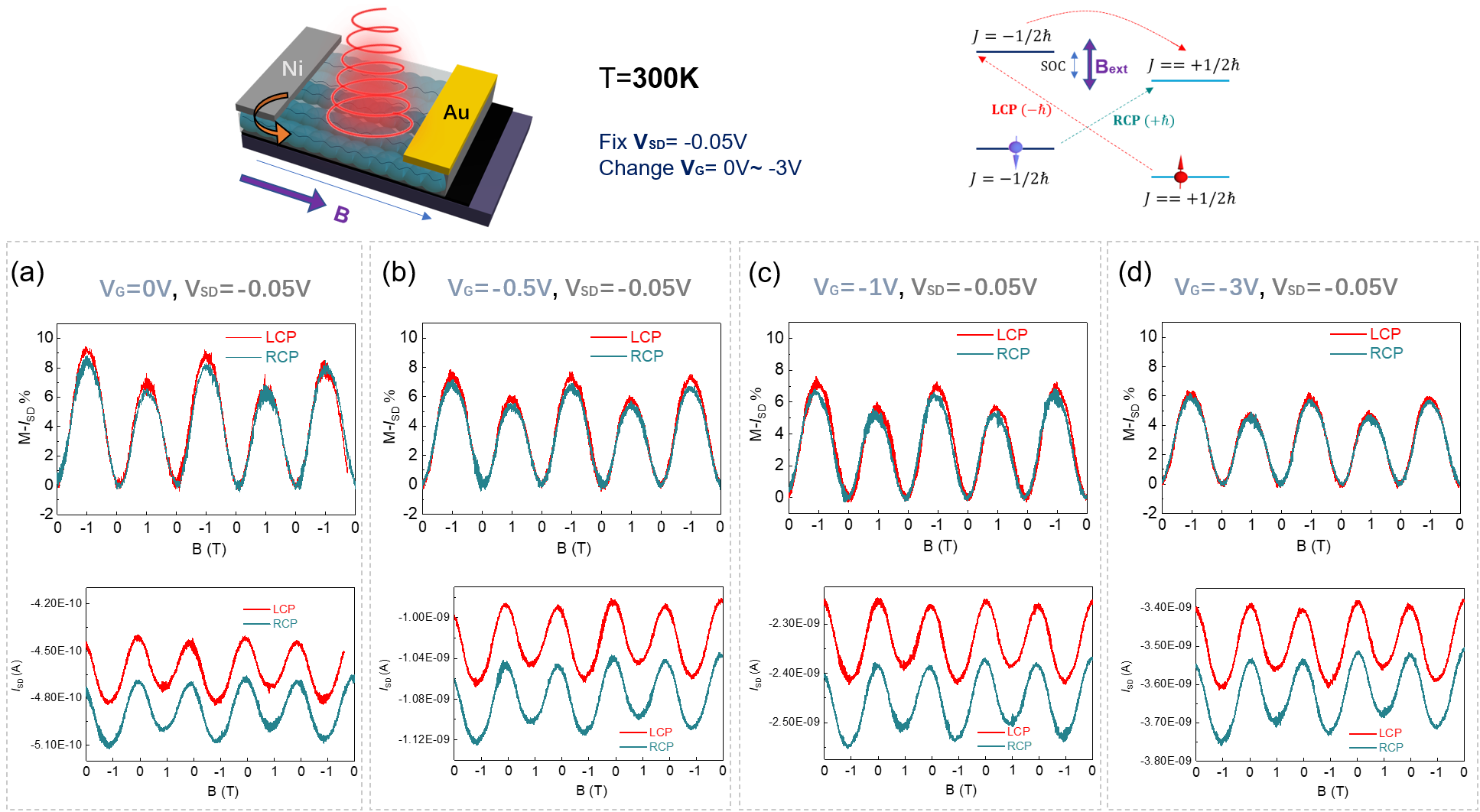


Figure S43: R-type device with Ni electrode acts as the carrier injection terminal**,** the source voltage is fixed at -0.05V, with the gate voltage changed. In all the figures, the top part represents the percentage change in the current of the device with a magnetic field under polarized light, and the bottom part represents the raw data of the current change with the magnetic field under polarized light. **(a)** V_G_= 0 V, V_SD_= -0.05 V. **(b)** V_G_= -0.5 V, V_SD_= -0.05 V. **(c)** V_G_= -1 V, V_SD_= -0.05 V. **(d)** V_G_= -3 V, V_SD_= -0.05 V.

The temperature is 300 K.


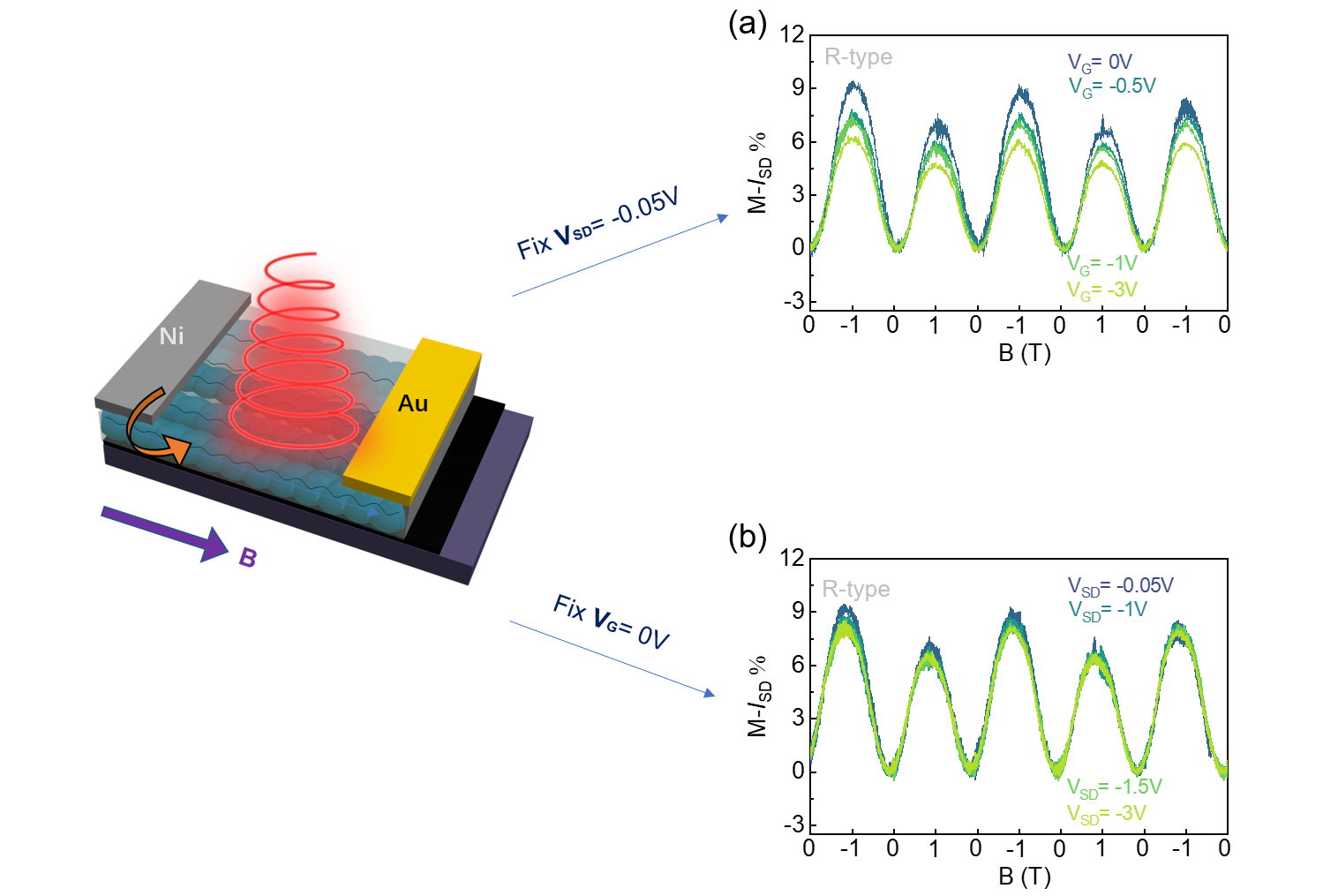


Figure S44: Comparison of the regulation of gate voltage and source voltage on the CMC effect in R-type device. **(a)** The source voltage is fixed at -0.05V, with the gate voltage changed from 0V to -3 V, The CMC strength and $\Delta g_{2}$ changed significantly. **(b)** the gate voltage is fixed at 0V, with the source voltage changed from -0.05 V to -3 V, The CMC strength and $\Delta g_{2}$ has no significant change.

The temperature is 300 K.


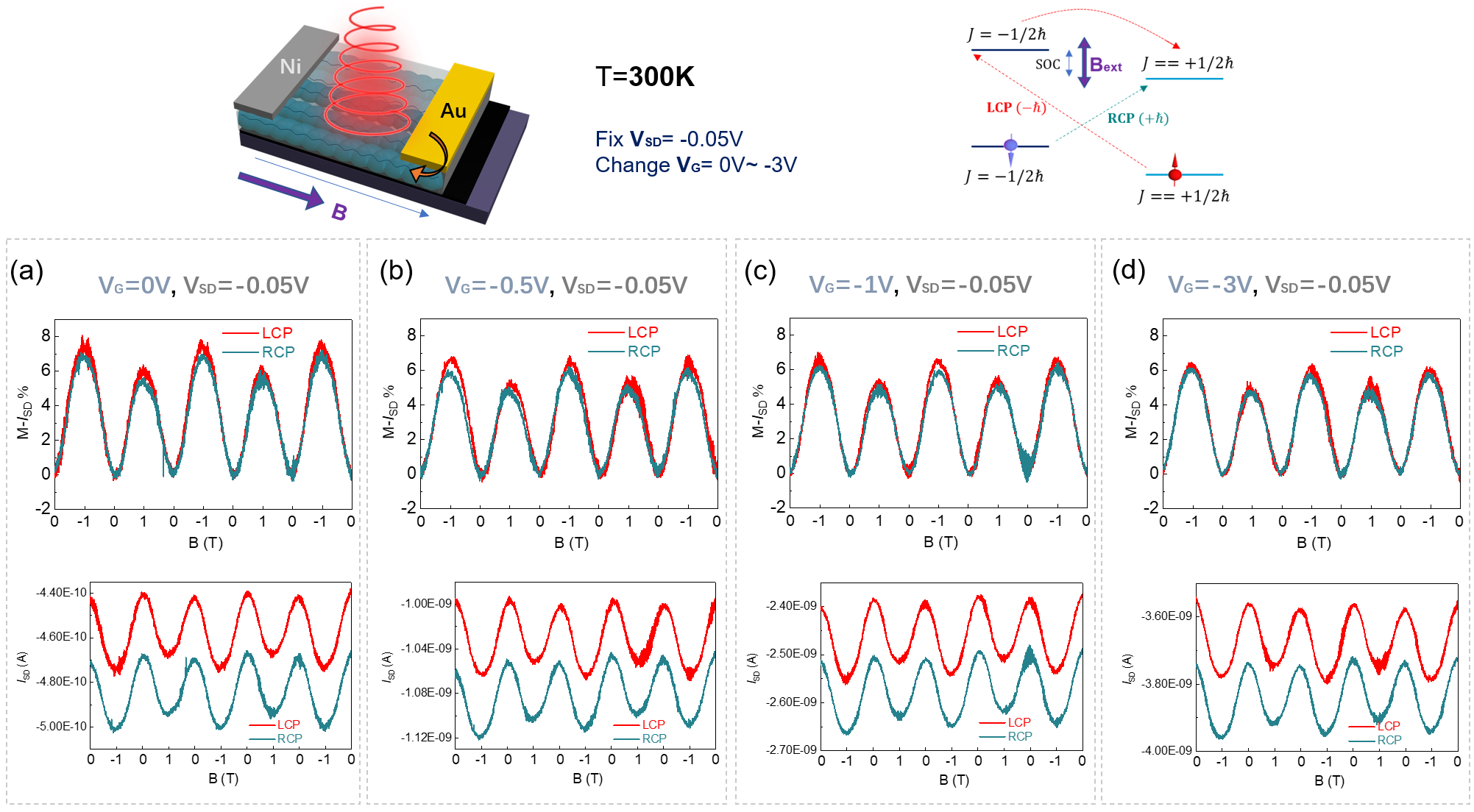


Figure S45: R-type device with Au electrode acts as the carrier injection terminal**,** the source voltage is fixed at -0.05V, with the gate voltage changed. In all the figures, the top part represents the percentage change in the current of the device with a magnetic field under polarized light, and the bottom part represents the raw data of the current change with the magnetic field under polarized light. **(a)** V_G_= 0 V, V_SD_= -0.05 V. **(b)** V_G_= -0.5 V, V_SD_= -0.05 V. **(c)** V_G_= -1 V, V_SD_= -0.05 V. **(d)** V_G_= -3 V, V_SD_= -0.05 V.

The temperature is 300 K.

**Figure S46-S49, S-type device**

This part of the supplementary material demonstrates the CMC effect of the S-type FET device under illumination, including the change with gate and source voltage, and the response to different polarized light. The wavelength of the laser is 793 nm. During all tests, the direction of the magnetic field is kept collinear with the direction of carrier transport.


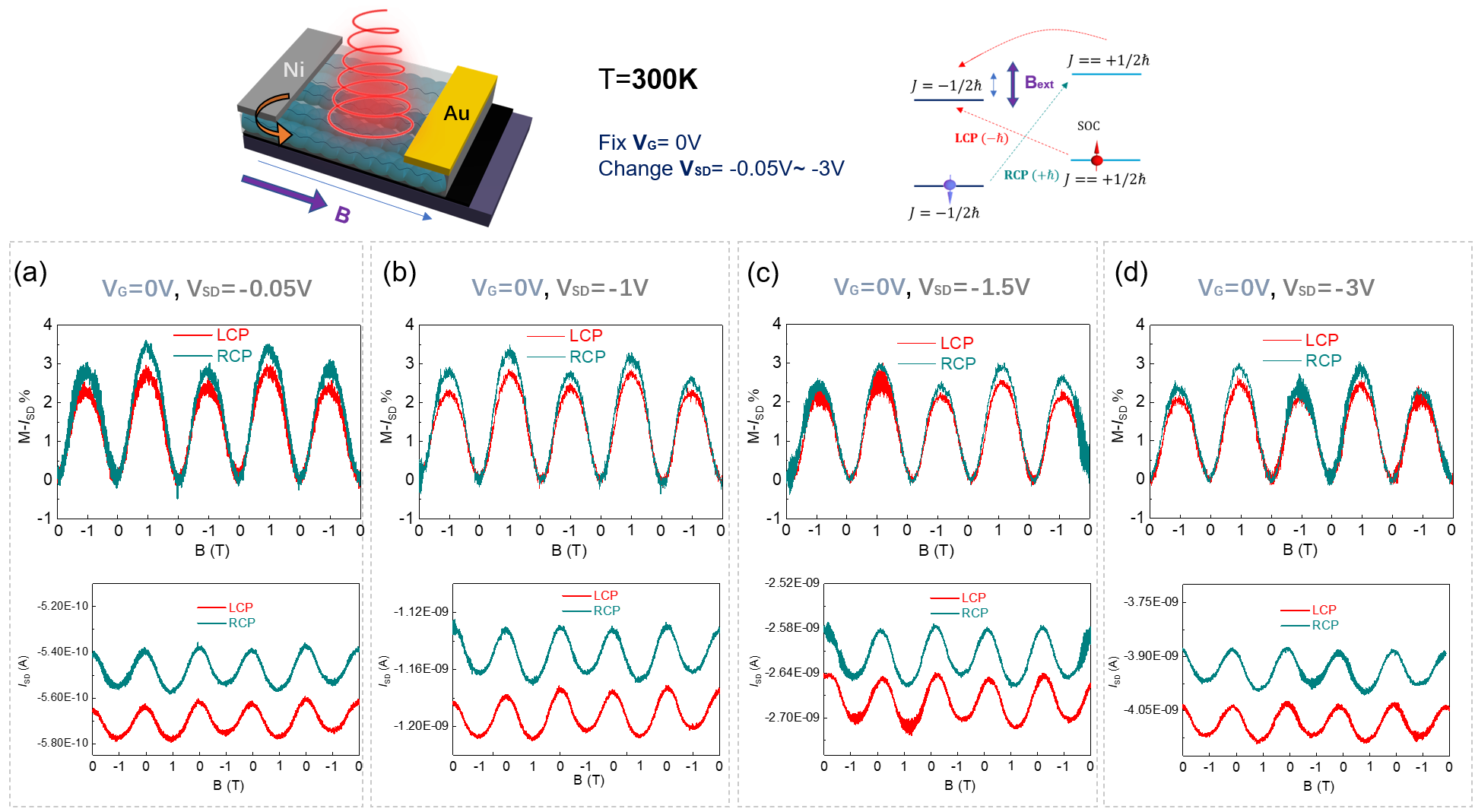


Figure S46: S-type device with Ni electrode acts as the carrier injection terminal**,** the gate voltage is fixed at 0V, with the source voltage changed. In all the figures, the top part represents the percentage change in the current of the device with magnetic field under polarized light, and the bottom part represents the raw data of the current change with the magnetic field under polarized light. **(a)** V_G_= 0 V, V_SD_= -0.05 V. **(b)** V_G_= 0 V, V_SD_= -1 V. **(c)** V_G_= 0 V, V_SD_= -1.5 V. **(d)** V_G_= 0 V, V_SD_= -3 V.

The temperature is 300 K.


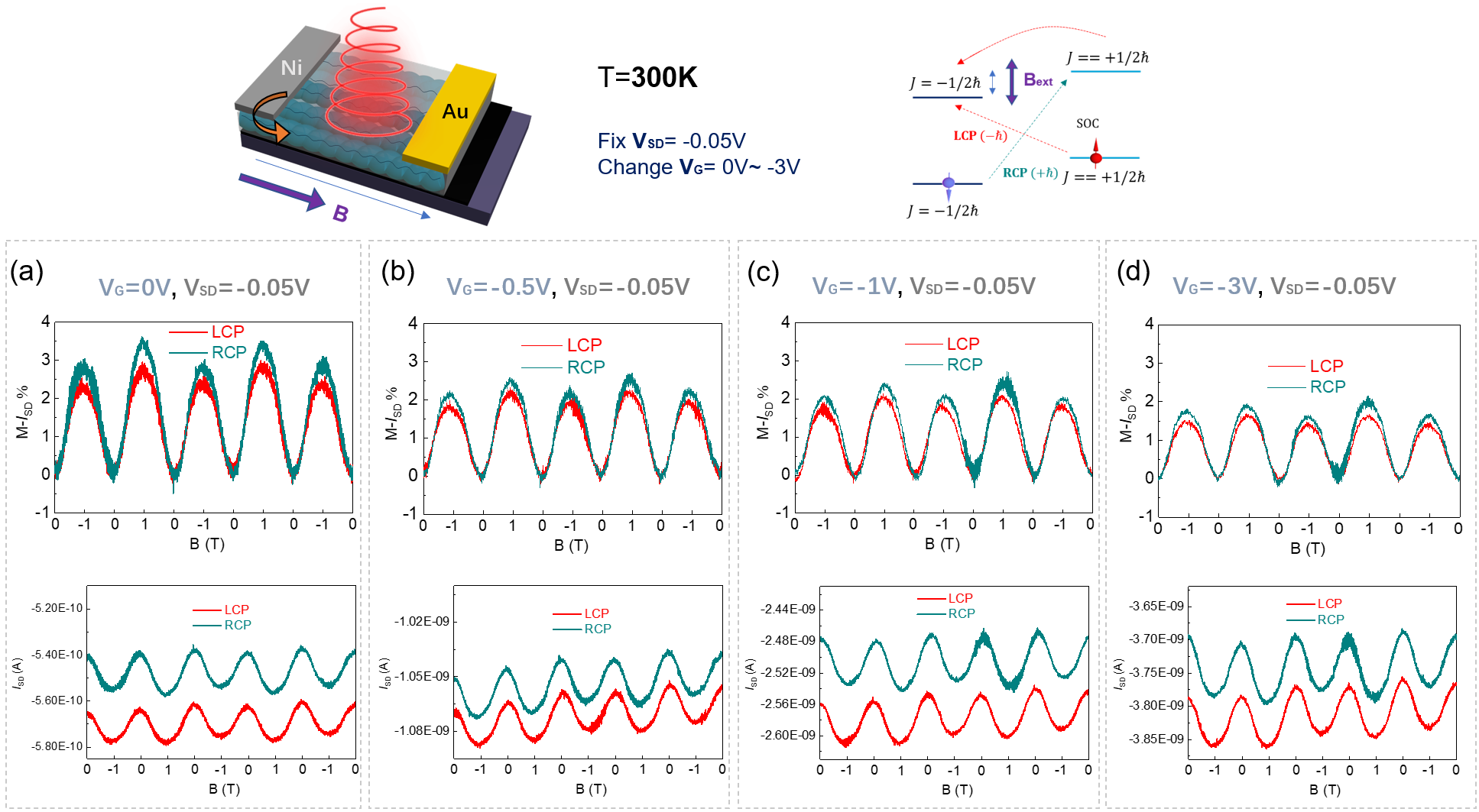


Figure S47: S-type device with Ni electrode acts as the carrier injection terminal**,** the source voltage is fixed at -0.05V, with the gate voltage changed. In all the figures, the top part represents the percentage change in the current of the device with magnetic field under polarized light, and the bottom part represents the raw data of the current change with the magnetic field under polarized light. **(a)** V_G_= 0 V, V_SD_= -0.05 V. **(b)** V_G_= -0.5 V, V_SD_= -0.05 V. **(c)** V_G_= -1 V, V_SD_= -0.05 V. **(d)** V_G_= -3 V, V_SD_= -0.05 V.

The temperature is 300 K.


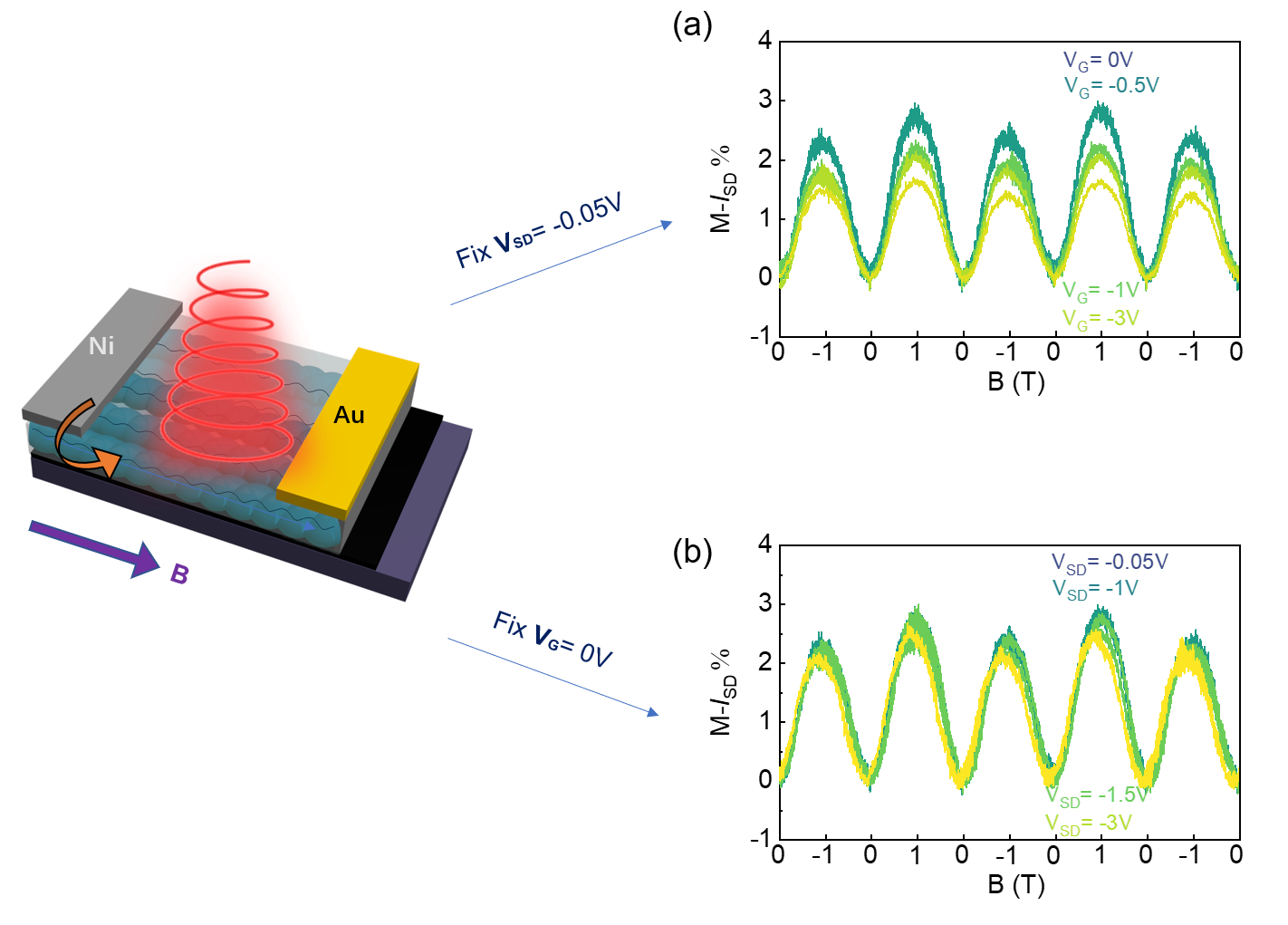


Figure S48: Comparison of the regulation of gate voltage and source voltage on the CMC effect in S-type device. **(a)** the source voltage is fixed at -0.05 V, with the gate voltage changed from 0 V to -3 V, The CMC strength and $\Delta g_{2}$ changed significantly. **(b)** the gate voltage is fixed at 0V, with the source voltage changed from -0.05V to -3 V, The CMC strength and $\Delta g_{2}$ has no significant change.

The temperature is 300 K.


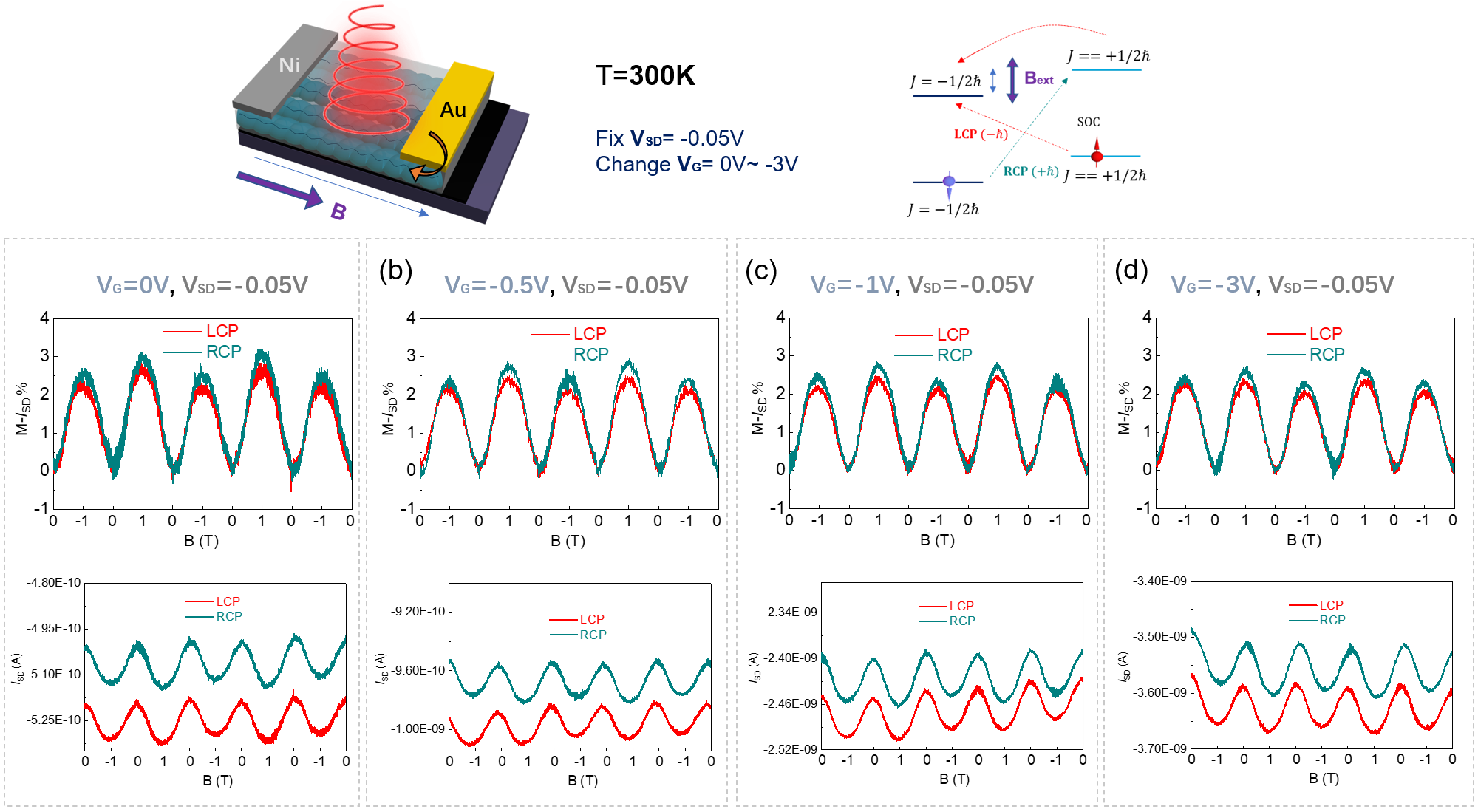


Figure S49: S-type device with Au electrode acts as the carrier injection terminal**,** the source voltage is fixed at -0.05V, with the gate voltage changed. In all the figures, the top part represents the percentage change in the current of the device with a magnetic field under polarized light, and the bottom part represents the raw data of the current change with the magnetic field under polarized light. **(a)** V_G_= 0 V, V_SD_= -0.05 V. **(b)** V_G_= -0.5 V, V_SD_= -0.05 V. **(c)** V_G_= -1 V, V_SD_= -0.05 V. **(d)** V_G_= -3 V, V_SD_= -0.05 V.

The temperature is 300 K.

**Figure S50-S52, Achiral-type device**

This part of the supplementary material demonstrates the MC effect of the **A-type** FET device under illumination, including the change with gate and source voltage, and the response to different polarized light. The wavelength of the laser is 793 nm. During all tests, the direction of the magnetic field is kept collinear with the direction of carrier transport.


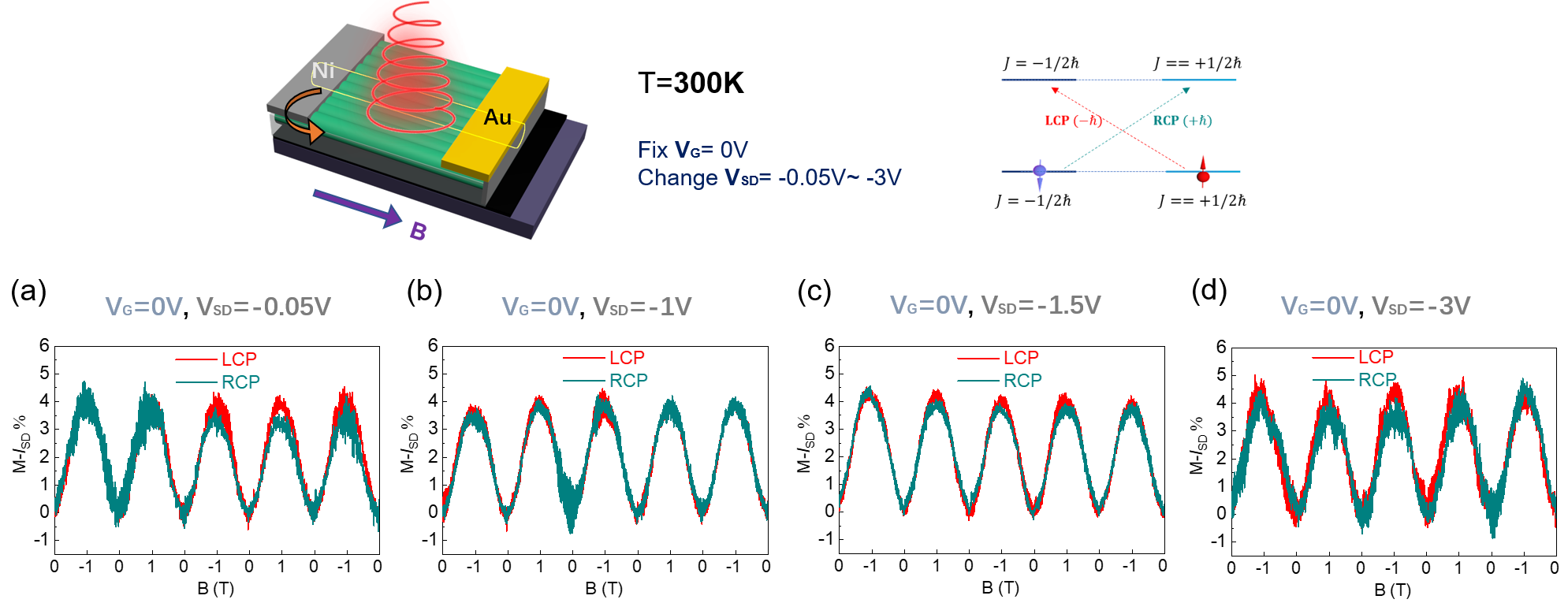


Figure S50: A-type device with Ni electrode acts as the carrier injection terminal**,** the gate voltage is fixed at 0V, with the source voltage changed. **(a)** V_G_= 0 V, V_SD_= -0.05 V. **(b)** V_G_= 0 V, V_SD_= -1 V. **(c)** V_G_= 0 V, V_SD_= -1.5 V. **(d)** V_G_= 0 V, V_SD_= -3 V.

The temperature is 300 K.


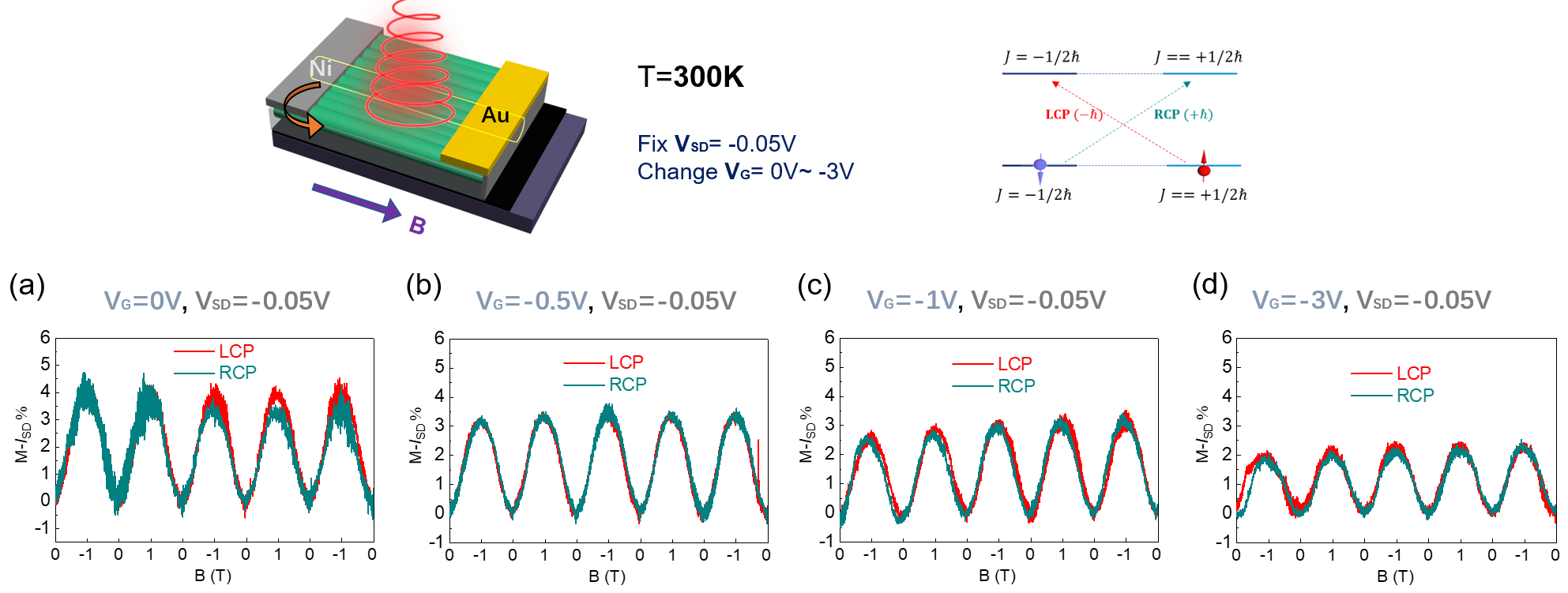


Figure S51: A-type device with Ni electrode acts as the carrier injection terminal**,** the source voltage is fixed at -0.05 V, with the gate voltage changed. **(a)** V_G_= 0 V, V_SD_= -0.05 V. **(b)** V_G_= -0.5 V, V_SD_= -0.05 V. **(c)** V_G_= -1 V, V_SD_= -0.05 V. **(d)** V_G_= -3 V, V_SD_= -0.05 V.

The temperature is 300 K.


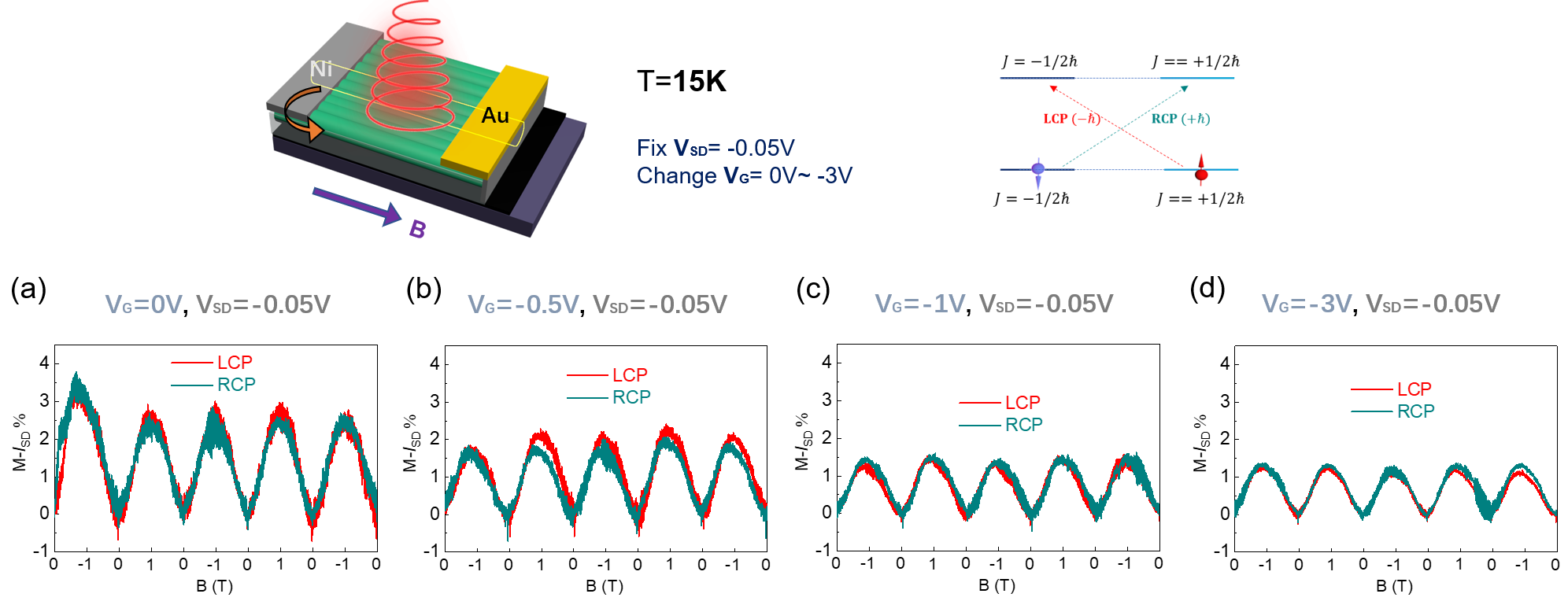


Figure S52: A-type device with Ni electrode acts as the carrier injection terminal**,** the source voltage is fixed at -0.05V, with the gate voltage changed. **(a)** V_G_= 0 V, V_SD_= -0.05 V. **(b)** V_G_= -0.5 V, V_SD_= -0.05 V. **(c)** V_G_= -1 V, V_SD_= -0.05 V. **(d)** V_G_= -3 V, V_SD_= -0.05 V.

The temperature is 15 K.

**Figures S53-S55**

This part of the supplementary material demonstrates the MC effect of the R, S and A-type FET device under illumination, including the change with gate and source voltage, and the response to right-circularly polarized light. The wavelength of the laser is 793nm. During all tests, the direction of the magnetic field is kept perpendicular to the direction of carrier transport.


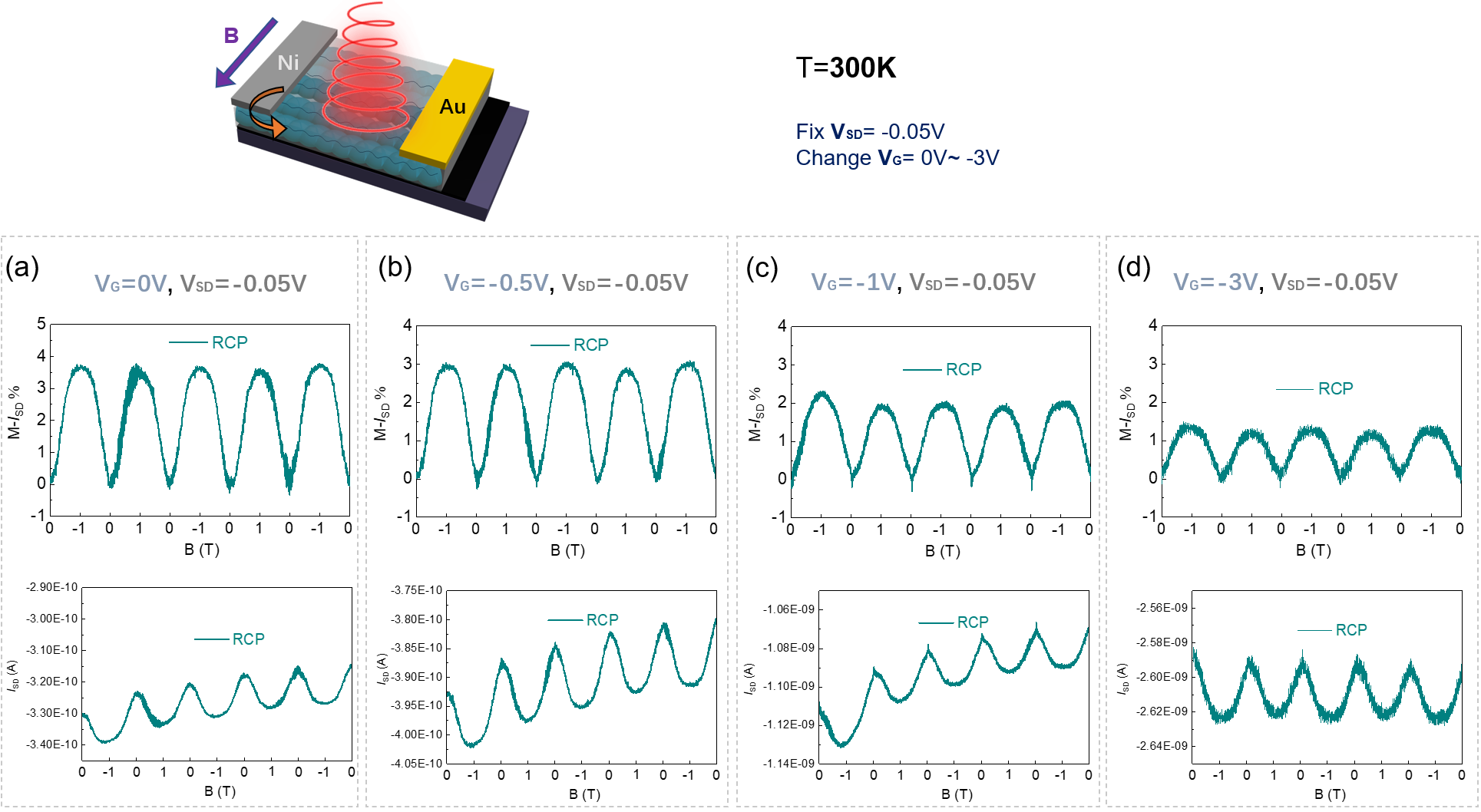


Figure S53: R-type device with Ni electrode acts as the carrier injection terminal**,** the source voltage is fixed at -0.05V, with the gate voltage changed. In all the figures, the top part represents the percentage change in the current of the device with a magnetic field under polarized light, and the bottom part represents the raw data of the current change with the magnetic field under polarized light. **(a)** V_G_= 0 V, V_SD_= -0.05 V. **(b)** V_G_= -0.5 V, V_SD_= -0.05 V. **(c)** V_G_= -1 V, V_SD_= -0.05 V. **(d)** V_G_= -3 V, V_SD_= -0.05 V.

The temperature is 300 K.


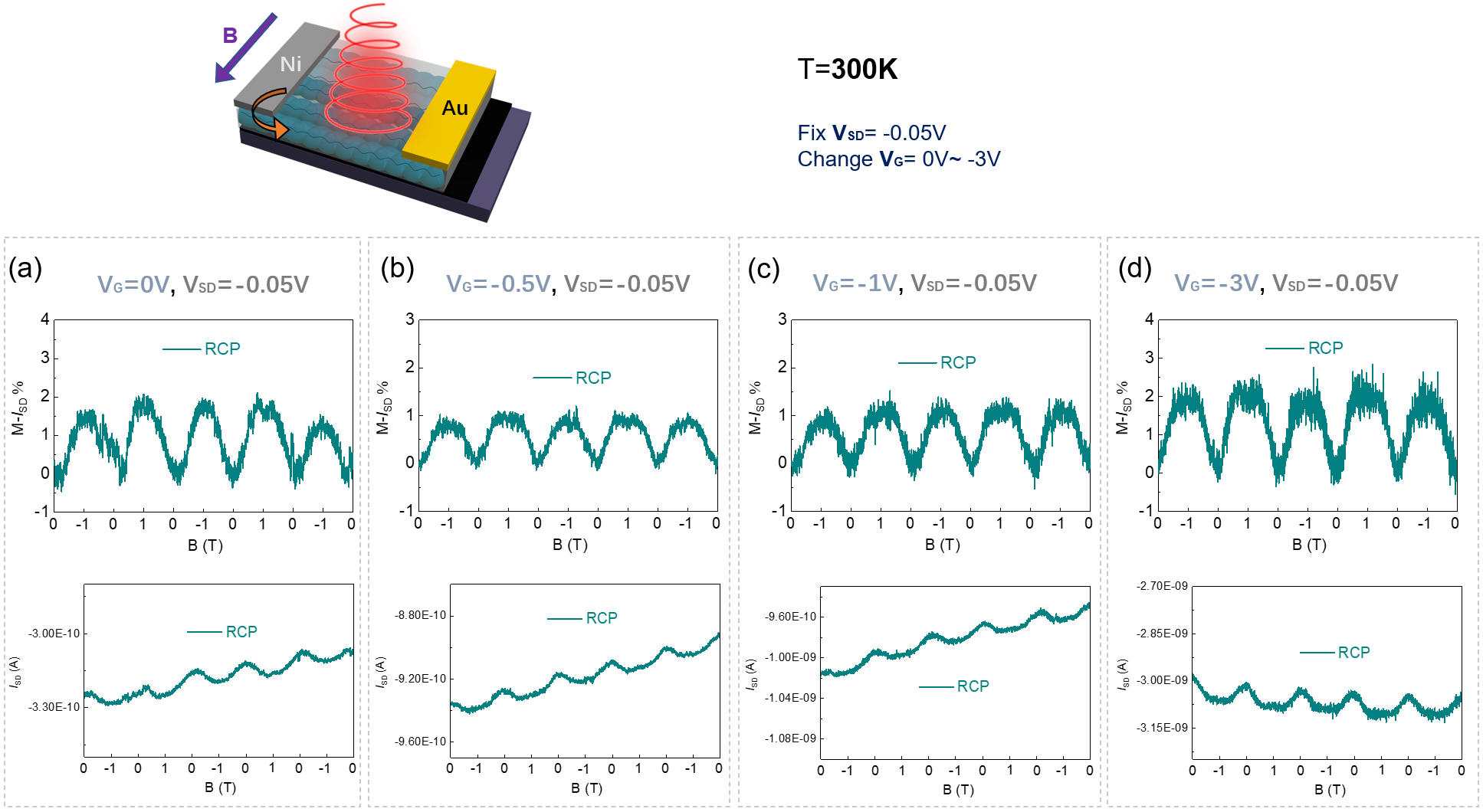


Figure S54: S-type device with Ni electrode acts as the carrier injection terminal**,** the source voltage is fixed at -0.05 V, with the gate voltage changed. In all the figures, the top part represents the percentage change in the current of the device with a magnetic field under polarized light, and the bottom part represents the raw data of the current change with the magnetic field under polarized light. **(a)** V_G_= 0 V, V_SD_= -0.05 V. **(b)** V_G_= -0.5 V, V_SD_= -0.05 V. **(c)** V_G_= -1 V, V_SD_= -0.05 V. **(d)** V_G_= -3 V, V_SD_= -0.05 V.

The temperature is 300 K.


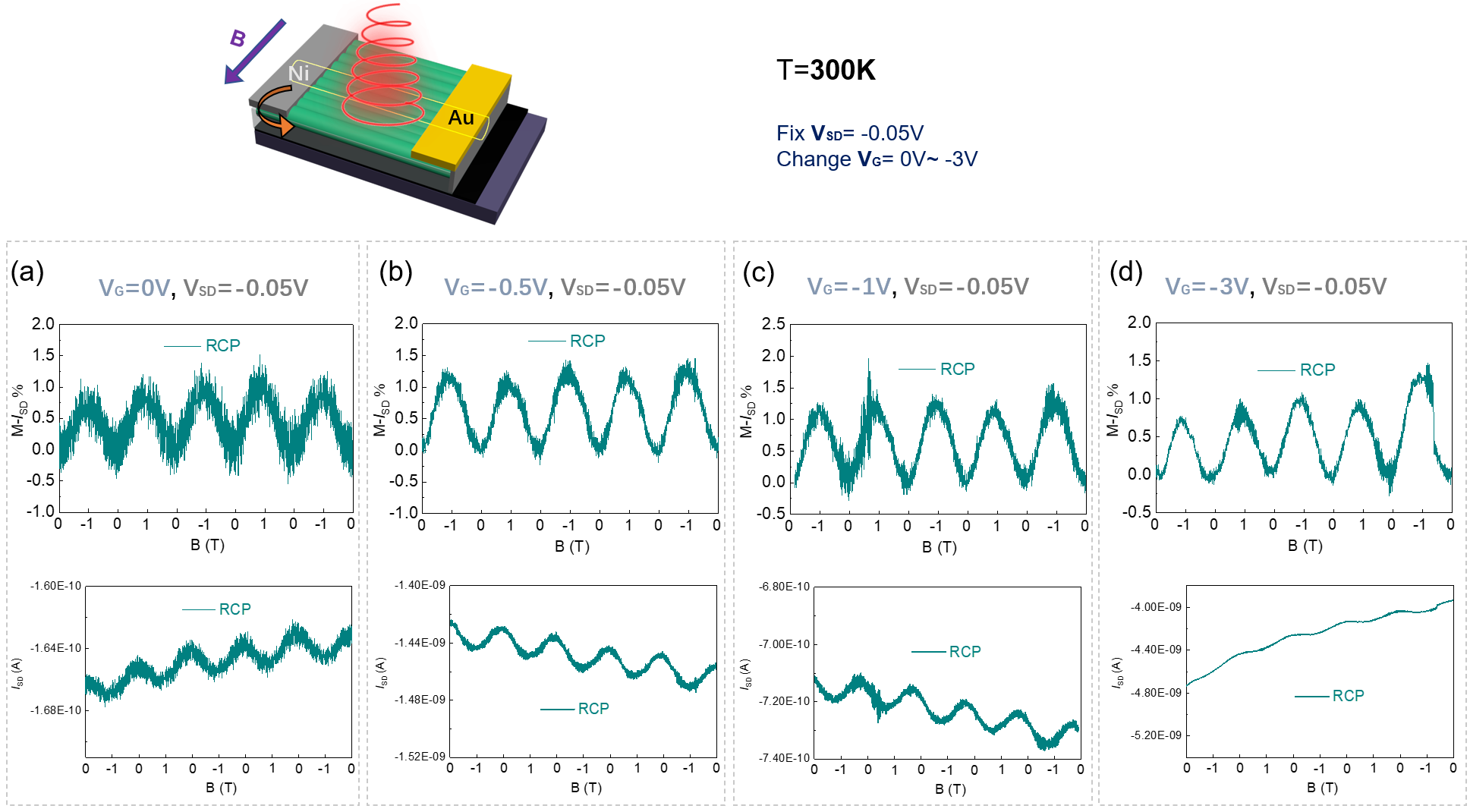


Figure S55: A-type device with Ni electrode acts as the carrier injection terminal**,** the source voltage is fixed at -0.05V, with the gate voltage changed. In all the figures, the top part represents the percentage change in the current of the device with a magnetic field under polarized light, and the bottom part represents the raw data of the current change with the magnetic field under polarized light. **(a)** V_G_= 0 V, V_SD_= -0.05 V. **(b)** V_G_= -0.5 V, V_SD_= -0.05 V. **(c)** V_G_= -1 V, V_SD_= -0.05 V. **(d)** V_G_= -1.5 V, V_SD_= -0.05 V.

The temperature is 300 K.

**7 The transmission limit of the chiral signal under laser illumination**

In the previous content, we compared the chiral signal transport in the dark state and under illumination. Benefiting from the synergistic effect of selective transition of electrons under light excitation and chiral-induced spin-selective transport, the chiral signal transport is not significantly attenuated at the typical transmission scale (10-60 μm). To further explore the limit distances of chiral signal transport, we prepare OFET devices at longer transmission scales. As shown in Figure S56, the OFET devices can exhibit normal transfer characteristics at different channel lengths (100-900 μm). Due to the sharp increase in the distance between the source and drain electrodes (an increase in resistance), a slight increase in the on-V_G_ and a significant decrease in the I_SD_ are correspondingly observed.

Herein, we use the same test method as before. Adjusting V_G_ and V_SD_ to reduce current drift and stabilize the current density in a similar range, and then test the current change with the magnetic field under light excitation (LCP and RCP). In this process, the external magnetic field direction remains parallel to the current direction. Significantly, at long-scale transport distances (even up to 900 μm), the asymmetry of the CMC signal refers to the different magnetic field direction can be well maintained. To some extent, in chiral materials, the CMC signals under light excitation shown in OFET, the differential response of photoluminescence to CPL shown in raw material, and the differential response of current intensity to CPL shown in photodetector, can be classified into similar categories. In the above case, as long as the surface of the chiral material is covered by CPL, the chiral features can be read out with specific signals, which correspondingly weakens the limitation of the transmission distance of electrical signals. Besides, we did not continue to amplify the transmission distance, because millimeter-level and above planar devices are beyond the scope of traditional field-effect devices. The current channel scale can fully demonstrate the potential of our OFET devices in chiral signal transmission under illumination.


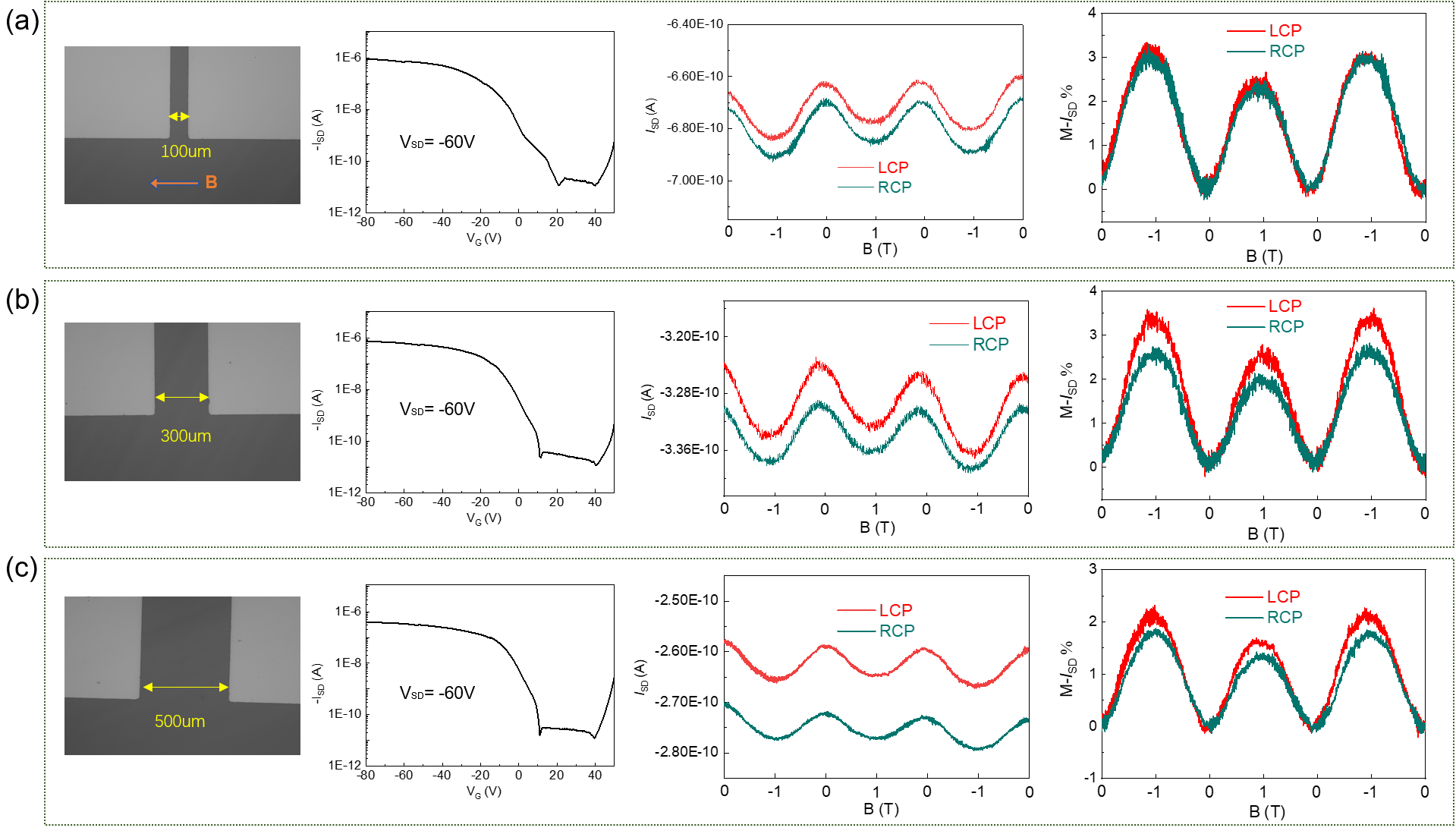

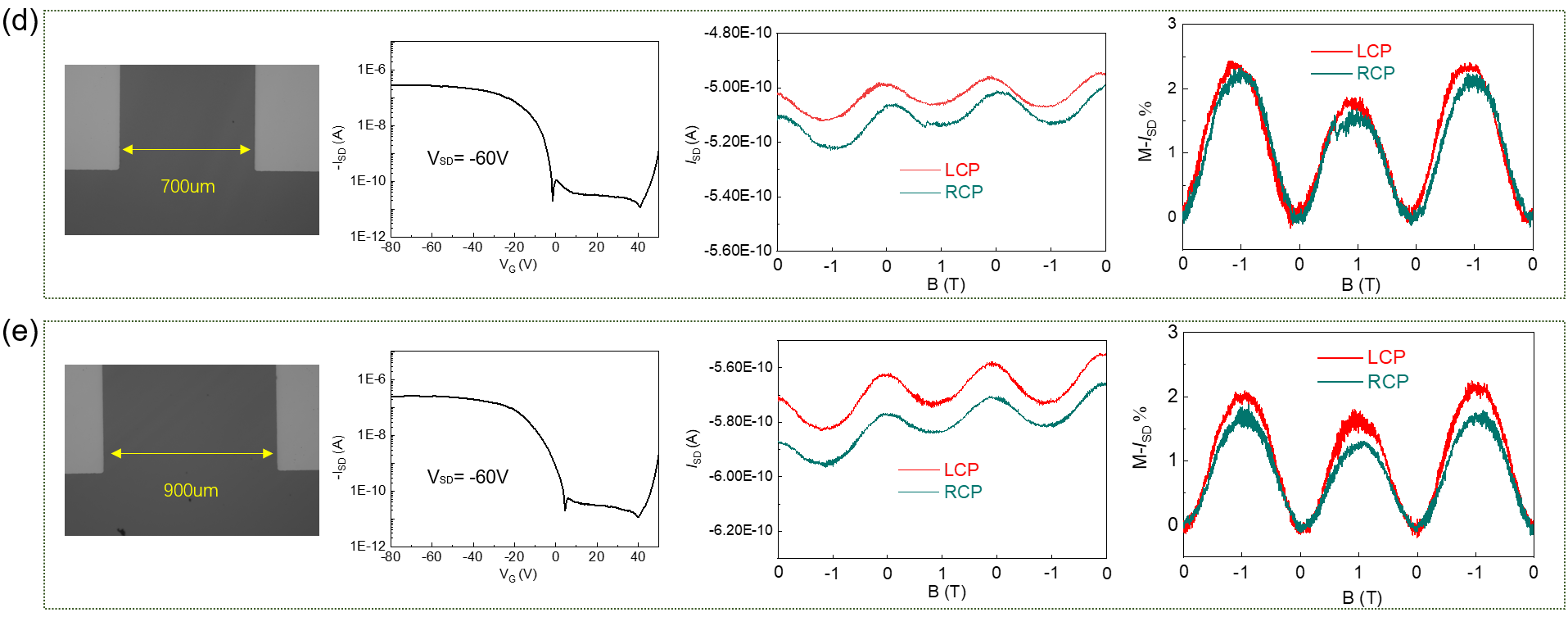


Figure S56: The chiral signals transmission under photoexcitation with different channels in 300 K. All the OFET devices are fabricated with R-type polymer. In each subgraph, from left to right, is the optical picture of the OFET device, the transfer characteristic curve, the raw current data of the chiral signal, and the change ratio of the chiral signal. To suppress current drift and stabilize the current density, the gate and source voltages are slightly adjusted at each channel length. **(a)** d=100 μm, V_G_= -1.5 V, V_SD_= -0.5 V. **(b)** d=300 μm, V_G_= -2 V, V_SD_= -0.5 V. **(c)** d=500 μm, V_G_= -2 V, V_SD_= -0.8 V. **(d)** d=700 μm, V_G_= -3 V, V_SD_= -0.5 V. **(e)** d=900 μm, V_G_= -3 V, V_SD_= -1 V.

**8 Stability of chiral structures and devices**


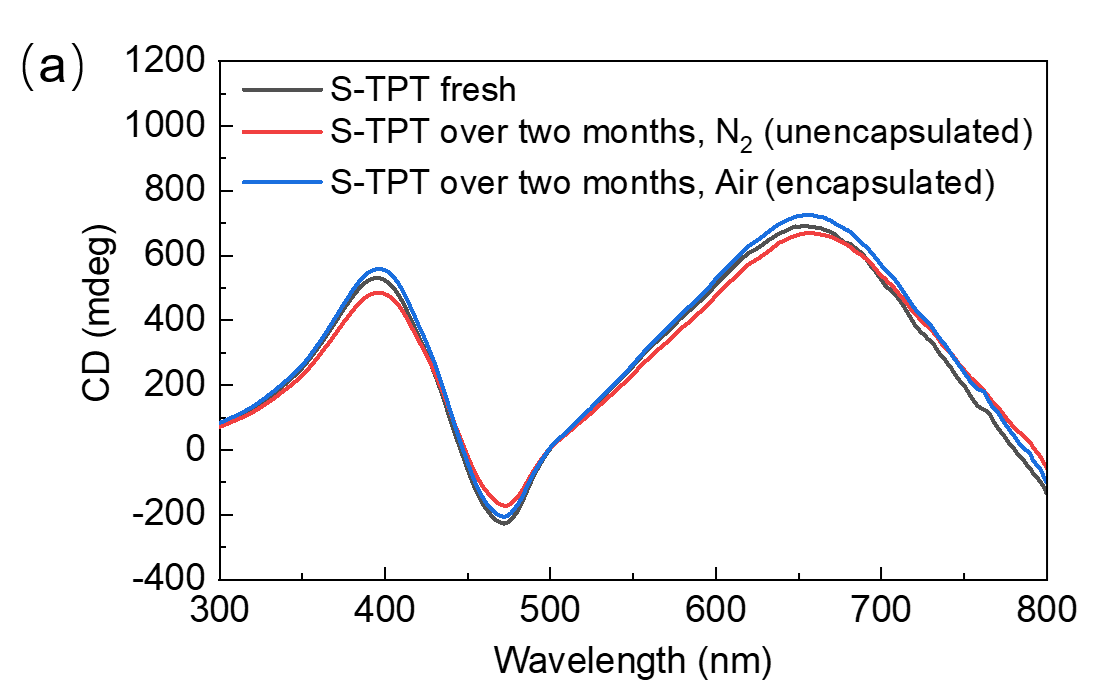


Figure S57**.** The stability testing of chiral structures. Here, we provide data for the fresh sample (black curve), the sample left unencapsulated in a nitrogen glove box for two months (red curve), and the sample left encapsulated in air for two months.


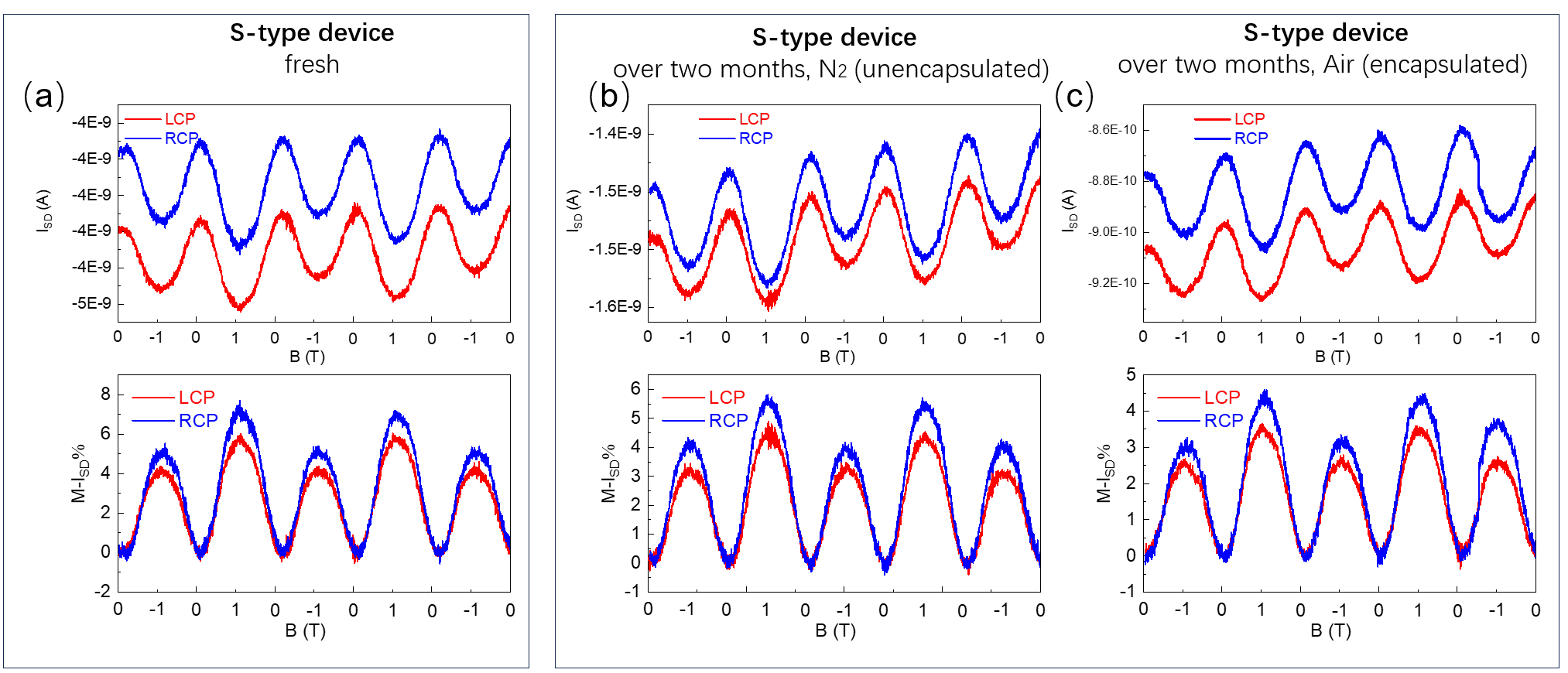


Figure S58**.** The stability testing of chiral devices. Here, we provide data for a fresh chiral device **(a)**. chiral device left unencapsulated in a nitrogen glove box for two months **(b),** and a chiral device left encapsulated in air for two months **(c).** The upper part represents the original current data, and the down part is the calculated CMC data. The voltage parameters are V_G_= -1 V, V_SD_= -0.05 V in room temperature for all the devices.

**Reference**

1. Su, W. P., Schrieffer, J. R., Heeger, A. J. *Phys. Rev. Lett.* **42**, 1698 (1979).

2. Johansson, A. A., S. Stafström, S. *Phys. Rev. B* **69**, 235205 (2004).

3. Mu, X. Y., Ji, Y. W., Yin, H., Gao, K. *Phys. Rev. Applied* **19**, 054017 (2023).

4. Heeger, A. J., Kivelson, S., Schrieffer, J. R., Su, W. P. *Rev. Mod. Phys.* **60**, 781 (1988).

5. Li, C., Li, Y., Xu, L. X., Meng, R. X., Gao, K. *J. Phys. Chem. C* **124**, 1898 (2020).

6. Shi, X. Y., Zhang, M. M., Wang, W. J., Gao, K. *Appl. Phys. Lett.* **119**, 263302 (2021).

7. Yuan, Y., Giri, G., Ayzner, AL., Zoombelt, AP., Mannsfeld, SC., Chen, J. et al. Ultra-high mobility transparent organic thin film transistors grown by an off-centre spin-coating method. *Nat. Commun.* **5**, (2014).

8. Jia, L., Wang, C., Zhang, Y., Yang, L., Yan, Y. Efficient Spin Selectivity in Self-Assembled Superhelical Conducting Polymer Microfibers. *ACS Nano* **14**, 6607-6615 (2020).

9. Das, TK., Naaman, R., Fransson, J. Insights into the Mechanism of Chiral-Induced Spin Selectivity: The Effect of Magnetic Field Direction and Temperature. *Adv. Mater.* **36**, e2313708 (2024).

10. Qian, Q., Ren, H., Zhou, J., Wan, Z., Zhou, J., Yan, X. et al. Chiral molecular intercalation superlattices. *Nature* **606**, 902-908 (2022).

11. Yang, S-H., Naaman, R., Paltiel, Y., Parkin, SSP. Chiral spintronics. *Nat. Rev. Phys.* **3**, 328-343 (2021).

12. Guo, L., Gu, X., Hu, S., Sun, W., Zhang, R., Qin, Y. et al. Strain-restricted transfer of ferromagnetic electrodes for constructing reproducibly superior-quality spintronic devices. *Nat. Commun.* **15**, 865 (2024).

13. Meng, K., Li, M., Guo, L., Zhang, R., Guo, A., Liu, M. et al. Room-Temperature Organic Spintronic Devices with Wide Range Magnetocurrent Tuning and Multifunctionality via Electro-Optical Compensation Strategy. *Adv. Mater.* **37**, e2417995 (2025).

14. Shiota, K., Inui, A., Hosaka, Y., Amano, R., Onuki, Y., Hedo, M. et al. Chirality-Induced Spin Polarization over Macroscopic Distances in Chiral Disilicide Crystals. *Phys. Rev. Lett.* **127**, 126602 (2021).

15. Nakajima, R., Hirobe, D., Kawaguchi, G., Nabei, Y., Sato, T., Narushima, T. et al*.* Giant spin polarization and a pair of antiparallel spins in a chiral superconductor. *Nature* **613**, 479-484 (2023).

16. Bian, Z., Nakano, Y., Miyata, K., Oya, I., Nobuoka, M., Tsutsui, Y. et al. Chiral Van Der Waals Superlattices for Enhanced Spin-Selective Transport and Spin-Dependent Electrocatalytic Performance. *Adv. Mater.* **35**, e2306061 (2023).

17. Suda, M., Thathong, Y., Promarak, V., Kojima, H., Nakamura, M., Shiraogawa, T. et al*.* Light-driven molecular switch for reconfigurable spin filters. *Nat. Commun.* **10**, 2455 (2019).

18. Aizawa, H., Sato, T., Maki-Yonekura, S., Yonekura, K., Takaba, K., Hamaguchi, T. et al. Enantioselectivity of discretized helical supramolecule consisting of achiral cobalt phthalocyanines via chiral-induced spin selectivity effect. *Nat. Commun.* **14**, 4530 (2023).

19. Mondal, AK., Preuss, MD., Sleczkowski, ML., Das, TK., Vantomme, G., Meijer, EW. et al. Spin Filtering in Supramolecular Polymers Assembled from Achiral Monomers Mediated by Chiral Solvents. *J. Am. Chem. Soc.* **143**, 7189-7195 (2021).

20. Sun, X. et al. A molecular spin-photovoltaic device. *Science* **357**, 677-680 (2017).
